# Mapping genetic variants for cranial vault shape in humans

**DOI:** 10.1371/journal.pone.0196148

**Published:** 2018-04-26

**Authors:** Jasmien Roosenboom, Myoung Keun Lee, Jacqueline T. Hecht, Carrie L. Heike, George L. Wehby, Kaare Christensen, Eleanor Feingold, Mary L. Marazita, A. Murat Maga, John R. Shaffer, Seth M. Weinberg

**Affiliations:** 1 Center for Craniofacial and Dental Genetics, Department of Oral Biology, University of Pittsburgh, Pittsburgh, PA, United States of America; 2 Department of Pediatrics, University of Texas McGovern Medical Center, Houston, TX, United States of America; 3 Department of Pediatrics, Seattle Children’s Craniofacial Center, University of Washington, Seattle, WA, United States of America; 4 Department of Health Management and Policy, University of Iowa, Iowa City, IA, United States of America; 5 Department of Epidemiology, Institute of Public Health, University of Southern Denmark, Odense, Denmark; 6 Department of Human Genetics, University of Pittsburgh, Pittsburgh, PA, United States of America; 7 Center for Developmental Biology and Regenerative Medicine, Seattle Children’s Research Institute Seattle, WA, United States of America; 8 Department of Anthropology, University of Pittsburgh, Pittsburgh, PA, United States of America; Medical University of South Carolina, UNITED STATES

## Abstract

The shape of the cranial vault, a region comprising interlocking flat bones surrounding the cerebral cortex, varies considerably in humans. Strongly influenced by brain size and shape, cranial vault morphology has both clinical and evolutionary relevance. However, little is known about the genetic basis of normal vault shape in humans. We performed a genome-wide association study (GWAS) on three vault measures (maximum cranial width [MCW], maximum cranial length [MCL], and cephalic index [CI]) in a sample of 4419 healthy individuals of European ancestry. All measures were adjusted by sex, age, and body size, then tested for association with genetic variants spanning the genome. GWAS results for the two cohorts were combined via meta-analysis. Significant associations were observed at two loci: 15p11.2 (lead SNP rs2924767, p = 2.107 × 10^−8^) for MCW and 17q11.2 (lead SNP rs72841279, p = 5.29 × 10^−9^) for MCL. Additionally, 32 suggestive loci (p < 5x10^-6^) were observed. Several candidate genes were located in these loci, such as *NLK*, *MEF2A*, *SOX9* and *SOX11*. Genome-wide linkage analysis of cranial vault shape in mice (N = 433) was performed to follow-up the associated candidate loci identified in the human GWAS. Two loci, 17q11.2 (c11.loc44 in mice) and 17q25.1 (c11.loc74 in mice), associated with cranial vault size in humans, were also linked with cranial vault size in mice (LOD scores: 3.37 and 3.79 respectively). These results provide further insight into genetic pathways and mechanisms underlying normal variation in human craniofacial morphology.

## Introduction

The dimensions of the human cranial vault, which forms the protective skeletal covering around the brain [1], are highly heritable [2]. While mutations in a number of genes are known to affect either brain growth [3] and/or the timing of cranial suture fusion [4] and result in an altered vault shape, less is known about the genetic basis of normal-range variation of this portion of the craniofacial complex. One reason the genetic basis of normal cranial vault shape has received relatively little attention may be that, unlike the face, the dimensions of the vault are not easily captured with photogrammetry. An improved understanding of the genetic basis of normal-range cranial vault morphology may provide novel insights into the biological pathways underlying both normal and abnormal cranial development. For instance, it may explain some of the phenotypic variability observed clinically in cases of craniosynostosis [4], a condition in which the vault shape is altered due to the premature fusion of one or more cranial sutures. Using traditional anthropometry, two prior candidate gene studies [5,6] reported associations between cranial vault shape and common polymorphisms in the *FGFR1* gene, chosen because mutations in this gene have been implicated in craniosynostosis syndromes. While these studies were promising, a large-scale genome-wide investigation of normal cranial vault morphology had been lacking.

To address this deficit, we examined the genetic basis of cranial vault morphology in 4419 individuals of recent European ancestry by performing genome-wide association studies (GWASs) of maximum cranial width (MCW), maximum cranial length (MCL), and the cephalic index (CI) in two independent cohorts. Subsequently, genome-wide significant and suggestive loci were tested using linkage analysis in a sample of mice with comparable cranial vault measurements available.

## Results

### Association studies

GWAS was performed for MCW, MCL and CI with 968515 (for the 3DFN cohort) or 567677 (for the OFC cohort) genotyped and imputed SNPs with MAF > 1% in two separate European-derived cohorts using either linear regression or mixed models. GWAS results for each cohort were then combined via inverse variance-weighted meta-analysis using Stouffer’s p-value method [7]. The conventional threshold of 5x10^-8^ was set for genome-wide statistical significance.

**Fig 1:**
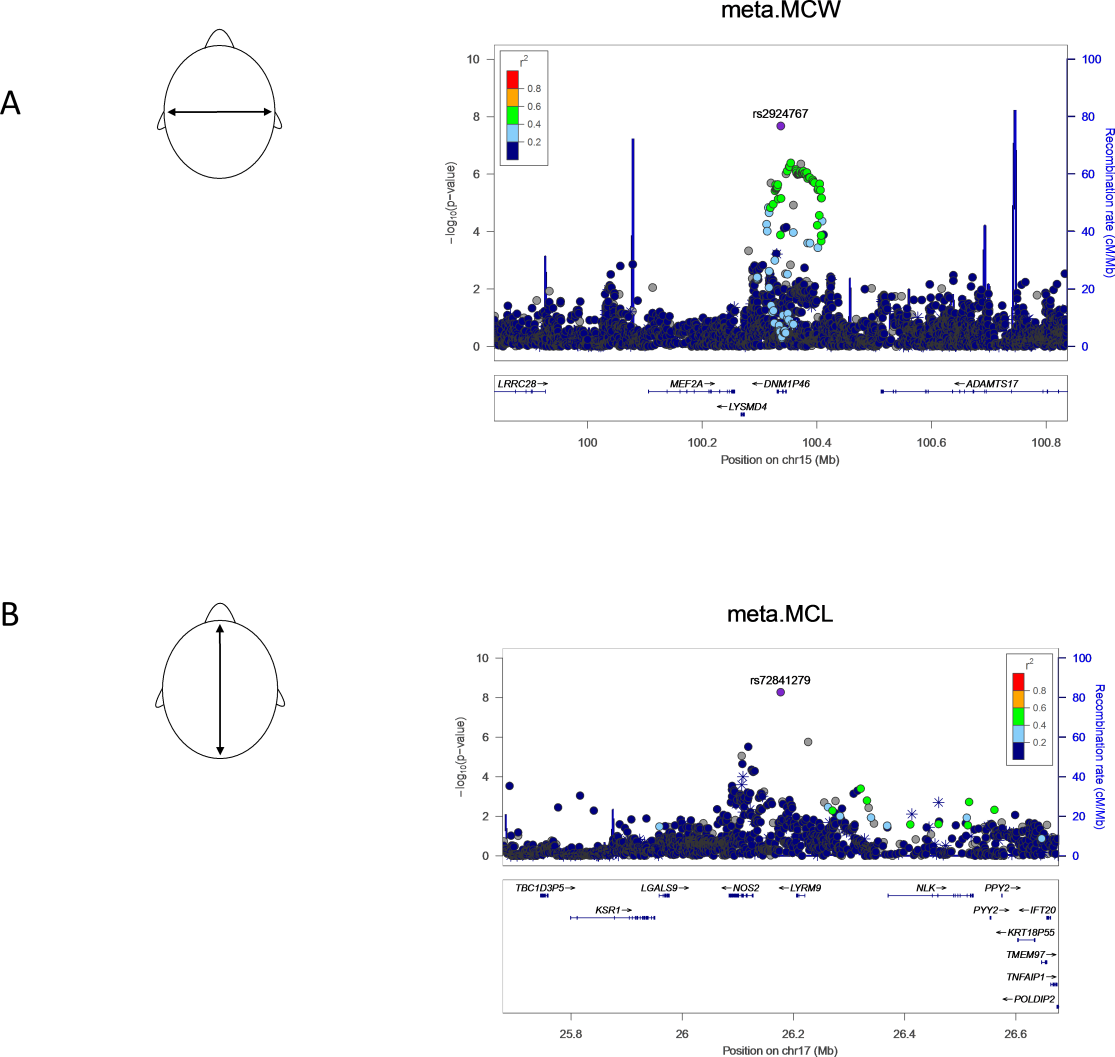
LocusZoom plots showing genome-wide significant associations observed in the meta- analysis. (A) The observed association for maximal cranial width (MCW) at 15p11.2 and (B) maximal cranial length (MCL) at 17q11.2. LocusZoom plots show the association (left y-axis; log10-transformed p-values) with facial traits. Genotyped SNPs are depicted by asterisks and imputed SNPs are depicted by circles. Shading of the points represent the linkage disequilibrium (r^2^, based on the 1000 Genomes Project Europeans) between each SNP and the top SNP, indicated by purple shading. Grey points in these plots represent the lack of LD information between the index SNP (diamond) the plotted SNP (circle or asterisk). The blue overlay shows the recombination rate (right y-axis). Positions of genes are shown below the plot.

Our meta-analysis revealed genome-wide significant associations at 15p11.2 for MCW (lead SNP: rs2924767, p = 2.11x10^-8^) and at 17q11.2 for MCL (lead SNP: rs72841279, p < 5.29x10^-9^). LocusZoom plots for these two loci are shown in Fig 1. In addition, 16 loci showed suggestive association (p < 5x10^-6^) with one of the three traits via meta-analysis (Tables 1 and S1). Moreover, another 16 cohort-specific suggestive loci were observed across the three traits. S2–S4 Tables list association results for all SNPs meeting genome-wide significant or suggestive p-value thresholds in at least one cohort or in the meta-analysis, for MCW, MCL and CI, respectively. Manhattan plots (S1 Fig) and LocusZoom plots (S2–S4 Figs) show the results for each cohort individually and for the meta-analysis.

**Table 1:** Significant and suggestive association results from meta-analysis of cranial vault traits.

Trait	Lead SNP	Chr	BP	Effect allele	P-value	Beta 3DFN	Beta OFC	Possible candidate genes	LOD score
MCW	rs2693856	2	6130176	T	1.51E-07	1.366	0.9266	*SOX11*	0.06
	rs4687677	3	52893187	T	5.66E-08	-1.401	-0.9487	-	NA
	rs35199932	3	25597498	AT	4.81E-07	0.6837	0.5630	-	0.16
	rs112065802	13	97449258	C	3.56E-07	2.254	1.8364	-	0.95
	rs2924767	15	100336990	A	**2.11E-08**	0.7996	1.2160	*MEF2A*	0.65
	rs1652002	18	21474565	G	1.47E-07	1.053	2.5971	-	0.15
	rs6115764	20	3024914	C	3.70E-07	-0.8907	-0.9724	*CPXM1*, *GNRH2*, *DDRGK1*	NA
MCL	rs5837581	2	1998982062	C	8.78E-07	-0.8376	-1.3787	-	2.04
	rs6869389	5	68216835	G	2.32E-07	0.9518	0.7050	*SLC30A5*	0.31
	rs187705391	8	144115976	T	8.68E-07	-2.332	-4.060	-	NA
	chr10:102939462	10	102939462	C	2.98E-07	-0.9144	-0.8715	*DPCD*, *POLL*	0.01
	rs142836096	12	94819905	C	8.35E-07	0.9264	2.7417	-	0.39
	rs80340157	12	94730738	C	1.10E-06	0.9085	2.7432	-	0.37
	rs8063767	16	58240735	T	5.77E-07	-2.626	-3.4821	*CNOT1*, *KATNB1*	0.03
	rs61064486	16	17530870	T	5.89E-07	-0.9679	-0.8565	-	1.81
	rs72841279	17	26176833	T	**5.29E-09**	-1.696	-1.7790	*NLK*, *TMEM97*, *TNFAIP1*, *POLDIP2*	**3.37**
CI	rs7516169	1	55903443	A	7.02E-07	0.4306	0.4043	-	0.05
	rs143374074	17	69971945	G	1.07E-06	1.226	0.4646	*SOX9*	**3.79**

LOD score from genome-wide linkage scan in mice. Bold values indicate genome-wide significant results.

Genes at a distance of ± 500 kb from each lead SNP were queried for possible roles in skull development in several databases. Corroborating evidence, such as expression in relevant tissues or putative roles in relevant human syndromes, were found for 18 of the 34 loci.

### *In silico* replication study

We tested seven SNPs in *FGFR1* identified from two prior candidate gene studies [5,6] of cranial vault shape. Both of these candidate gene studies were done in a study population of mixed ethnicity. We observed no associations between any of these previously reported *FGFR1* SNPs and any of our cranial vault traits (S5 Table). To further explore the possibility of a role for *FGFR1*, we examined all SNPs available in the gene (+/- 20kb) and still found no evidence of association (S6 Table). In addition, we tested SNPs from two GWASs involving related traits, including seven SNPs previously associated with intracranial volume [8] and two SNPs previously associated with isolated sagittal craniosynostosis [9]. We did not observe any association between our traits and these previously identified SNPs (S7 Table). Furthermore, we checked whether the significant and suggestive SNPs identified in the present study were associated with total and regional brain size using the online ENIGMA database [10]. None of our SNPs were associated with these brain morphology traits.

### Linkage analysis in mice

To follow up our GWAS findings, we used a mouse backcross between A/J (A) and C57BL/6J (B6) strains (N = 433) and performed quantitative trait loci (QTL) linkage analysis for three equivalent cranial vault measurements derived from microCT scans. All animals were genotyped at 882 informative autosomal SNPs using the Illumina medium density linkage panel. A Haley-Knott regression [11] approach was used to identify QTLs.

**Fig 2:**
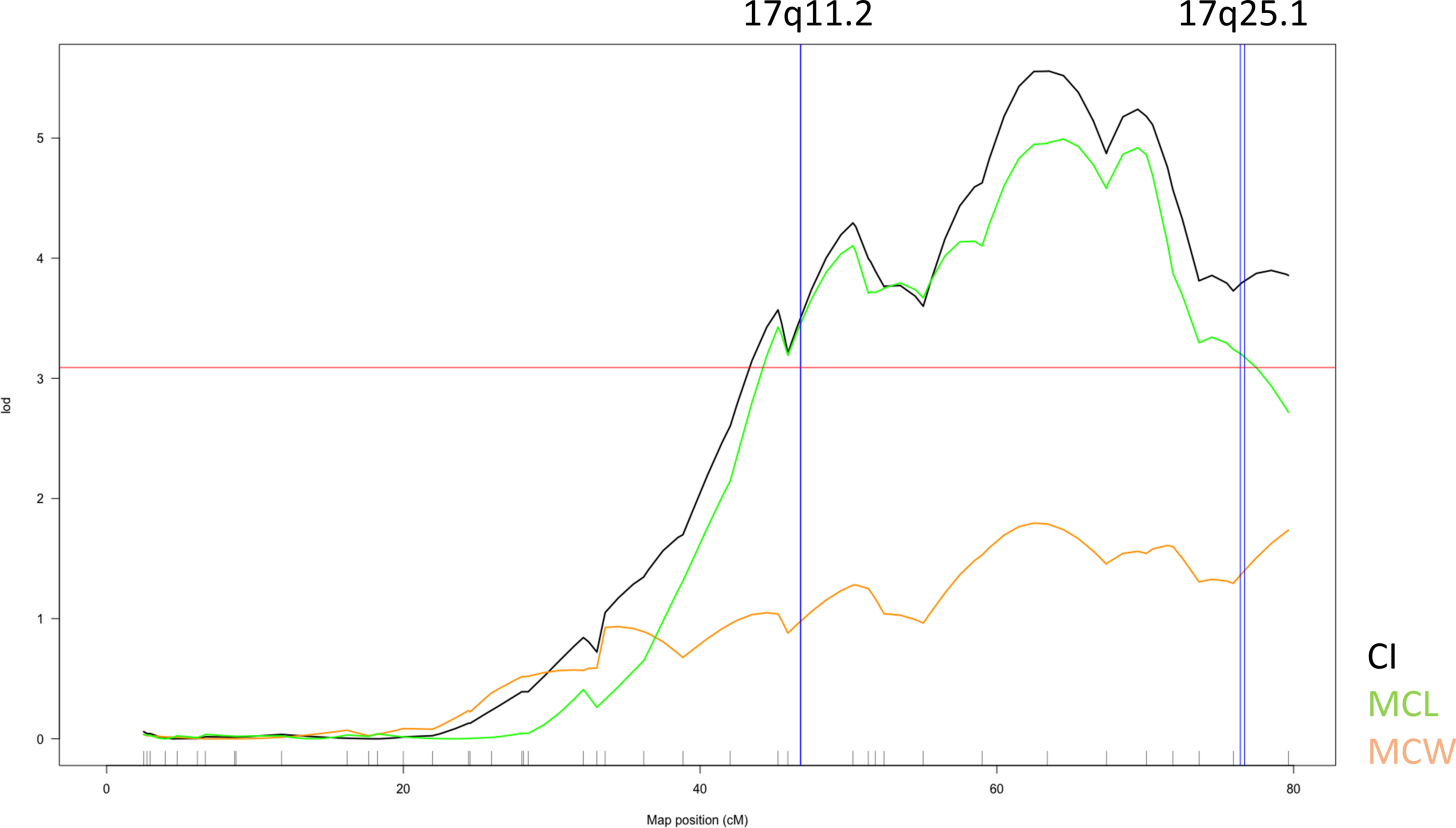
Positive linkage result in mice corresponding to two human GWAS signals. Traits are indicated by color: MCW (orange), MCL (green) and CI (black). The two corresponding human hits (17q11.2 and 17q25.1) are indicated by blue vertical lines. The horizontal line represents the permutation-based empirical threshold for genome-wide statistical significance.

Loci at 17q11.2 (c11.loc44 in mice on chr11:46765–46774) and at 17q25.1 (c11.loc74 in mice on chr11:76406–76694) aligned with a linkage peak for cranial vault size in mice (LOD scores: 3.37 and 3.79 respectively; Fig 2). In humans, the 17q11.2 signal was associated with MCL (5.29x10^-9^), while the 17q25.1 signal showed suggestive association with CI. The significant linkage peaks involved the corresponding cranial length and index traits in mice. Full results of the linkage scans are represented in S5 Fig.

## Discussion

This study represents an unbiased, genome-wide attempt to map common variants associated with normal-range cranial vault traits in humans. We observed two genome-wide significant associations. These loci were near a number of potentially relevant candidate genes. MCW was associated with locus 15p11.2 in the vicinity of *MEF2A*, while MCL was associated with locus 17q11.2 in the vicinity of *NLK*. *MEF2A* is a transcription factor involved in mediating a wide variety of basic cellular processes during development [12]. It has been previously associated with several cardiac disease phenotypes in humans [13]. *NLK* is a protein kinase that regulates a number of signaling pathways involved in cellular differentiation and proliferation, including Wnt/β-catenin and Notch [14]. Interestingly, the transcription factor *MEF2A* is a substrate for *NLK*, which negatively regulates *Wnt* signaling during the development of anterior neural structures in Xenopus Laevis [15]. Furthermore, *Nlk*-deficient mice show significant growth retardation and neurological abnormalities, indicating a disruption of normal development. The role of these genes in human cranial development is currently unknown, but they are both involved in a number of pathways critical for proper morphogenesis.

Several other genes with suggestive levels of statistical significance may play an important role in embryonic development. For instance, loci 2p15 (associated with MCW) and 17q25.1 (associated with CI) were located in the vicinity of *SOX11* and *SOX9*, respectively. *Sox11* knockout mice are known to show an abnormal cranium and cranial suture morphology, together with other craniofacial abnormalities [16]. Mutations in *SOX9* are known to cause Campomelic Dysplasia (OMIM #114290), characterized by a high forehead and a wide anterior fontanel, among other craniofacial characteristics. Furthermore, *Sox9* knockout mice are known to have a domed cranium and a decreased cranial length. Also, it is thought that *Sox9* controls patterning and closure of the frontal cranial suture in mice [17]. Both *SOX9* and *SOX11* are, thus, excellent candidate genes for normal-range variation in cranial vault shape.

Locus 11p31.1 showed suggestive associations with both MCW and CI. Several candidate genes are located near this locus. *Cnih2* and *Tsga10ip* are expressed in the mouse cranium and *CNIH2* is responsible for the normal distribution of neural crest cells in chicken [18]. *LTBP3* is responsible for the proliferation and osteogenic differentiation of human mesenchymal stem cells [19]. Furthermore, *Ltbp3* is expressed in the base of the skull of mice [20], and *Ltbp3* knock-out mice show abnormal cranium morphology, indicating that this gene is possibly involved in osteogenesis in the skull. Each of these genes may influence variation in normal cranial vault shape.

Additionally, we were able to further support one of the genome-wide significant loci and one of the suggestive loci through a genome-wide linkage analysis in mice. The genome-wide significant locus that we replicated in mice was the *nlk* locus. At the amino acid level, human *NLK* shares >99% homology with the mouse *nlk* protein. The mouse results not only support the human results, but more generally indicate that cranial vault shape in mice and humans are at least partly influenced by the same genetic loci, suggesting that mice are a relevant model system to investigate the genetics of normal-range cranial vault shape [21].

In previous genetic studies of normal-range cranial vault morphology, both Coussens and Van daal [5] and Gómez-Valdés and colleagues [6] focused on SNPs in *FGFR1*, a gene known to play a role in the etiology of craniosynostosis. When we specifically tested these SNPs none showed any evidence of replication in our dataset. Moreover, when we expanded to look at all SNPs in *FGFR1*, we also did not identify any variants associated with normal-range cranial vault shape. Both of these candidate gene studies were done in mixed ancestry cohorts. It is possible that these previously detected signals were false positives, perhaps reflecting unaccounted for stratification in the mixed-ancestry cohorts. Alternatively, normal-range cranial vault shape may be influenced by a different set of genetic factors, at least in individuals of European ancestry.

The brain is the primary driver of cranial vault expansion, and the cranial sutures function as growth sites by laying down bone at the margins [22]. Some of the nominated genes from our GWAS are involved in suture morphogenesis (e.g., *SOX9*) or in neural development (e.g., *NLK*), raising the possibility that variants in these genes may influence the shape of the cranial vault by acting on these tissues/organs. When we examined variants previously associated with either sagittal craniosynostosis or intracranial size (a proxy for brain size), we did not observe associations with any of our three traits. In fact, none of our associations involved loci harboring genes known to be responsible for numerous craniosynostosis syndromes [23]. Normal-range cranial vault shape may involve different genetic pathways or rare variants in these previously described genes which are not identifiable in GWAS. It might be valuable to explore the prevalence of SNPs identified here in individuals with craniosynostosis. The fact that we could not identify variants previously associated with intracranial volume may simply reflect the lack of power to detect these associations in our study; the size of the cohort is much smaller. However, it may also reflect fundamental differences between the traits investigated; our traits do not adequately capture overall intracranial volume but rather focus on specific dimensions and measures of shape for the external vault.

As is the case for other craniofacial traits, cranial vault shape is not solely defined by genetic influences, but also by environmental factors, which likely interact with these genetic effects. The importance of non-genetic factors influencing human vault shape has been noted since the early studies of Franz Boas at the turn of the 20^th^ century, who showed that the cephalic index of first generation children in the United States differed markedly from their recent immigrant parents. Interestingly, a reappraisal of Boas’ data has showed that he likely underestimated the genetic contribution [2]. More recently, Katz and colleagues tried to quantify the influence of diet on human skull shape [24]. They found a small directional effect of soft diets on skull shape, including the cranial vault. Unfortunately, almost nothing is known about the way genetic variants and environmental forces interact to produce normal-range craniofacial variation. This promises to be a fascinating area for future research.

## Materials and methods

### Study samples

GWAS was performed on 4419 participants from two independent cohorts, the Three Dimensional Facial Norms (3DFN) study, and the Orofacial Clefts (OFC) study. The 3DFN cohort comprised 2274 unrelated participants of self-reported European ancestry recruited at four US sites: Pittsburgh, PA; Seattle, WA; Houston, TX; and Iowa City, IA. As described previously [25], all participants were prescreened for a personal or family history of genetic syndromes with known craniofacial phenotypes and a personal history of craniofacial surgery or trauma. The OFC cohort included 2145 individuals of self-described European ancestry recruited at several US and international sites (Denmark and Hungary). This cohort comprised a combination of related family members and unrelated individuals recruited as part of a genetic study of nonsyndromic orofacial clefting [26]. Participants included in the analysis were either recruited as healthy controls (n = 796) or as the unaffected family members of cleft-affected probands (n = 1349). We applied exclusion criteria similar to the 3DFN cohort. Demographic information of both cohorts is shown in Table 2.

**Table 2:** Demographic profile of cohorts.

	3DFN	OFC
Combined N	2274	2145
Males N	886	936
Females N	1388	1209
Mean age (sd)	22.8y (9.28)	32.2y (17.59)
MCW (sd)	147.42mm (6.51)	149.32mm (7.72)
MCL (sd)	188.98mm (9.69)	187.53mm (9.63)
CI (sd)	78.08 (3.57)	79.75 (4.38)

MCW = Maximum cranial width; MCL = Maximum Cranial Width; CI = Cephalic index (MCW/MCL*100)

### Ethics statement

Institutional Review Board (IRB) approval was obtained at each recruitment center and all subjects gave written informed consent prior to participation (University of Pittsburgh IRB #PRO09060553 and IRB0405013; Seattle Children’s IRB #12107; University of Iowa Human Subjects Office/IRB #200912764 and #200710721; UT Health Committee for the Protection of Human Subjects #HSC-DB-09-0508 and #HSC-MS-03-090; Regional Committee on Biomedical Research for Southern Denmark: #S-20080105, Colorado Multiple Institutional Review Board #10–0055, Washington University in St. Luis HRPO #03–0871). This study was carried out in strict accordance with the recommendations in the Guide for the Care and Use of Laboratory Animals of the National Institutes of Health. The protocol was approved by the Institutional Animal Care and Use Committee of the University of Washington (UW—2688–07). All animals were euthanized by CO2 asphyxiation followed by decapitation.

### Phenotyping

Using spreading calipers (GPM, Switzerland), trained personnel at each recruitment site collected measures of maximum cranial width (MCW; measured as the linear distance between the left and right euryon landmarks) and maximum cranial length (MCL; measured as the linear distance between the glabella and opisthocranion landmarks) according to standard established anthropometric protocols (Fig 1) [27]. To ensure consistent data collection, all personal were calibrated against an expert anthropometrist (SMW) during formal training sessions. Test-retest error associated with these measurements was low (intraclass correlation coefficients >0.98). The cephalic index (CI), a standard measure of cranial vault shape, was calculated for each participant by dividing their maximum cranial width by their maximum cranial length and multiplying by 100 to obtain a proportion. Outliers (N = 19 for MCW, N = 16 for MCL and N = 15 for CI) with cranial vault measurements more than three SD from the mean for their age and sex group were excluded from statistical analysis.

### Genotyping, quality control, population structure and imputation

For the 3DFN cohort, DNA was extracted from saliva samples and genotyped along with 72 HapMap control samples for 964193 SNPs on the Illumina (San Diego, CA) HumanOmniExpress+Exome v1.2 array plus 4322 SNPs of custom content by the Center for Inherited Disease Research (CIDR). For the OFC cohort, DNA was extracted from saliva, blood, or other biological samples and genotyped along with the same 72 HapMap controls for 551787 SNPs on an Illumina HumanCore+Exome array plus 15890 SNPs of custom content, also by CIDR. Genetic data cleaning and quality control analyses were performed as described in detail, previously [28,29]. In brief, samples were interrogated for genetic sex, chromosomal aberrations, relatedness, genotype call rate, and batch effects. SNPs were interrogated for call rate, discordance among 70 (3DFN) or 72 (OFC) duplicate samples, Mendelian errors among HapMap controls (parent-offspring trios), deviations from Hardy-Weinberg equilibrium, and sex differences in allele frequencies and heterozygosity. Filters applied for genotyped SNPs are described in S8 Table.

To assess population structure, we performed principal component analysis (PCA) within each cohort using subsets of uncorrelated SNPs. Based on the scatterplots of the principal components (PCs) and scree plots of the eigenvalues (S6 Fig), we determined that population structure was captured in four PCs of ancestry for the 3DFN cohort and 18 PCs of ancestry in the OFC cohort (this information was not included in the GWS since a mixed model was used).

A total of 9482681 and 9211574 imputed SNPs were tested for the 3DFN and OFC cohorts respectively. Imputation of unobserved variants was performed using haplotypes from the 1000 Genomes Project Phase 3 as the reference. Imputation was performed using IMPUTE2 [30]. We used an info score of >0.5 at the SNP level and a genotype probability of >0.9 at the SNP-per-participant level as filters for imputed SNPs (S8 Table). Masked variant analysis, in which genotyped SNPs were imputed in order to assess imputation quality, indicated high accuracy of imputation.

### Association analyses

Genetic association with MCW, MCL and CI was tested in 3DFN and OFC cohorts separately for 968515 (for the 3DFN cohort) or 567677 (for the OFC cohort) genotyped and imputed SNPs with MAF > 1%. For 3DFN, GWAS was performed using linear regression while adjusting for sex, age, height, weight and four PCs of ancestry as implemented in PLINK [31]. For OFC, which included relatives, GWAS was performed using a mixed-models approach as implemented in EMMAX, which explicitly models the variance due to the kinship (comprising both the biological relatedness and population structure) in the sample. Sex, age, height, weight, and cleft-family status were included as covariates. PCs of ancestry were not included for OFC because variation due to population structure was already explicitly modeled by the kinship matrix. We also investigated additional adjustments such as age-squared to deal with potential non-linear growth effects. In our covariate modeling (using Aikake Information Criterion tests and inspecting residual plots), we found no evidence of improved fit with such non-linear covariates. In both cohorts, GWAS was performed under the additive genetic model. For the analysis of the X-chromosome, genotypes were coded 0, 1, and 2 as per the additive genetic model for females, and coded 0, 2 for males in order to maintain the same scale between sexes. GWAS results for each study were combined via inverse variance-weighted meta-analysis using Stouffer’s p-value method [7]. The conventional threshold of 5x10^-8^ was set for genome-wide statistical significance. QQ plots and genomic inflation scores are represented in S8 Fig.

### Functional annotation

Lead SNPs at associated loci were queried using HaploReg [32] to extract evidence of functional variation (promoter and enhancer histone marks, DNAse hypersensitivity, eQTL information) for all SNPs in LD (r^2^ > 0.8) with the lead SNPs. Genes of interest were defined based on physical proximity of 500 kb to the lead SNP at each locus. These genes were queried in the following online databases: The Mouse Genome Informatics (MGI) database [33], which was used to annotate expression in relevant tissues and phenotypic consequences, the VISTA enhancer database [34], which was used to annotate active enhancer elements in relevant tissues, and OMIM and PubMed, which were used to annotate human phenotypic information.

### *In silico* replication of previous genetic associations

Seven SNPs in *FGFR1* from two prior candidate gene studies [5,6] which showed nominal evidence of association with either MCL or CI, were explicitly tested for replication in our cohorts. In addition, SNPs from two GWASs involving related cranial traits were tested for association with our three cranial vault traits, including seven SNPs previously associated with intracranial volume [18] and two SNPs previously associated with isolated sagittal craniosynostosis [9]. Furthermore, significant and suggestive SNPs identified in this study were checked for a possible association with brain size or brain structure, using the online ENIGMA database [10].

### Testing associated variants in mice

To follow up our GWAS findings, we used a mouse backcross between A/J (A) and C57BL/6J (B6) strains and performed quantitative trait loci (QTL) linkage analysis for three cranial vault measurements. To make our phenotypes consistent, we selected landmarks that closely approximate our measurements in humans, indicated in S7 Fig. For cranial vault length we used 3D distance from the midpoint of the frontonasal suture to the intersection of the interparietal bones with squamous portion of occipital bone in the midline, as the mouse study lacked opisthion as a landmark. Vault width was measured as the 3D distance between the left and right points corresponding to the intersection of most anterolateral aspect of parietal bone with the temporal bone. The equivalent of cephalic index was calculated as the ratio of skull length to skull width. To these variables, we fitted a linear regression to account for the effects of skull size and the direction of cross, and used residuals from this regression as our phenotypes to be mapped. All animals (N = 433) were genotyped at 882 informative autosomal SNPs using the Illumina medium density linkage panel. Additional details regarding the genotyping and phenotyping are available [35].

We used Haley-Knott regression [11] as implemented in R/qtl [36] to identify QTLs for our phenotypes. The genome-wide significance threshold was determined using a permutation test based on 10000 replicates [37]. We intersected the mouse QTL results with the human findings using the following procedure: human hits were first converted to mouse genomic coordinates and were then converted to sex-averaged genetic distances using Cox mouse map [38]. To determine the genomic interval of interest, we used arbitrarily defined 1 cM intervals centered on the average genetic distance of the human hits. If this interval contained a mouse QTL for that particular phenotype, we considered this as corroborating evidence in support of the associated locus in human. This procedure is more conservative than using the traditional 1-LOD support interval around the peak, which can be tens of cM in length.

### Data access

The genotype data for both human cohorts are available through dbGaP [https://www.ncbi.nlm.nih.gov/gap; dbGaP Study Accession: phs000094.v1.p1 (OFC) and phs000949.v1.p1 (3DFN)]. Cranial measurements for the OFC cohort are also available through the same dbGaP accession. Cranial measurements for the 3D Facial Norms cohort are available through the FaceBase Consortium (Accession #: FB00000491.01; https://www.facebase.org/data/record/#1/isa:dataset/accession=FB00000491.01). A full description of the 3D Facial Norms dataset is available at https://www.facebase.org/facial_norms/. Although there are no costs associated with access to FaceBase datasets, users must formally apply for access to human datasets through the FaceBase Consortium (the application process is described here: https://www.facebase.org/methods/policies/). Full summary statistics for all SNPs are available upon request.

## Supporting information

S1 Table P-values of all significant and suggestive SNPs in all three traits. (XLSX)

S2 Table All genome-wide significant and suggestive SNPs in the 3DFN cohort, OFC cohort and meta-analysis for MCW. (XLSX)

S3 Table All genome-wide significant and suggestive SNPs in the 3DFN cohort, OFC cohort and meta-analysis for MCL. (XLSX)

S4 Table All genome-wide significant and suggestive SNPs in the 3DFN cohort, OFC cohort and meta-analysis for CI. (XLSX)

S5 Table Association results of *FGFR1* SNPs identified from two published cranial vault candidate gene studies. (XLSX)

S6 Table Association results of all available *FGFR1* SNPs (+/- 20kb). (XLSX)

S7 Table Association results of SNPs identified from previously published GWASs of intracranial volume and sagittal craniosynostosis. (XLSX)

S8 Table Overview of the quality control, methods and population structure for genotyped and imputed SNPs in each of the main cohorts. (DOCX)

S1 Fig Manhattan plots for all genome-wide significant and suggestive SNPs in the 3DFN cohort, OFC cohort and meta-analysis for MCW, MCL and CI. The horizontal line represents the conventional threshold for genome-wide statistical significance: p ≤ 5x10^-8^.
(PDF)

S2 Fig LocusZoom plots for all genome-wide significant and suggestive SNPs in the 3DFN cohort for MCW, MCL and CI. LocusZoom plots show the association (left y-axis; log10-transformed p-values) with facial traits. Genotyped SNPs are depicted by asterisks and imputed SNPs are depicted by circles. Shading of the points represent the linkage disequilibrium (r2, based on the 1000 Genomes Project Europeans) between each SNP and the top SNP, indicated by purple shading. Grey points in these plots represent the lack of LD information between the index SNP (diamond) the plotted SNP (circle or asterisk). The blue overlay shows the recombination rate (right y-axis). Positions of genes are shown below the plot.
(PDF)

S3 Fig LocusZoom plots for all genome-wide significant and suggestive SNPs in the OFC cohort for MCW, MCL and CI. LocusZoom plots show the association (left y-axis; log10-transformed p-values) with facial traits. Genotyped SNPs are depicted by asterisks and imputed SNPs are depicted by circles. Shading of the points represent the linkage disequilibrium (r2, based on the 1000 Genomes Project Europeans) between each SNP and the top SNP, indicated by purple shading. Grey points in these plots represent the lack of LD information between the index SNP (diamond) the plotted SNP (circle or asterisk). The blue overlay shows the recombination rate (right y-axis). Positions of genes are shown below the plot.
(PDF)

S4 Fig LocusZoom plots for all genome-wide significant and suggestive SNPs in the meta-analysis for MCW, MCL and CI. LocusZoom plots show the association (left y-axis; log10-transformed p-values) with facial traits. Genotyped SNPs are depicted by asterisks and imputed SNPs are depicted by circles. Shading of the points represent the linkage disequilibrium (r2, based on the 1000 Genomes Project Europeans) between each SNP and the top SNP, indicated by purple shading. Grey points in these plots represent the lack of LD information between the index SNP (diamond) the plotted SNP (circle or asterisk). The blue overlay shows the recombination rate (right y-axis). Positions of genes are shown below the plot.
(PDF)

S5 Fig Genome-wide linkage scan plots for cranial vault traits in mice. The three traits are indicated by color: MCW = Orange, MCL = Green, CI = Black. The horizontal line represents the permutation-based empirical threshold for genome-wide statistical significance.
(PDF)

S6 Fig Ancestry PC plots and scree plots for OFC and 3DFN cohort. (PDF)

S7 Fig Overview of landmarks used to measure cranial vault dimensions from mouse skull microCT scans. (TIFF)

S8 Fig QQ Plots and corresponding genomic inflation factors for all association studies. (PDF)
