# Supplementary material for: Mapping genetic variants for cranial vault shape in humans
Source: PLoS One. 2018 Apr 26;13(4):e0196148. doi: 10.1371/journal.pone.0196148 (PMC5919379; doi:10.1371/journal.pone.0196148)
Supplement: S8 Table — (DOCX) [file pone.0196148.s008.docx]

Supplementary Table 8a. Number of genotyped SNPs omitted and retained for each quality filter in both 3DFN and OFC data

|  |  | **3DFN** | | **OFC** | |
| --- | --- | --- | --- | --- | --- |
| **Step** | **Filter** | **SNPs omitted** | **SNPs cumulatively retained** | **SNPs**  **omitted** | **SNPs**  **cumulative retained** |
| 1 | None (all SNPs) |  | 968,515 |  | 557,677 |
| 2 | CIDR technical filters | 8,470 | 960,045 | 9,625 | 548,052 |
| 3 | Missing call rate ≥ 2% | 9,675 | 950,370 | 5,891 | 542,161 |
| 4 | >1 discordant calls in 69 duplicates ^a c^  >2 discordant calls in 264 study duplicates ^b^ | 26 | 950,344 | 42 | 542,119 |
| 5 | >1 Mendelian error across 8 HapMap trios ^a^  >20 Mendelian errors in 5,288 parent-offspring trios or dyads ^b^ | 122 | 950,222 | 2,410 | 539,709 |
| 6 | HWE p-value < 10^-4^ | 2,038 | 948,184 | 194 | 539,515 |
| 7 | Allele frequency difference ≥ 0.2 between sexes ^d^ | 274 | 947,910 | 42 | 539,473 |
| 8 | Heterozygosity difference > 0.3 between sexes ^d^ | 41 | 947,869 | 0 | 539,473 |
| 9 | Positional duplicates | 19,597 | 928,272 | 7,278 | 532,195 |
| 10 | Monomorphic (MAF = 0) | 108,485 | 819,787 | 76,746 | 455,449 |
| 11 | MAF < 0.01 | 162,618 | 659,955 | 161,816 | 293,633 |

^a^ 3DFN filter

^b^ OFC filter

^c^ one duplicate was removed from QC filters due to a chromosomal anomaly

^d^ filter applied to SNPs on autosomes and XY pseudo-autosomal region on homogeneous genetic ancestry sample

Supplementary Table 8b. Number of imputed SNPs omitted and retained for each quality filter in both 3DFN and OFC data

|  |  | **3DFN** | | **OFC** | |
| --- | --- | --- | --- | --- | --- |
| **Step** | **Filter** | **SNPs omitted** | **SNPs cumulatively retained** | **SNPs**  **omitted** | **SNPs**  **cumulative retained** |
| 1 | None (all SNPs) |  | 34,985,077 |  | 32,630,291 |
| 2 | Info score < 0.5 | 14,834,852 | 20,150,225 | 3,654,625 | 28,975,666 |
| 3 | MAF < 0.01 | 10,667,544 | 9,482,681 | 19,764,092 | 9,211,574 |
