# Supplementary figures and images for: Mapping genetic variants for cranial vault shape in humans
Source: PLoS One. 2018 Apr 26;13(4):e0196148. doi: 10.1371/journal.pone.0196148 (PMC5919379; doi:10.1371/journal.pone.0196148)

A Figure S2

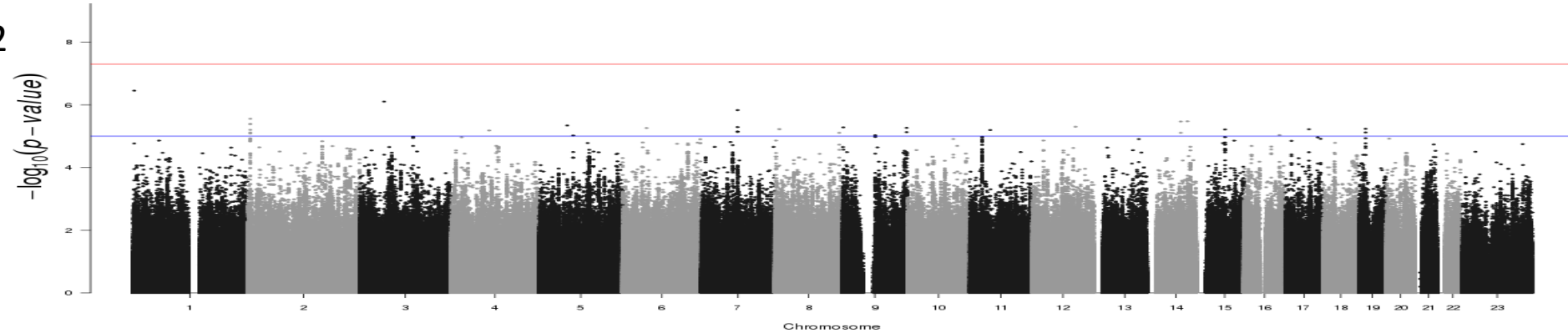

3DFN

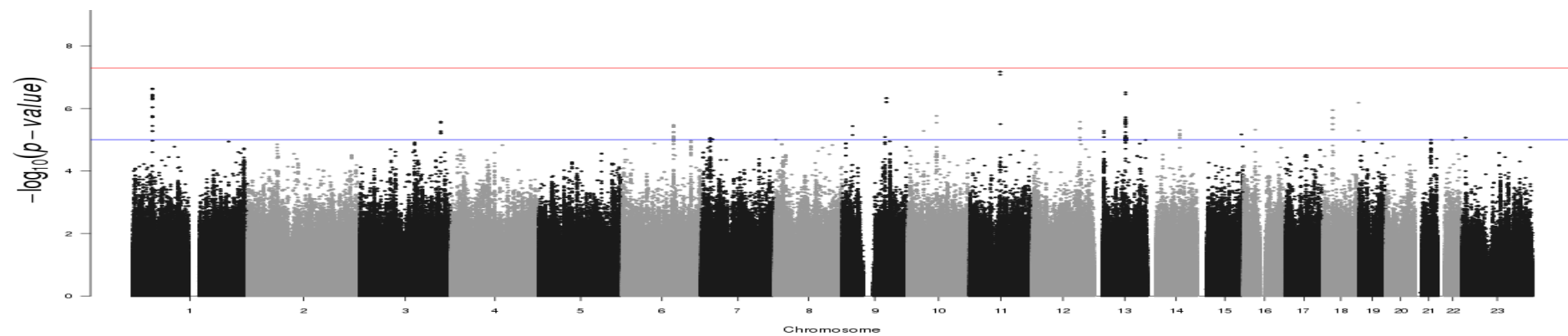

OFC

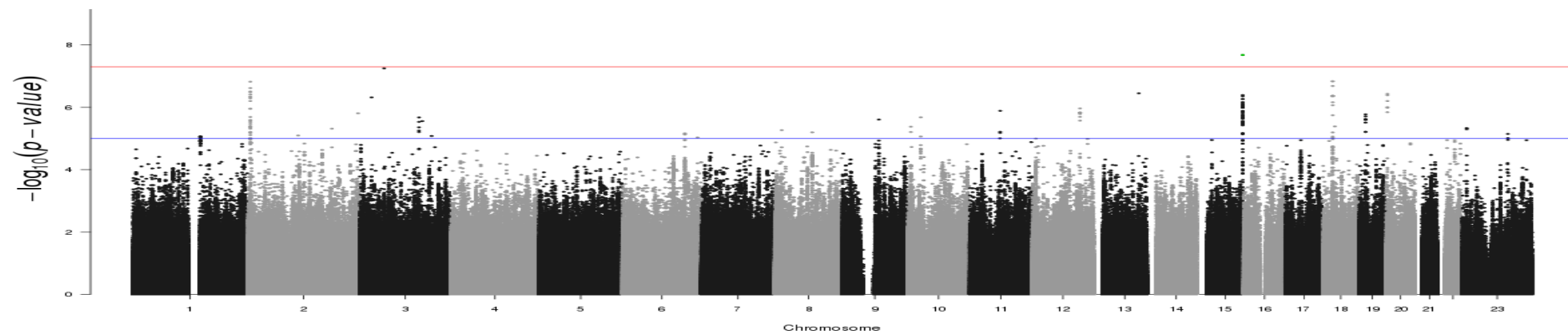

Meta

B

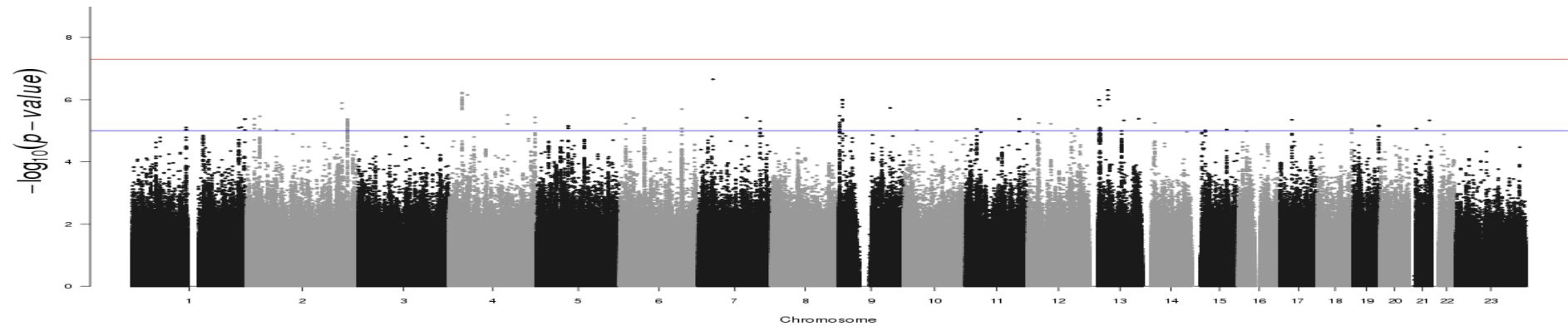

3DFN

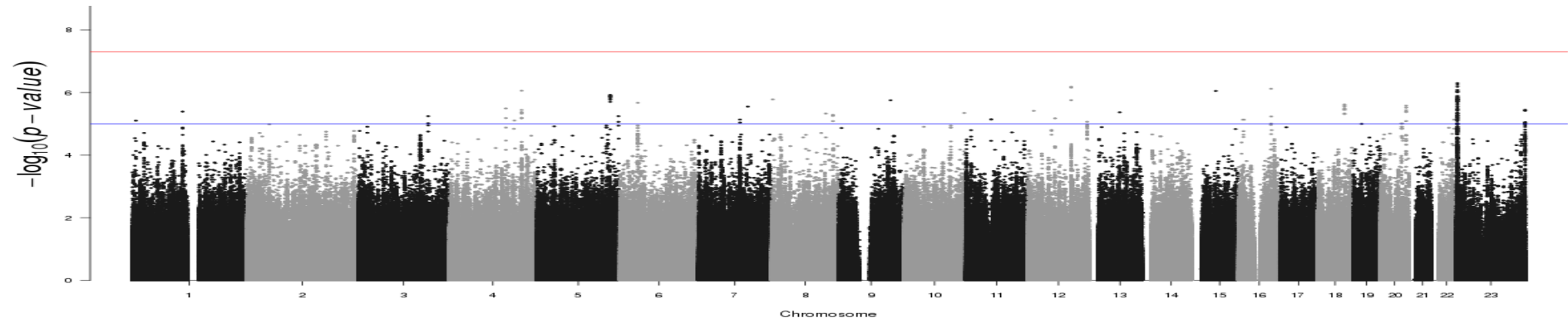

OFC

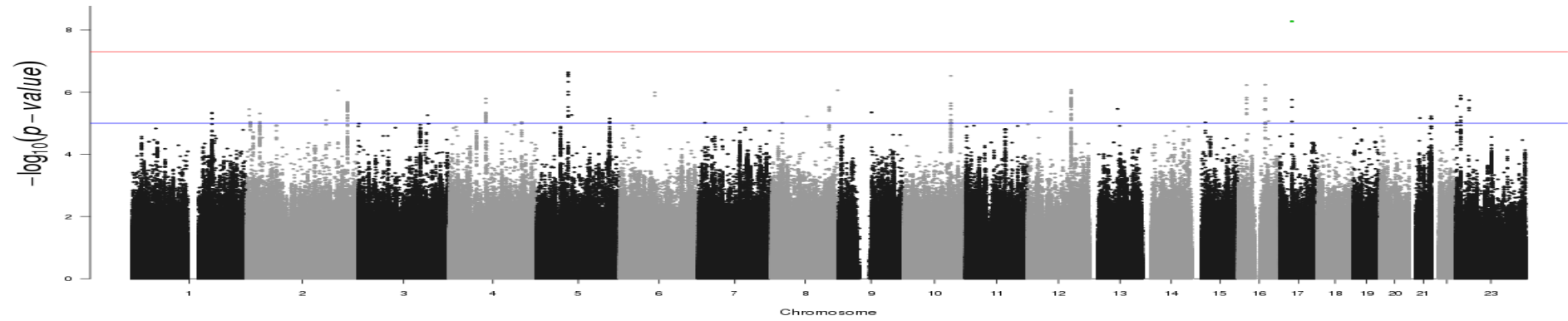

Meta

C

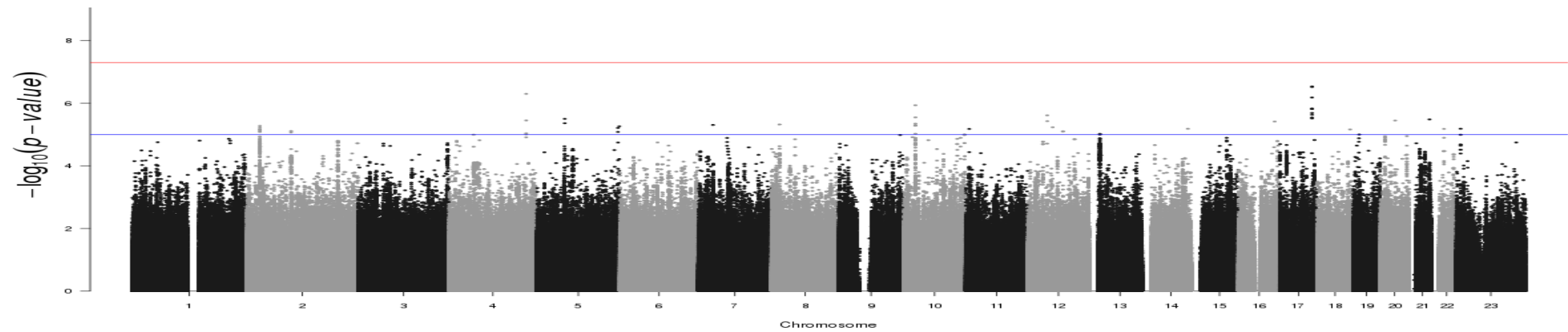

3DFN

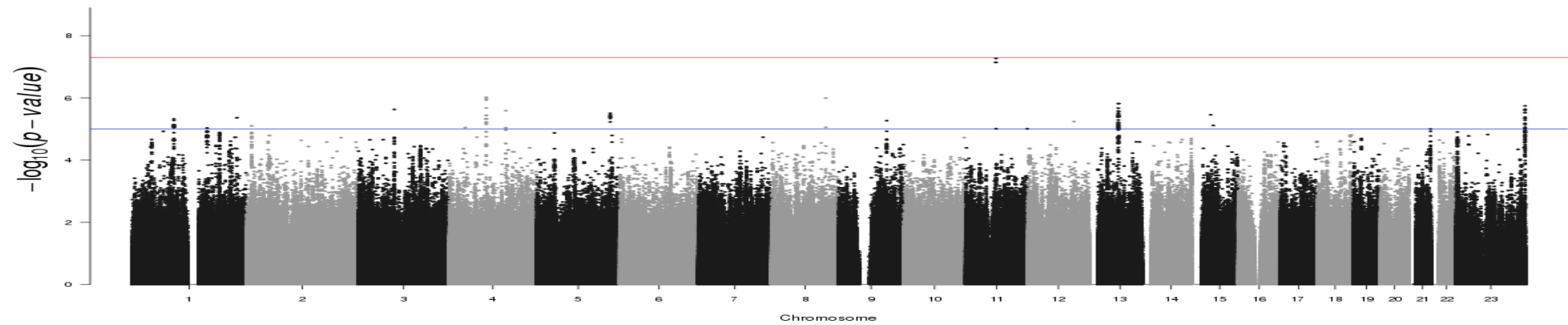

OFC

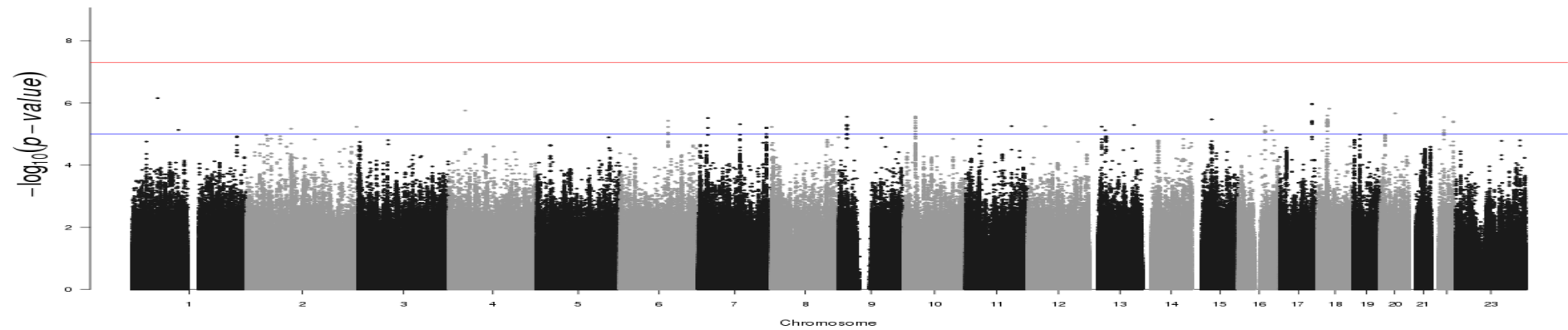

Meta

Supplement: S1 Fig — The horizontal line represents the conventional threshold for genome-wide statistical significance: p ≤ 5x10^-8^. (PDF) [file pone.0196148.s009.pdf]

Figure S3

FV.MCW

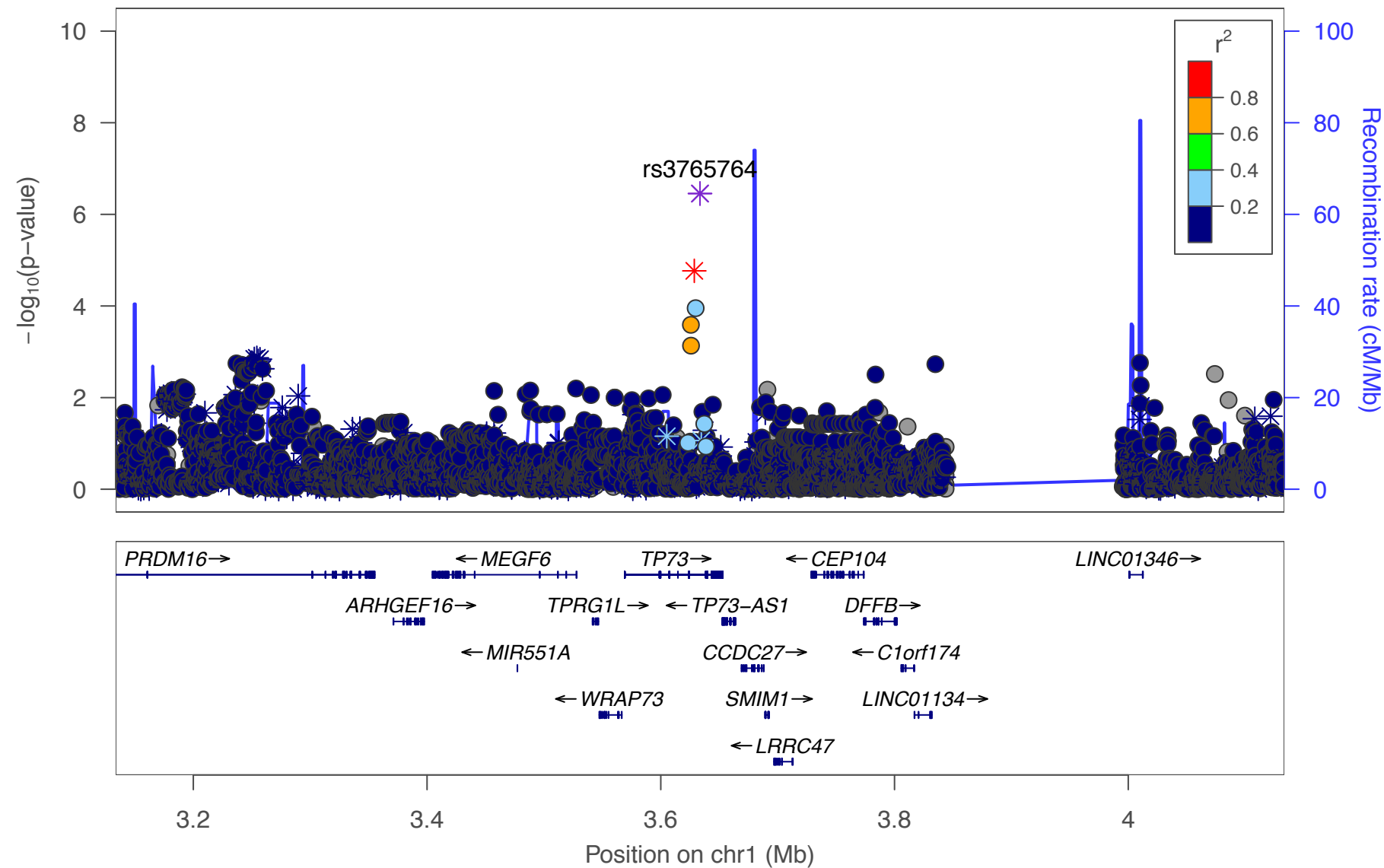

# FV.MCW

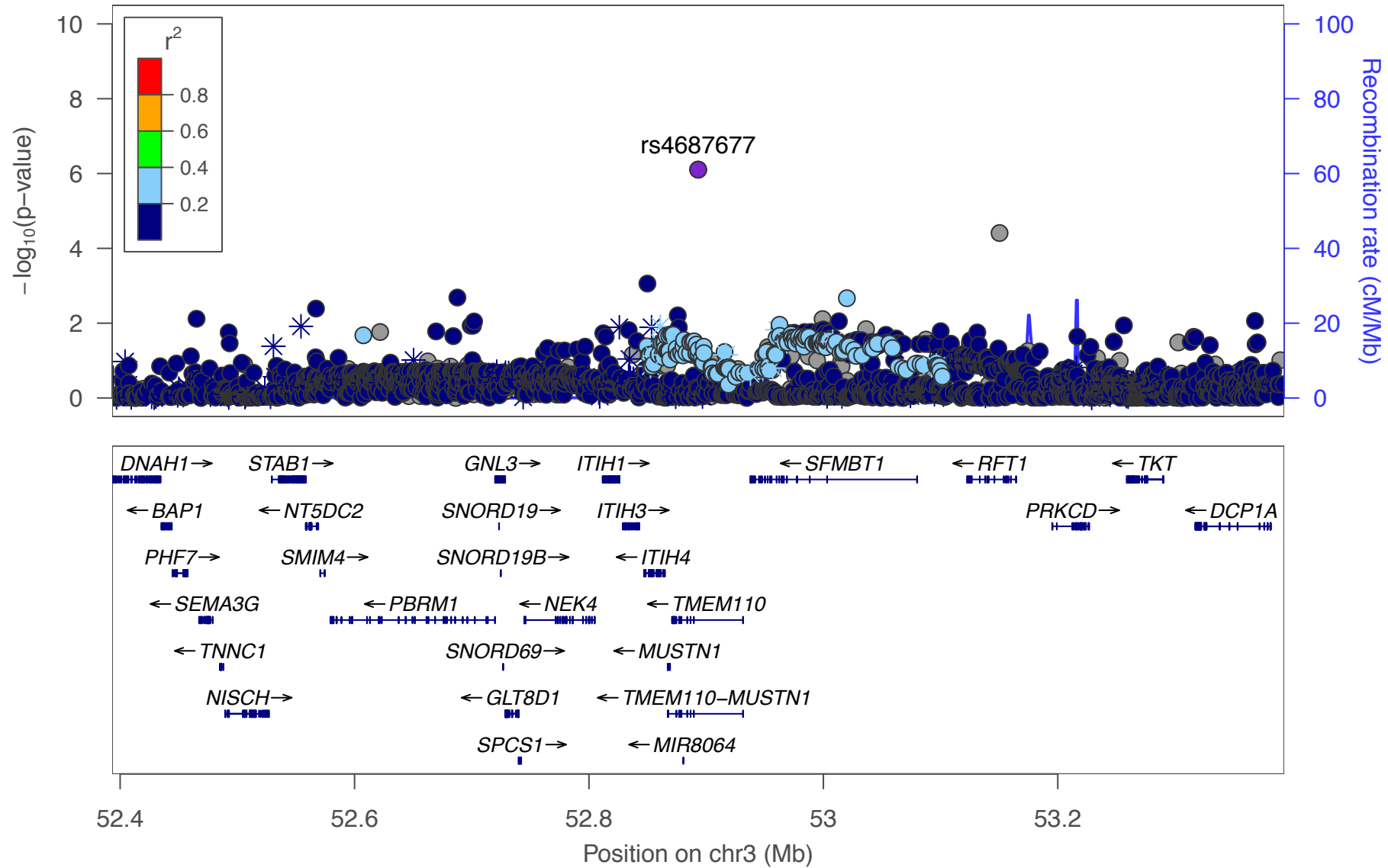

# FV.MCL

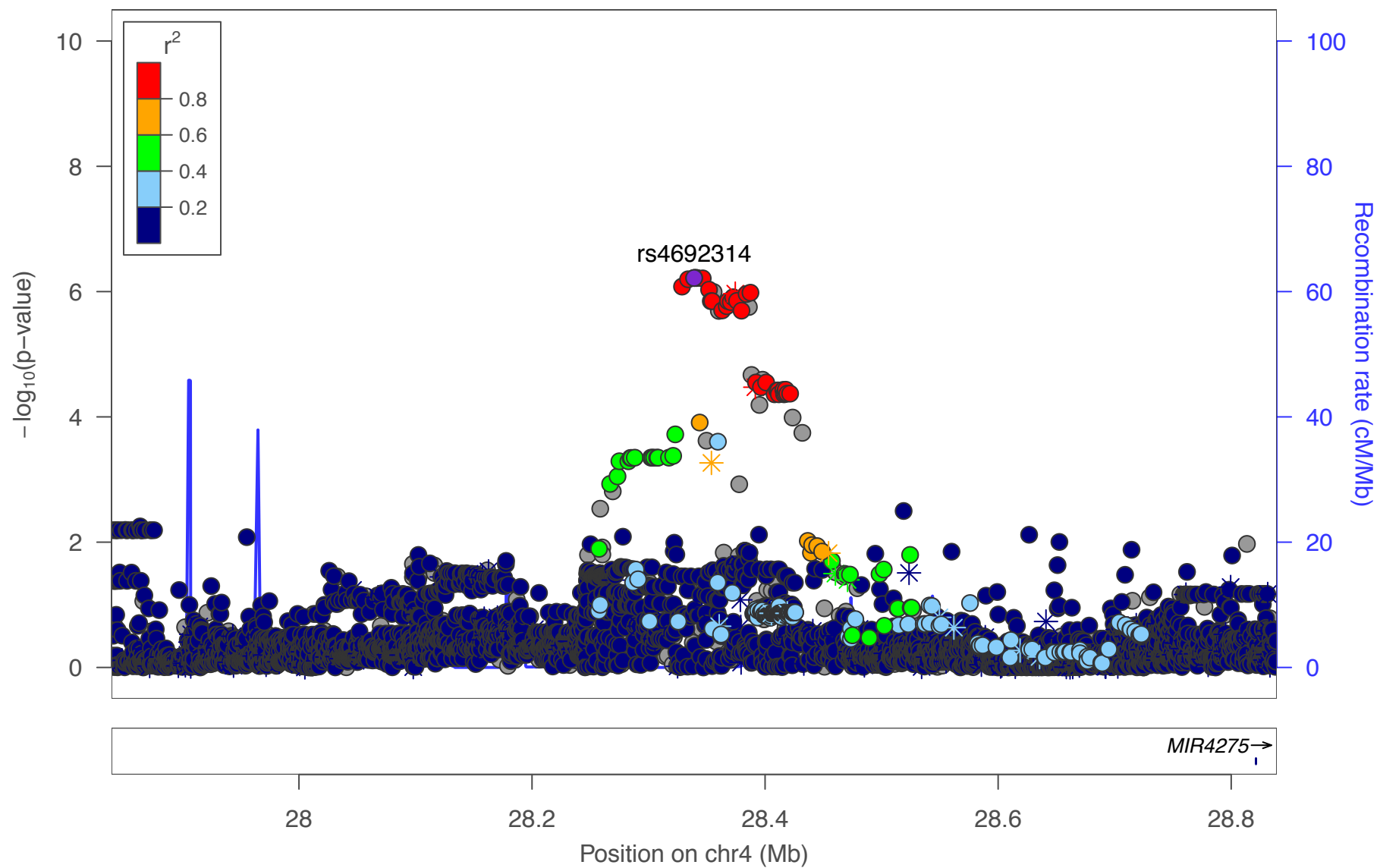

# FV.MCL

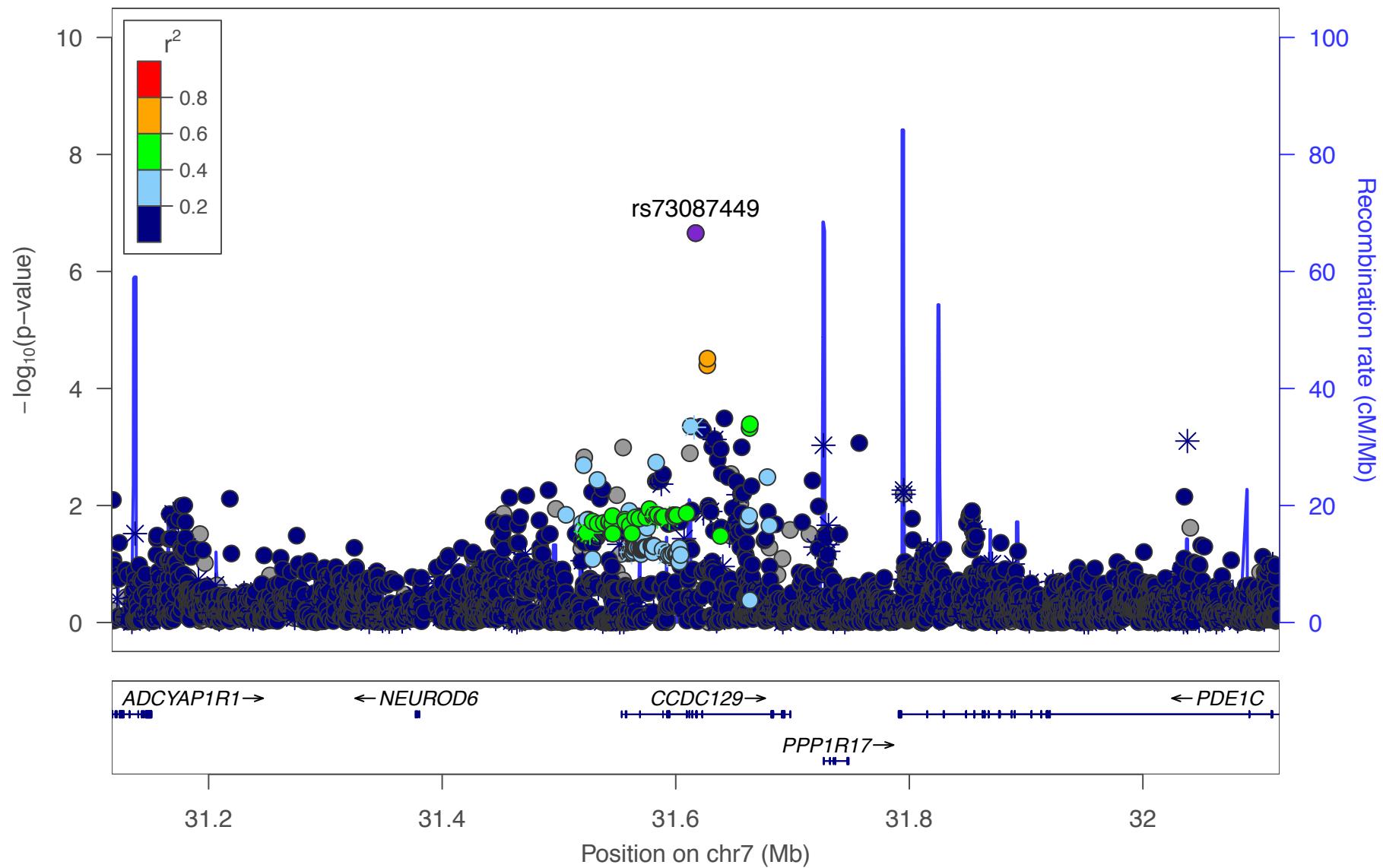

# FV.MCL

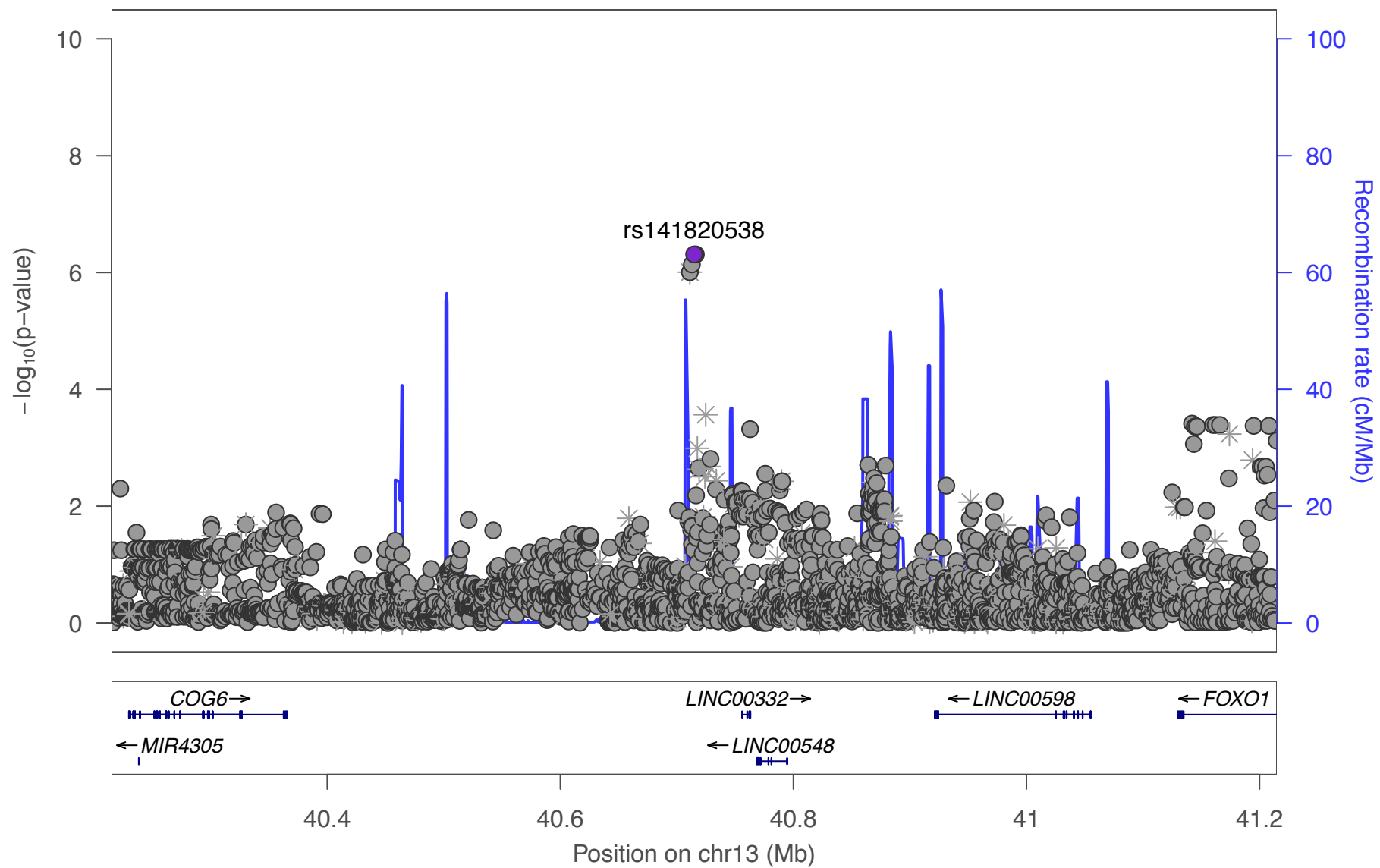

# FV.CI

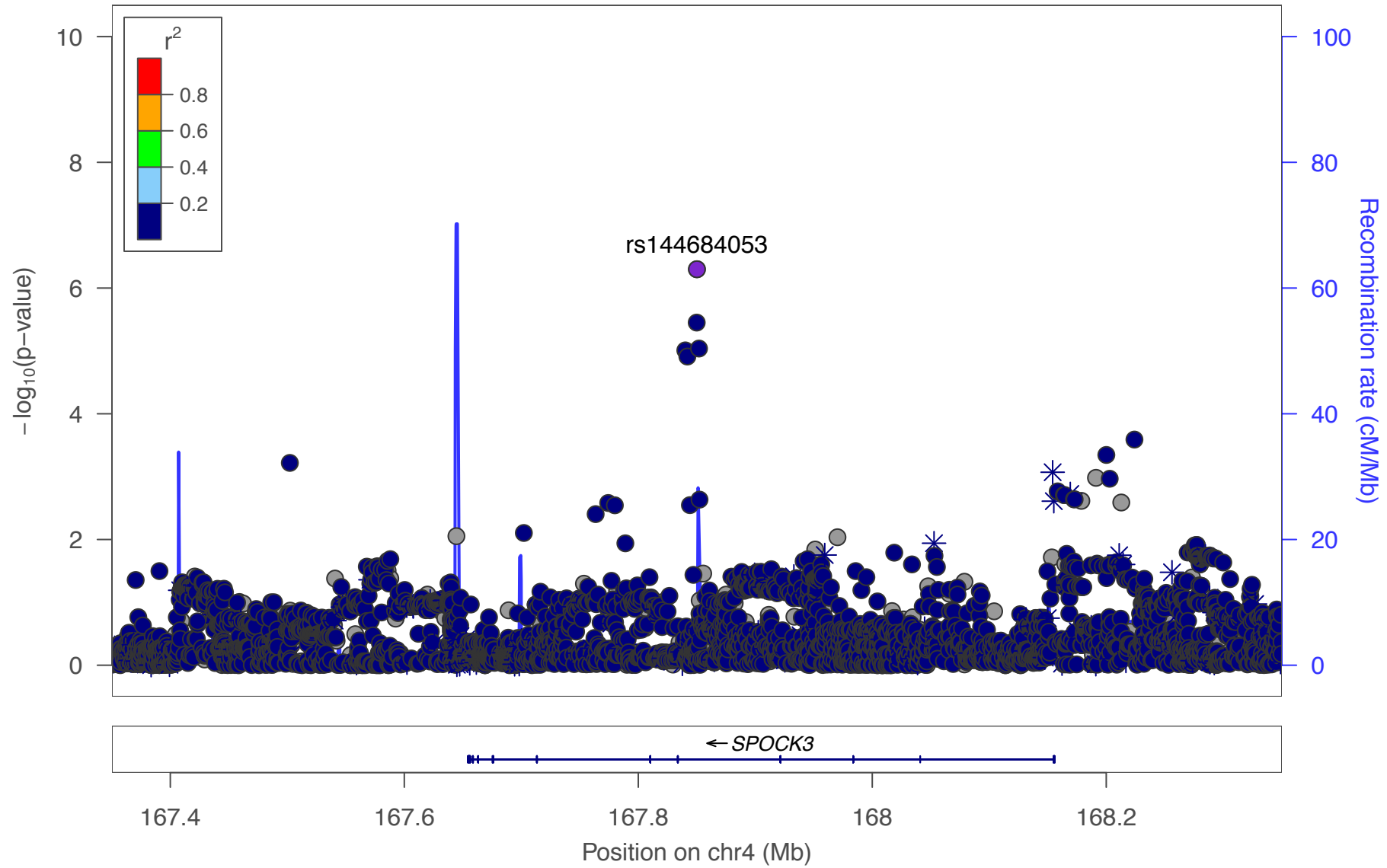

# FV.CI

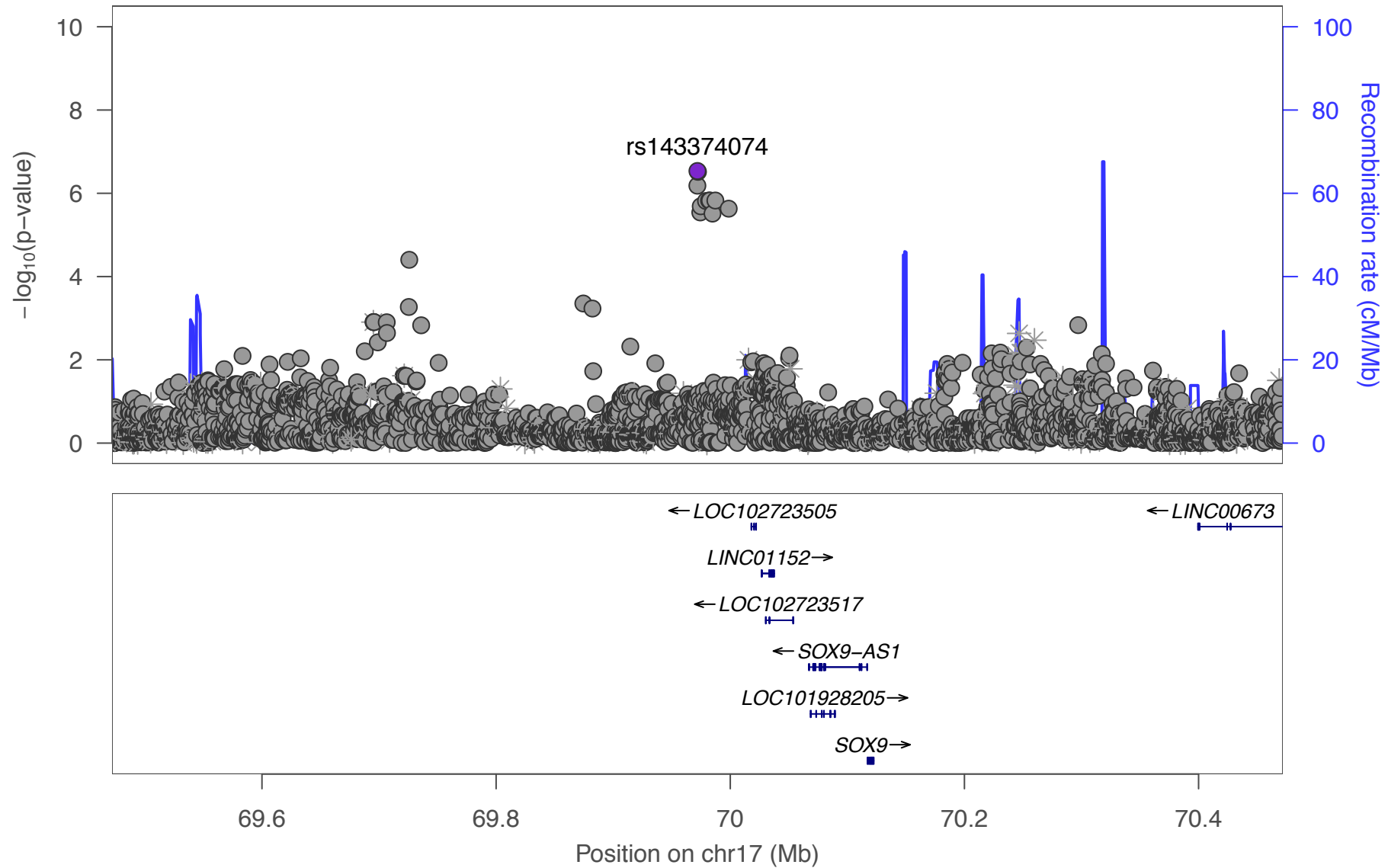

Supplement: S2 Fig — LocusZoom plots show the association (left y-axis; log10-transformed p-values) with facial traits. Genotyped SNPs are depicted by asterisks and imputed SNPs are depicted by circles. Shading of the points represent the linkage disequilibrium (r2, based on the 1000 Genomes Project Europeans) between each SNP and the top SNP, indicated by purple shading. Grey points in these plots represent the lack of LD information between the index SNP (diamond) the plotted SNP (circle or asterisk). The blue overlay shows the recombination rate (right y-axis). Positions of genes are shown below the plot. (PDF) [file pone.0196148.s010.pdf]

Figure S4

OFC.MCW

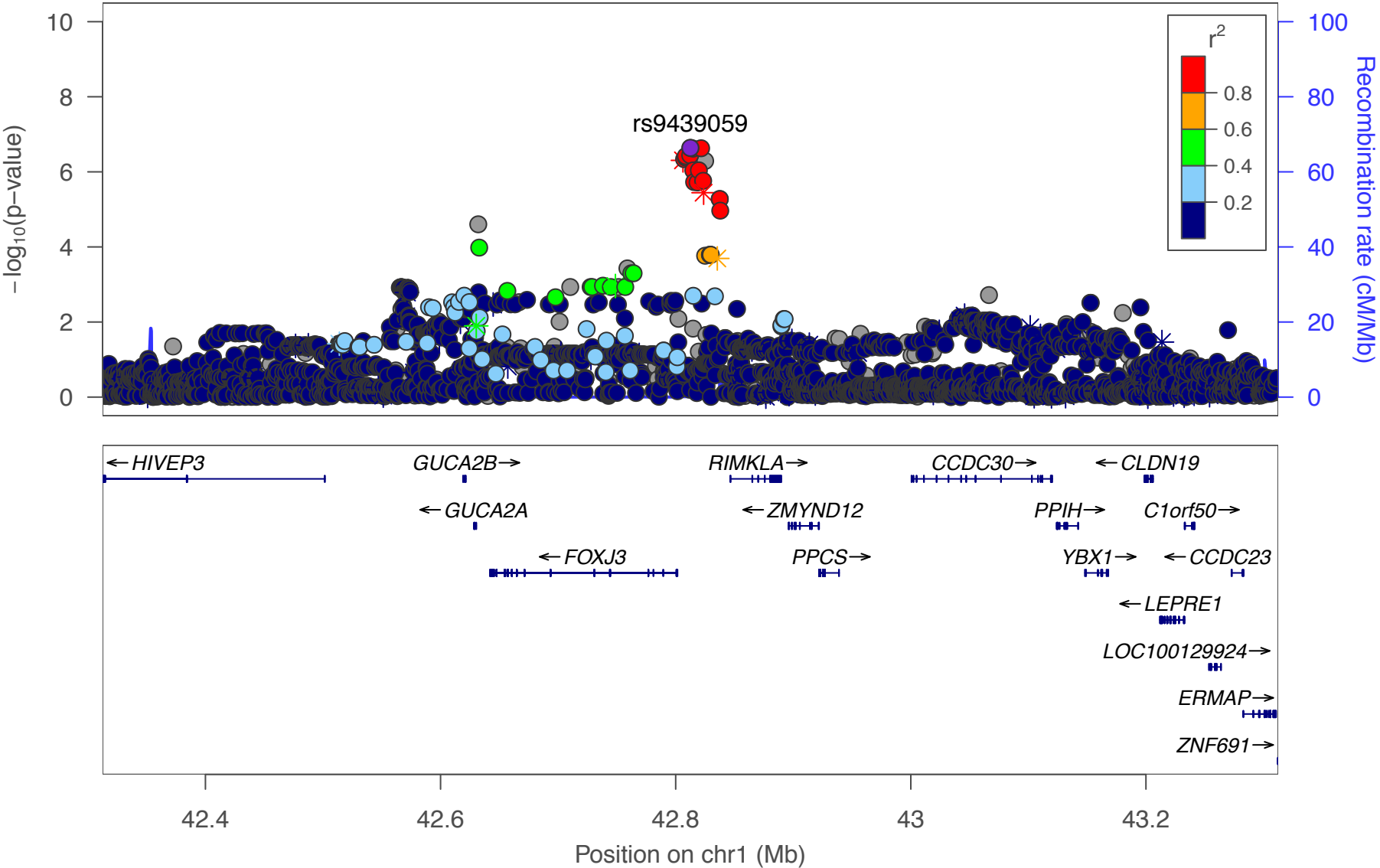

# OFC.MCW

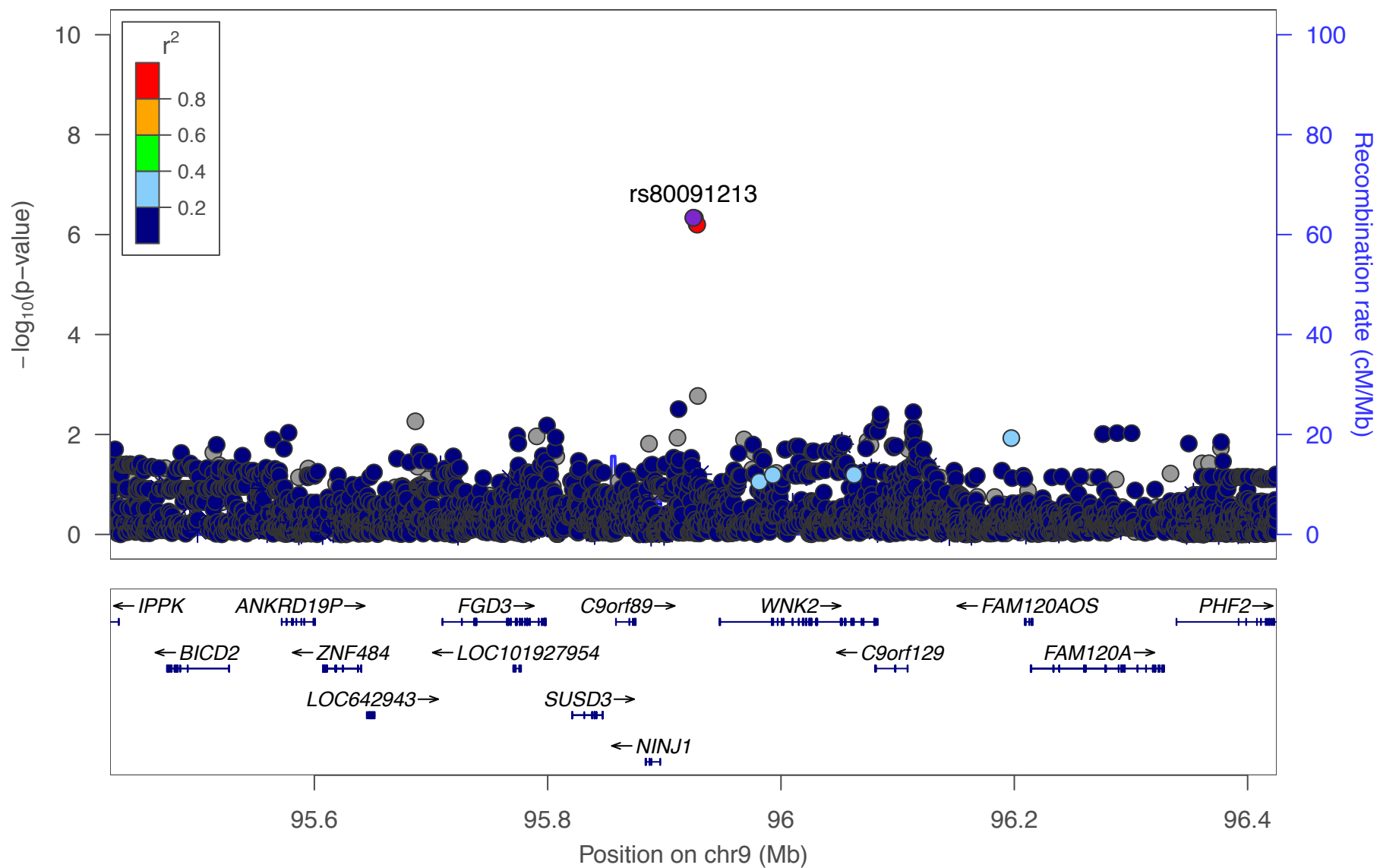

# OFC.MCW

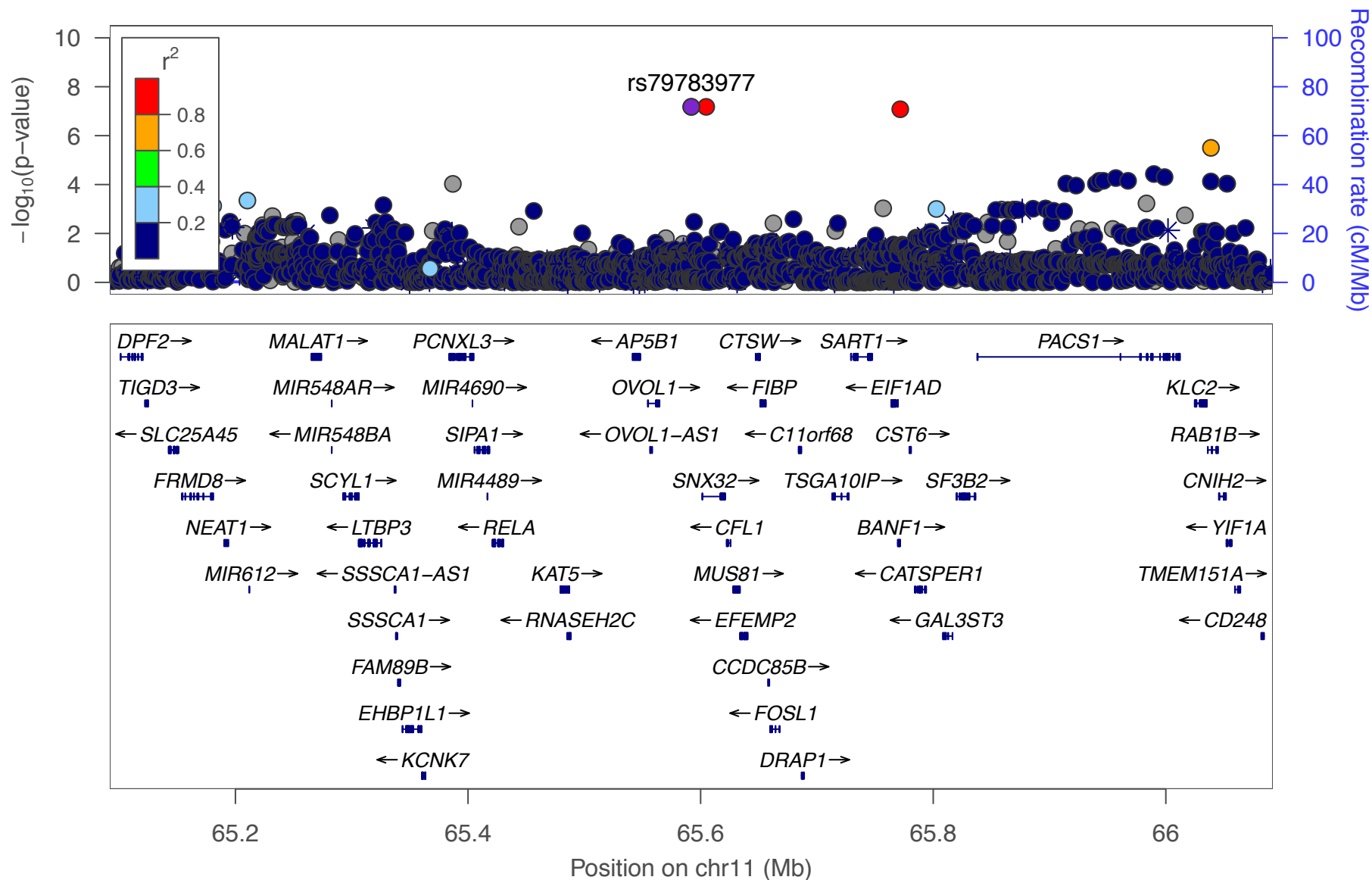

# OFC.MCW

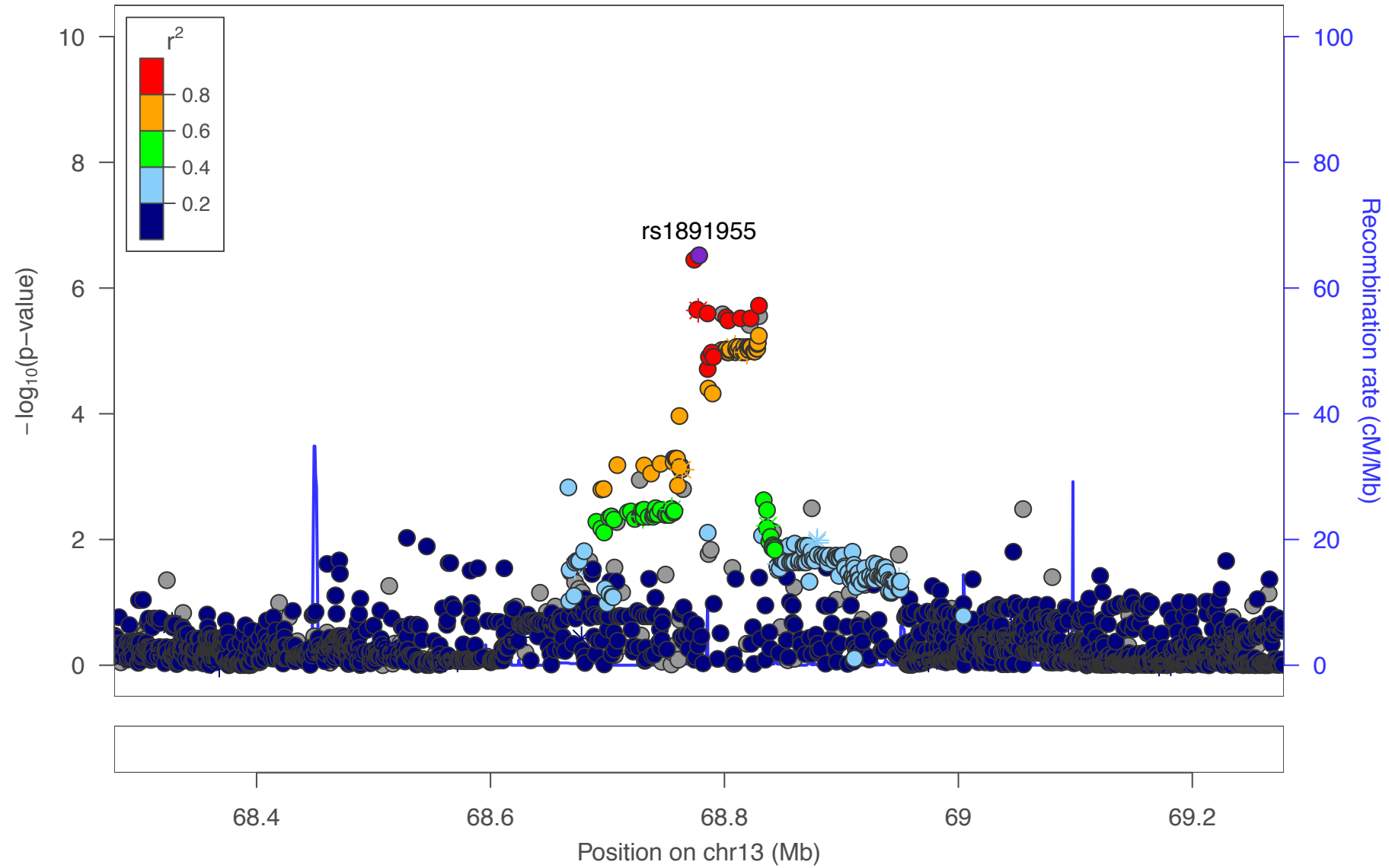

# OFC.MCW

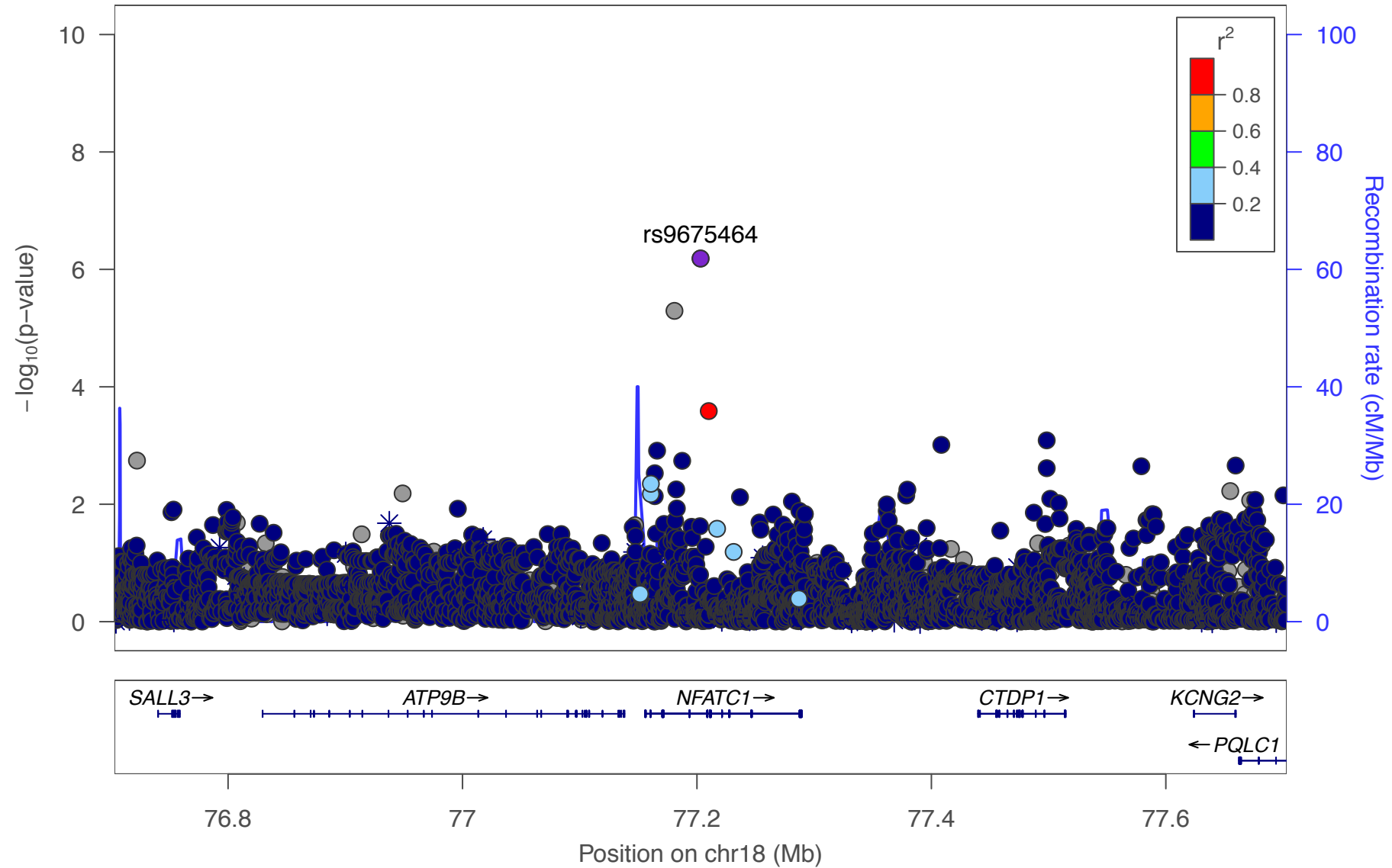

# OFC.MCL

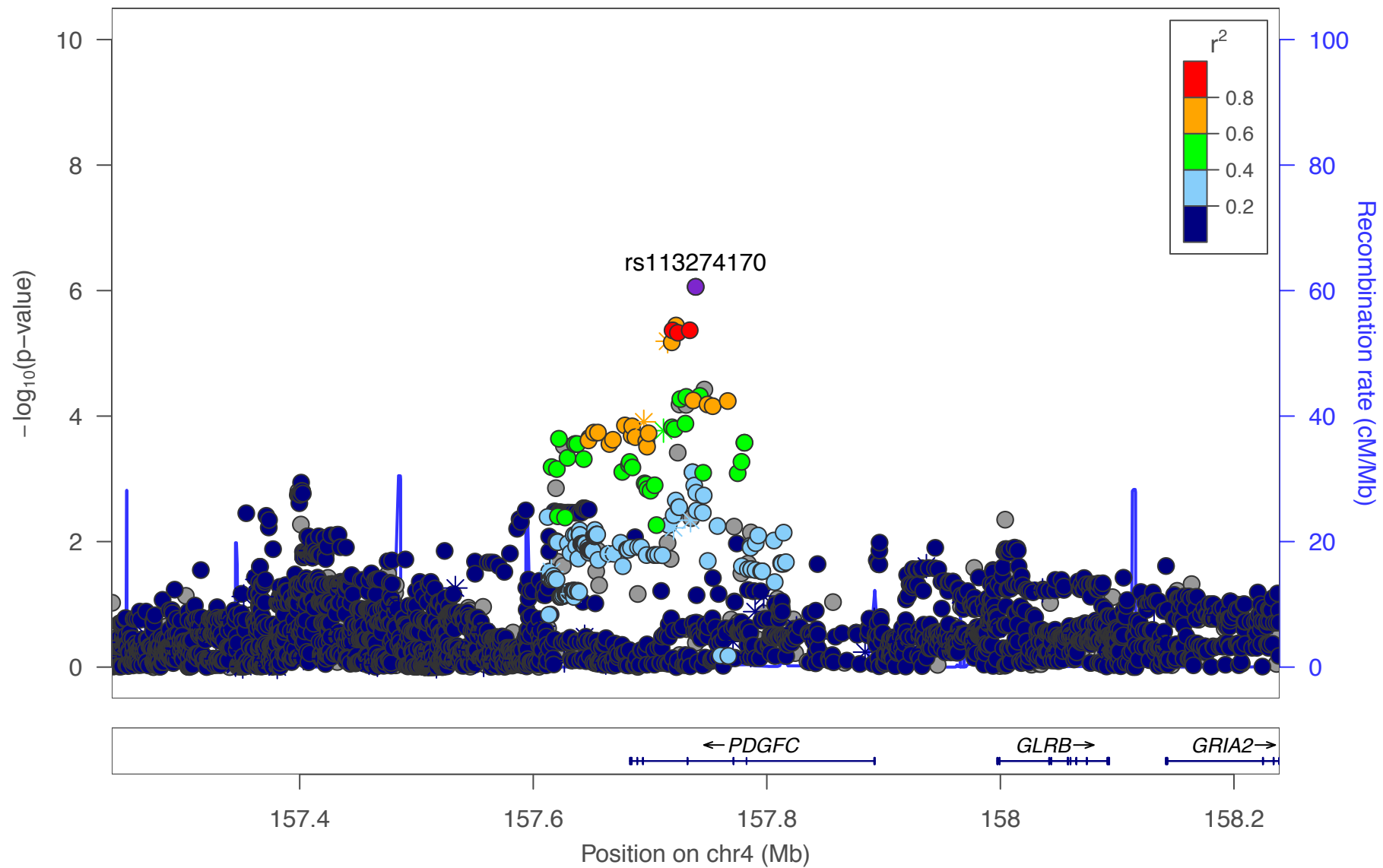

# OFC.MCL

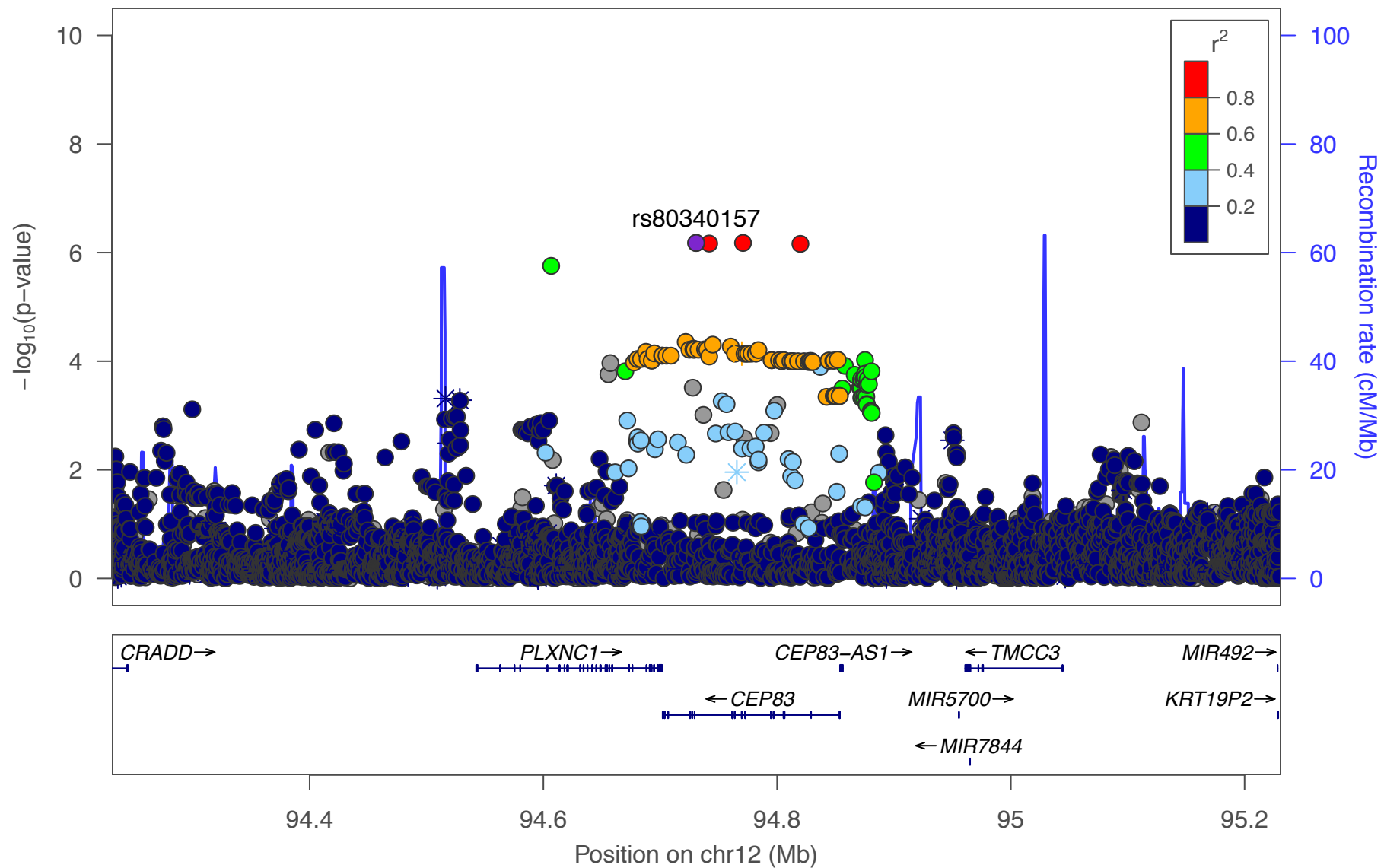

OFC.MCL

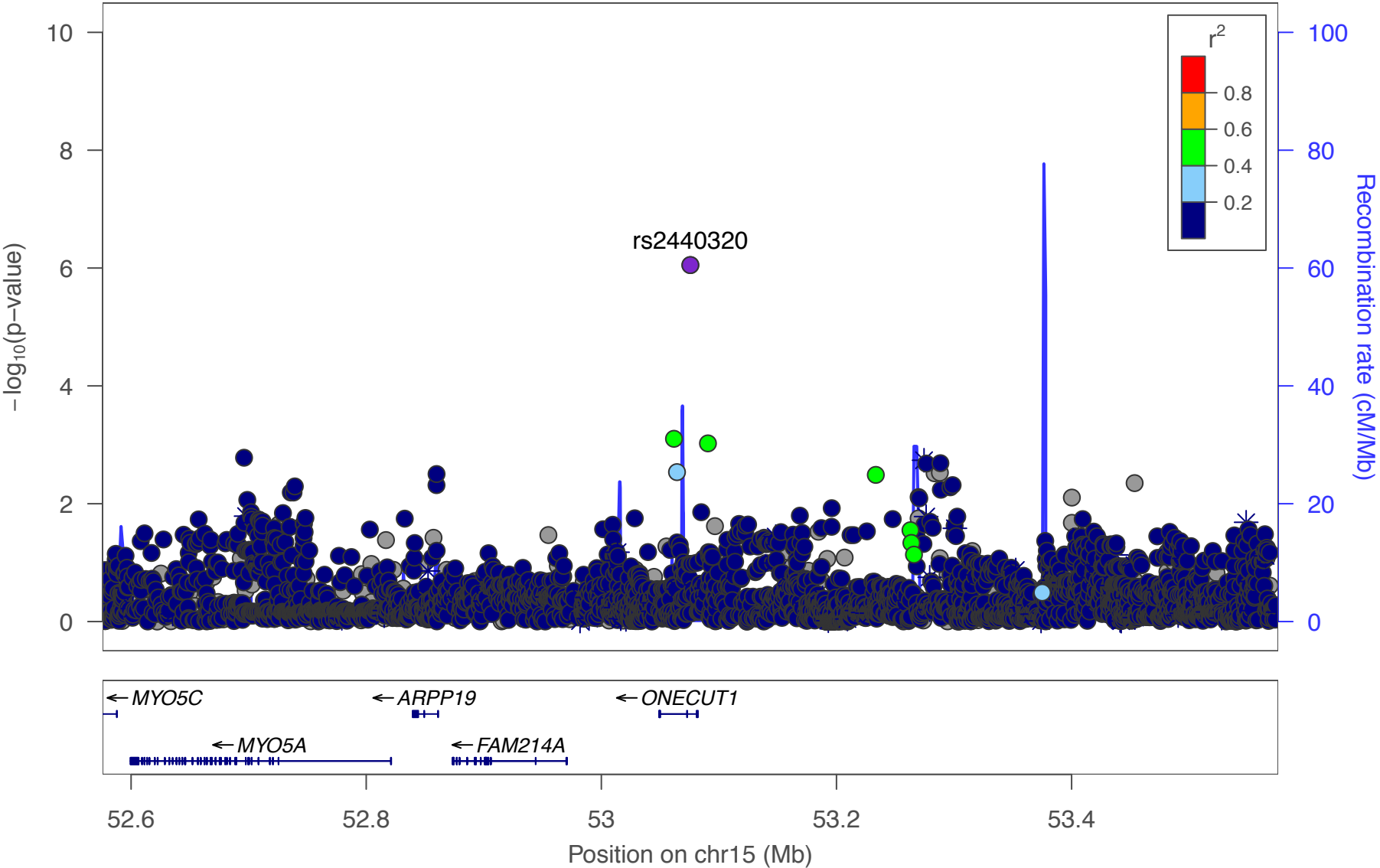

# OFC.MCL

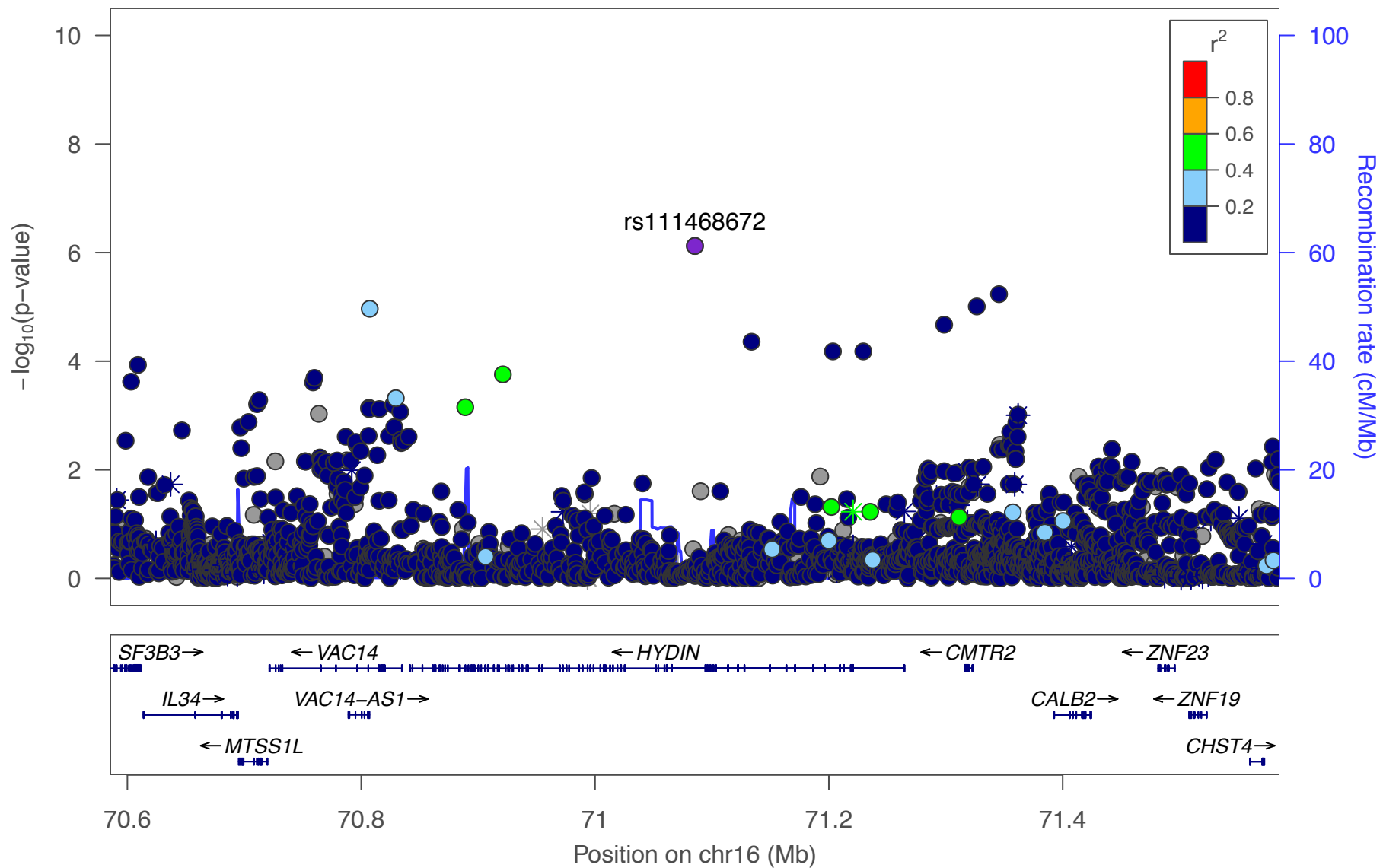

# OFC.MCL

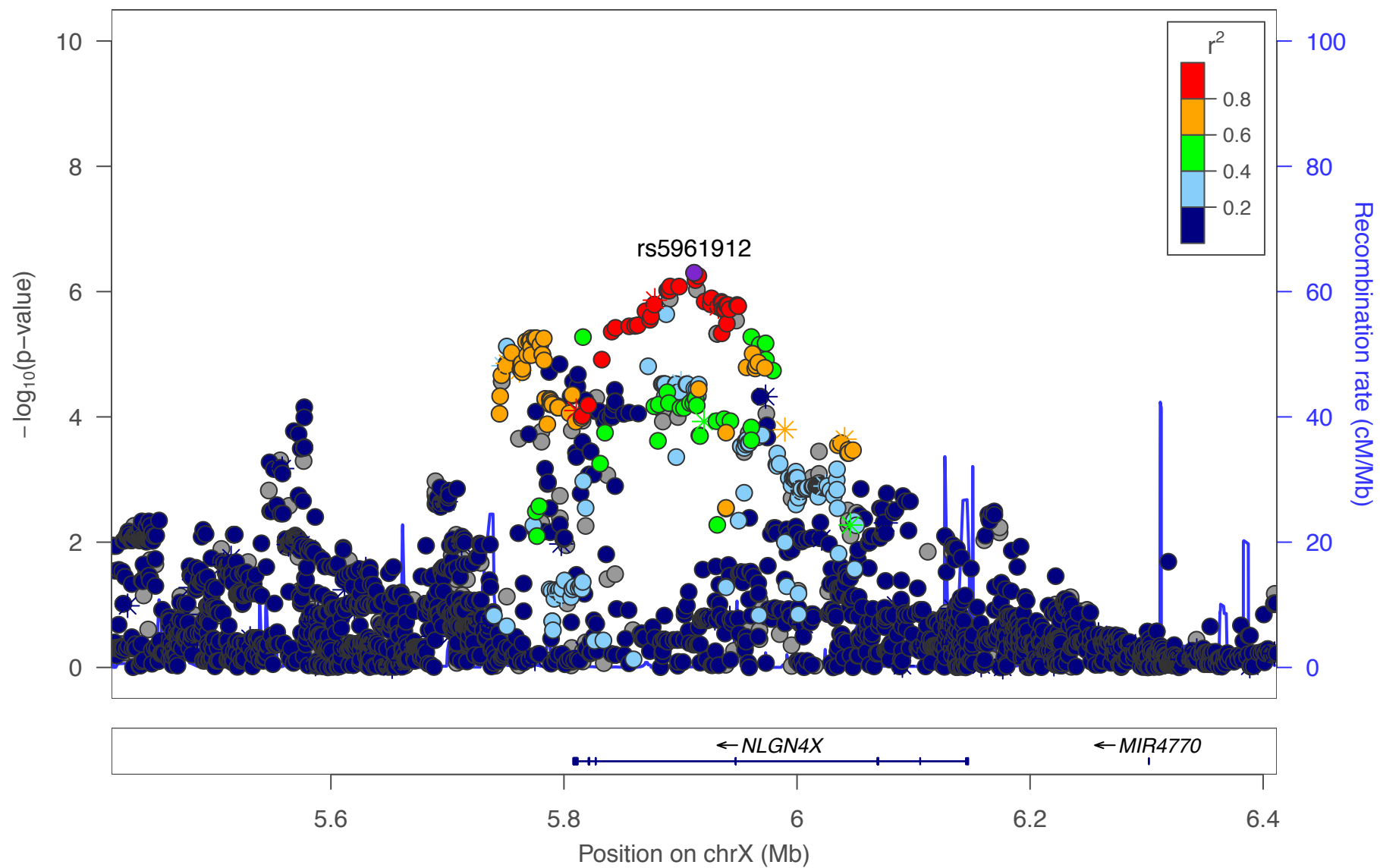

# OFC.CI

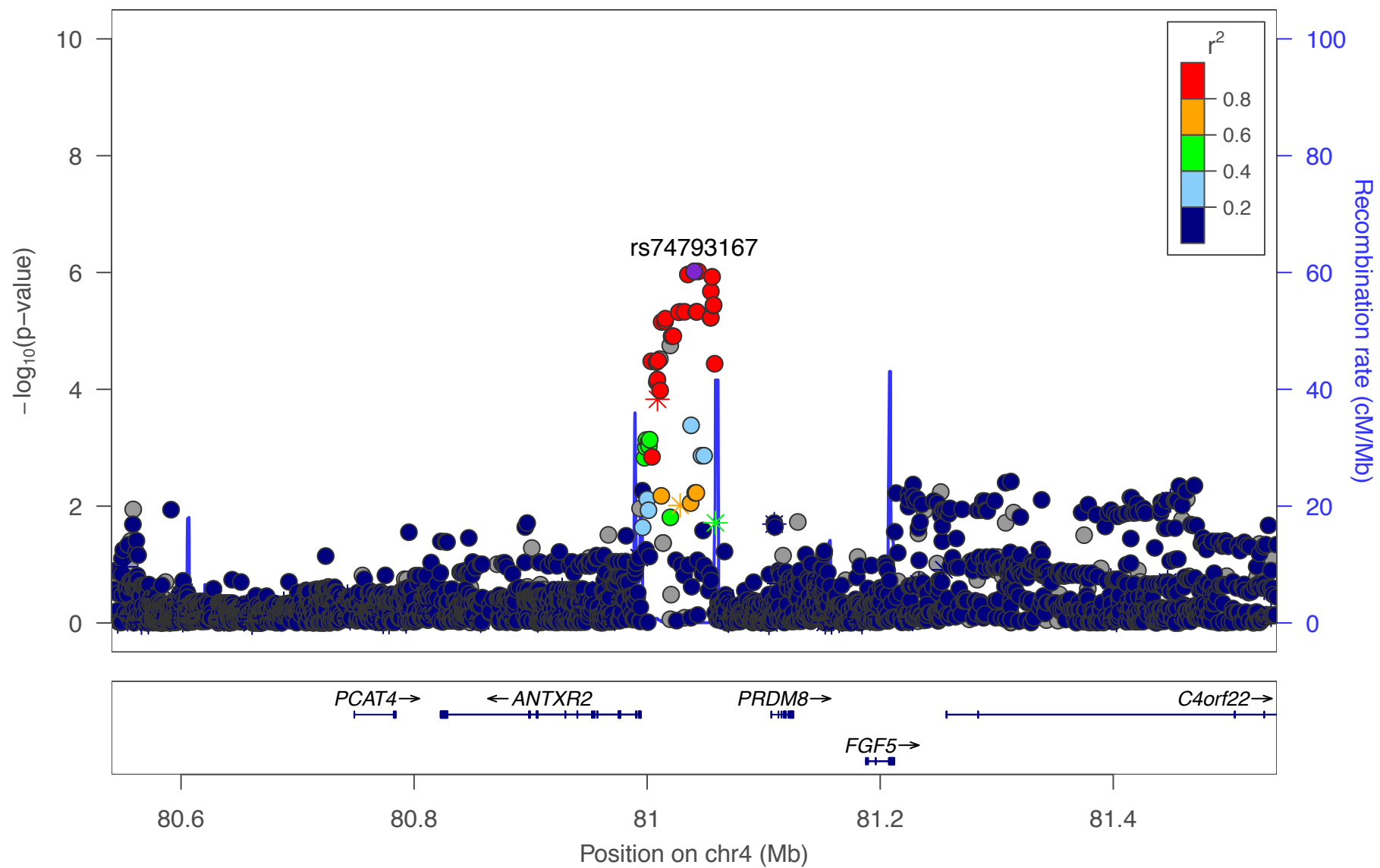

# OFC.CI

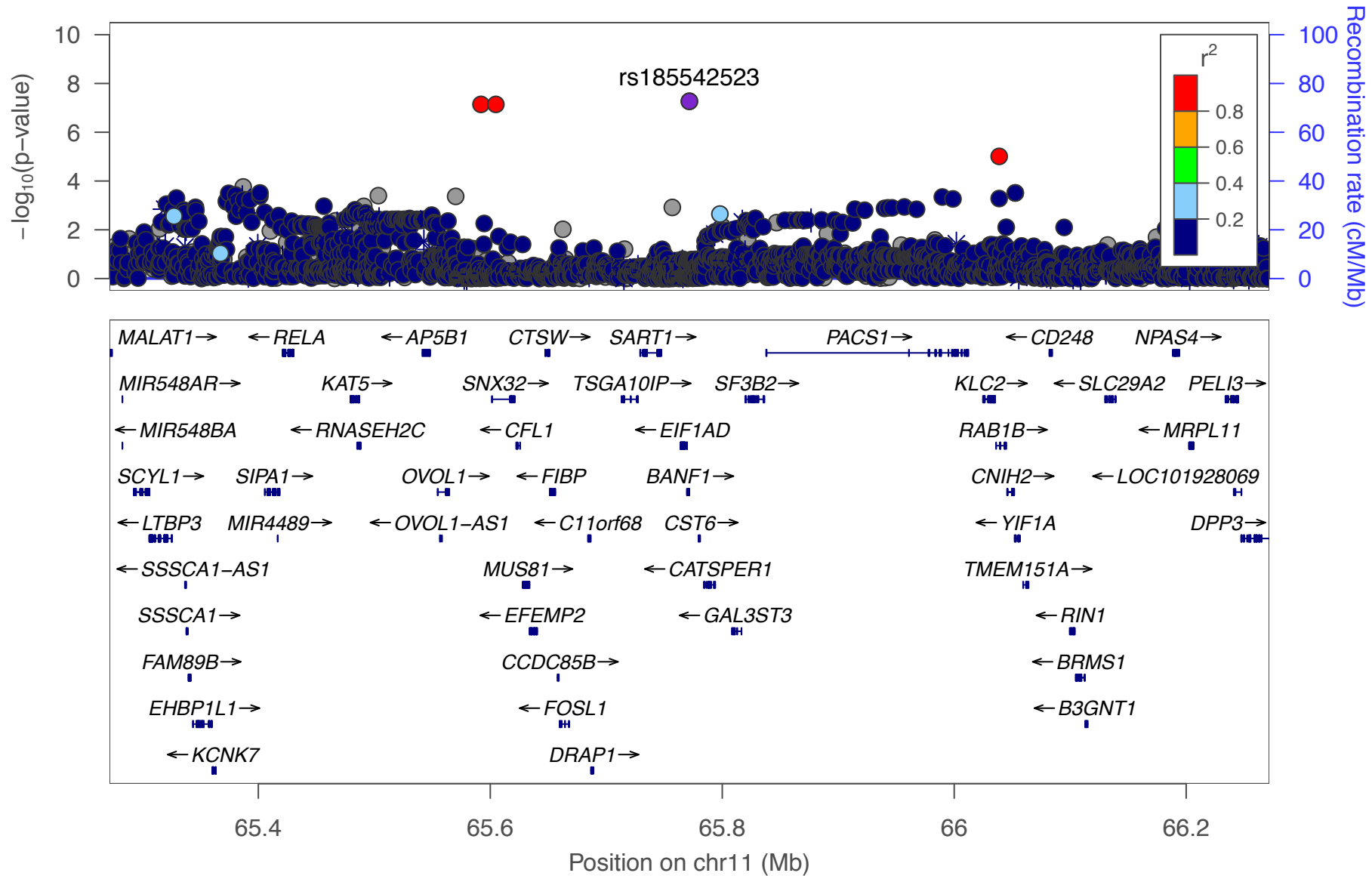

Supplement: S3 Fig — LocusZoom plots show the association (left y-axis; log10-transformed p-values) with facial traits. Genotyped SNPs are depicted by asterisks and imputed SNPs are depicted by circles. Shading of the points represent the linkage disequilibrium (r2, based on the 1000 Genomes Project Europeans) between each SNP and the top SNP, indicated by purple shading. Grey points in these plots represent the lack of LD information between the index SNP (diamond) the plotted SNP (circle or asterisk). The blue overlay shows the recombination rate (right y-axis). Positions of genes are shown below the plot. (PDF) [file pone.0196148.s011.pdf]

Figure S6

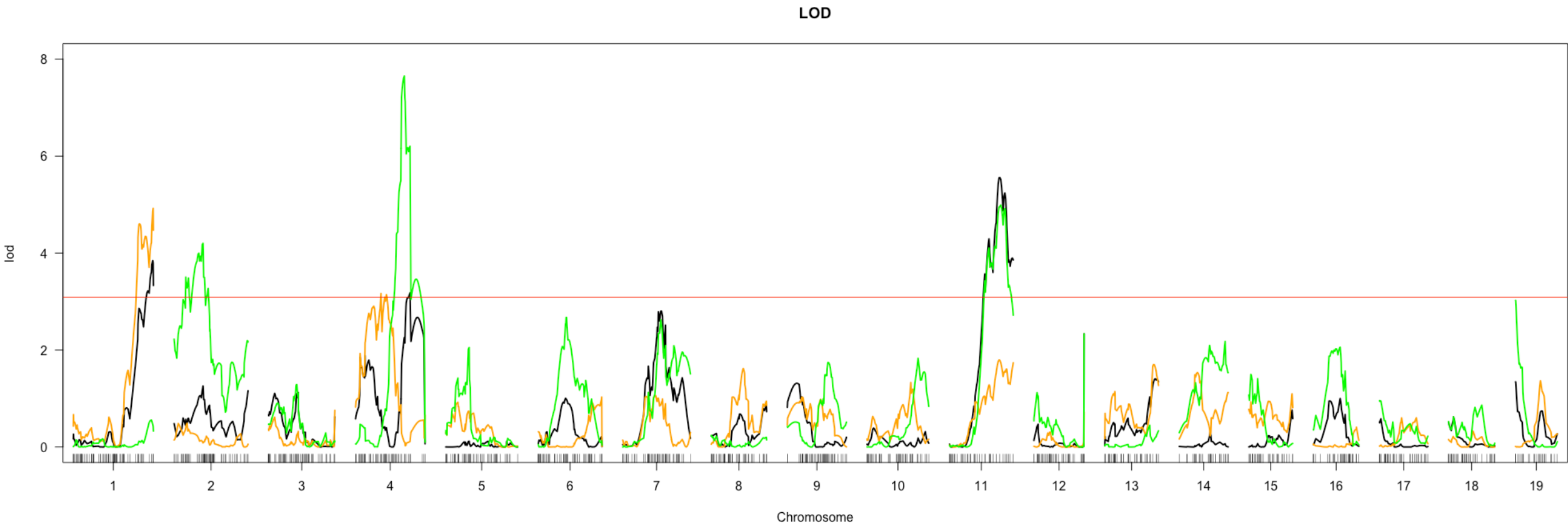

Supplement: S5 Fig — The three traits are indicated by color: MCW = Orange, MCL = Green, CI = Black. The horizontal line represents the permutation-based empirical threshold for genome-wide statistical significance. (PDF) [file pone.0196148.s013.pdf]

## OFC Cohort

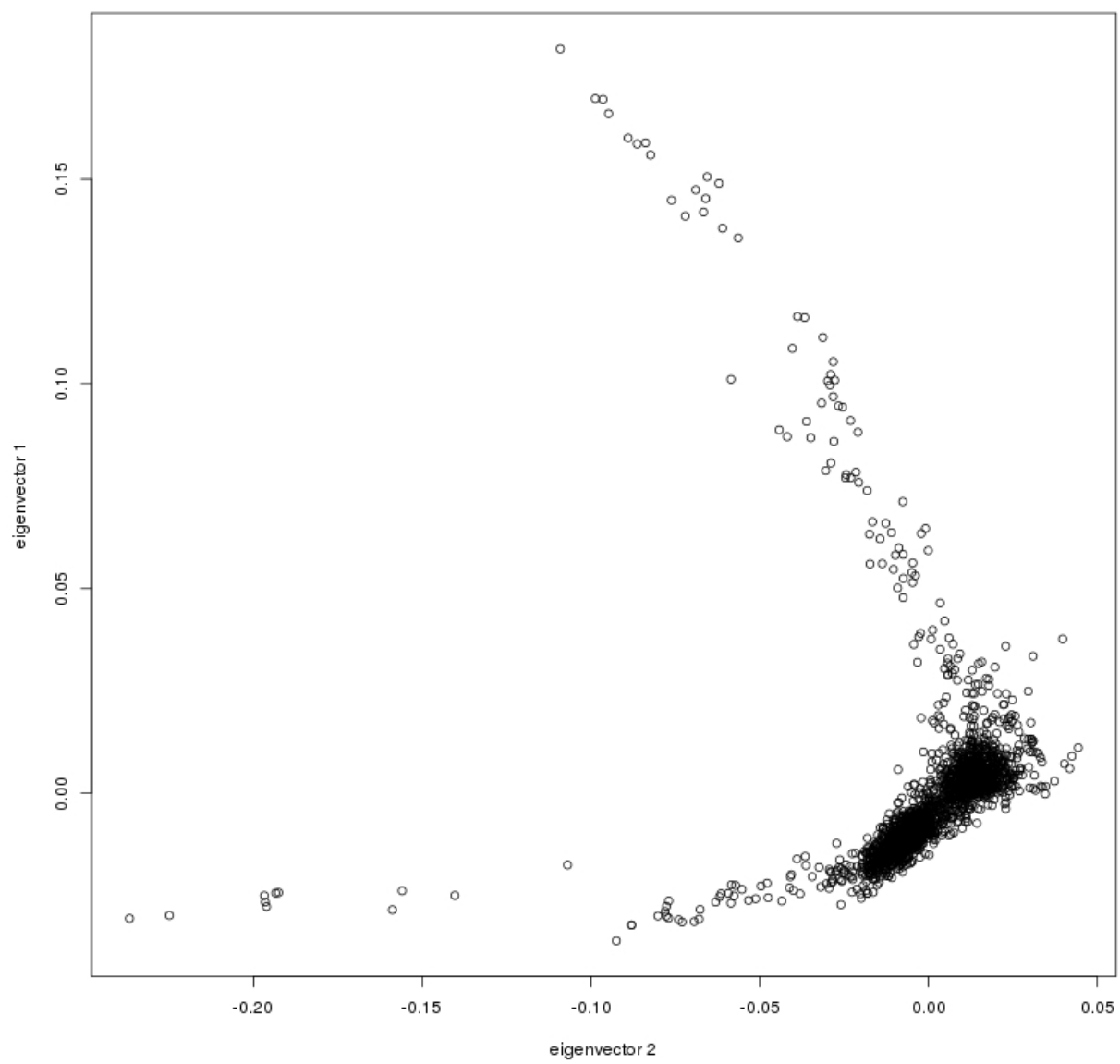

## OFC Cohort

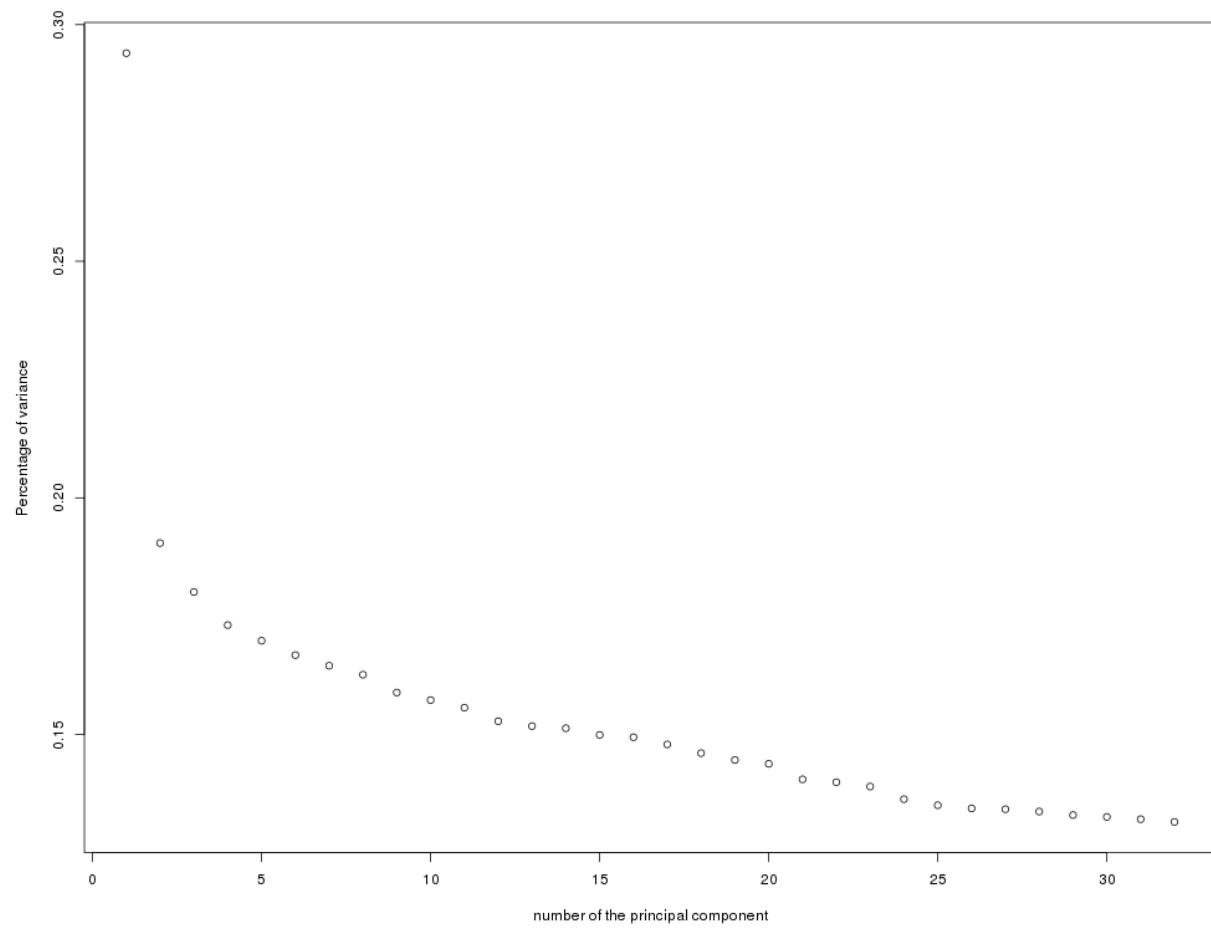

### 3DFN Cohort

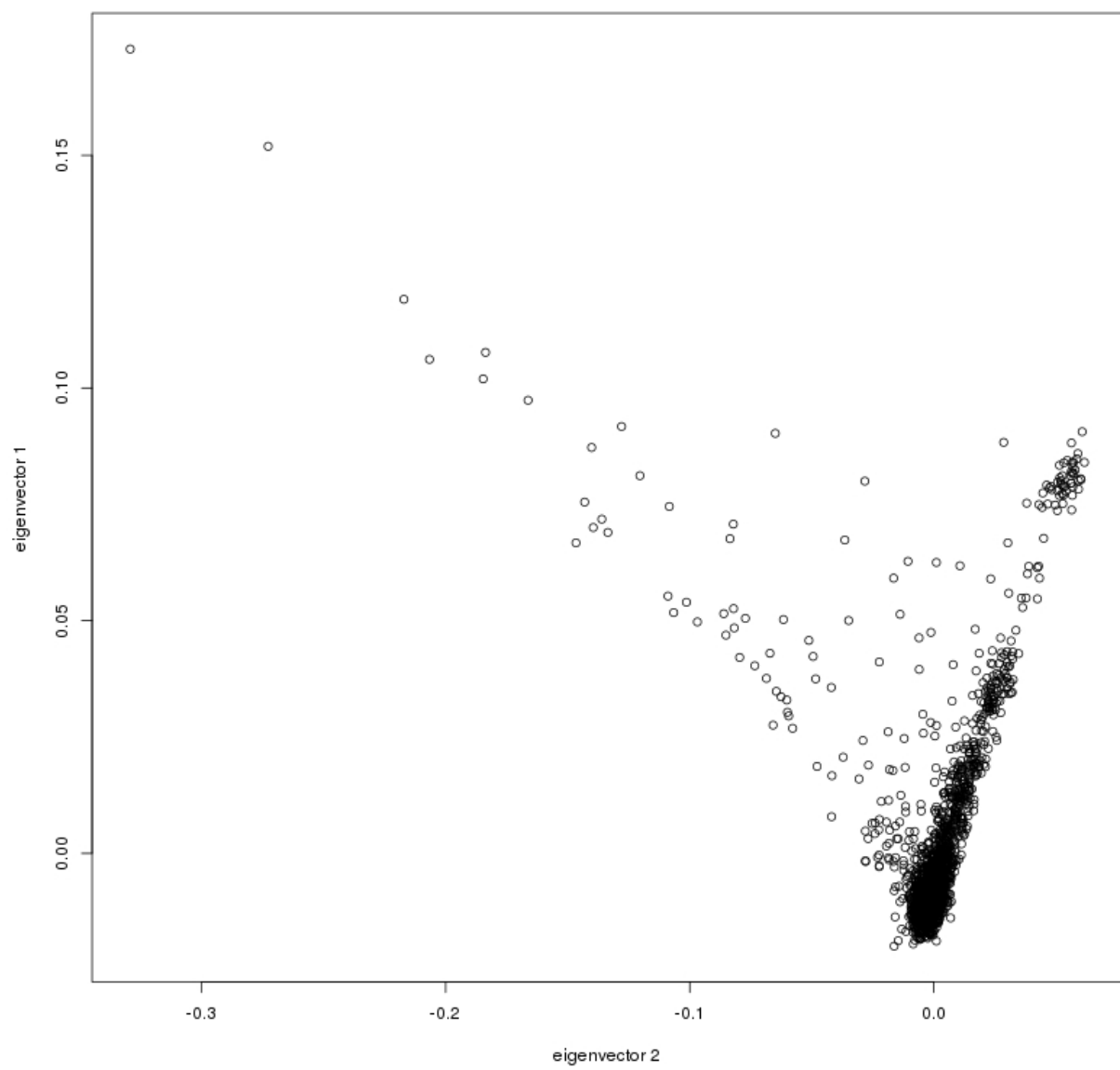

3DFN Cohort

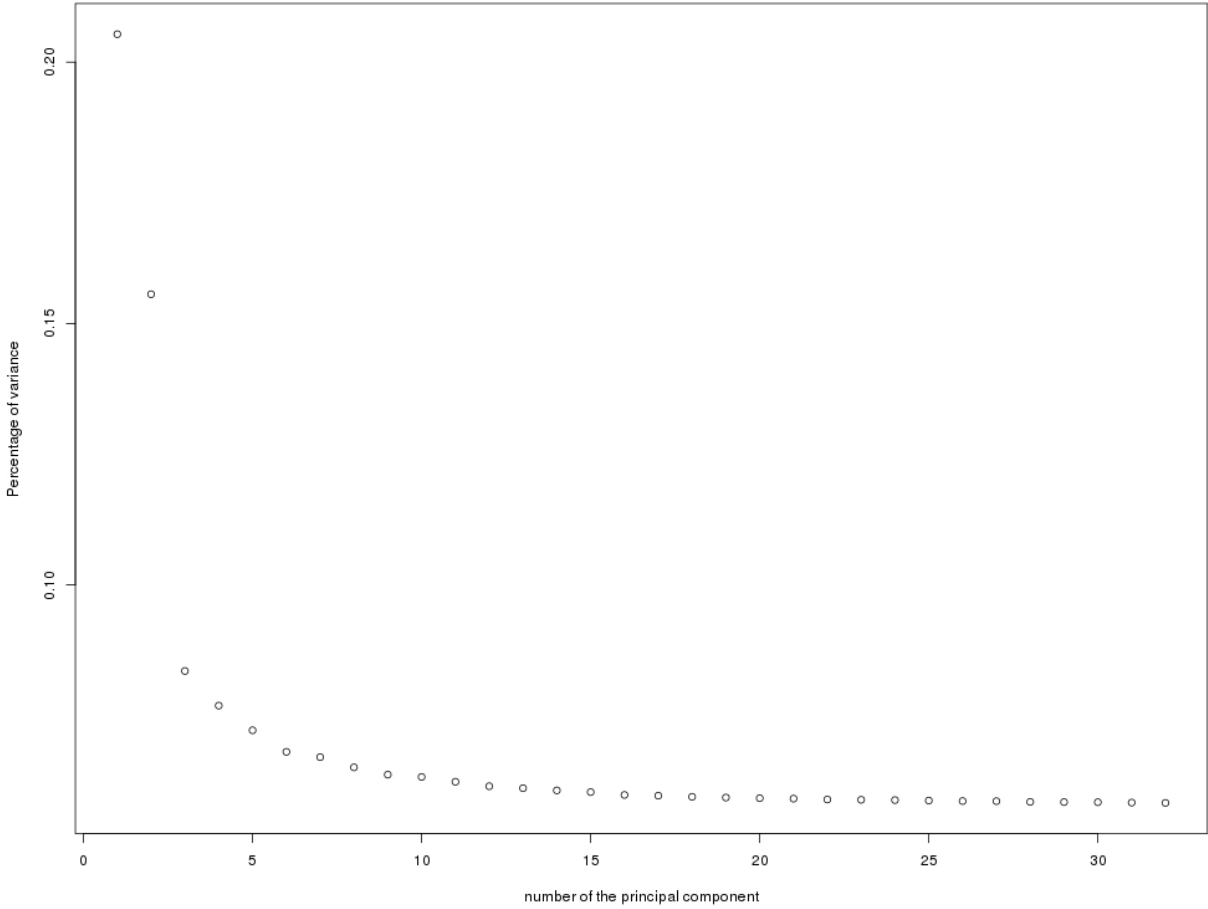

Supplement: S6 Fig — (PDF) [file pone.0196148.s014.pdf]

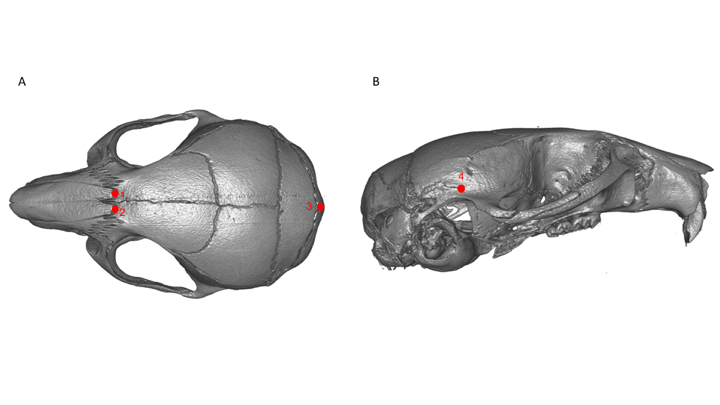

Supplement: S7 Fig — (TIFF) [file pone.0196148.s015.tiff]

OFC.CI

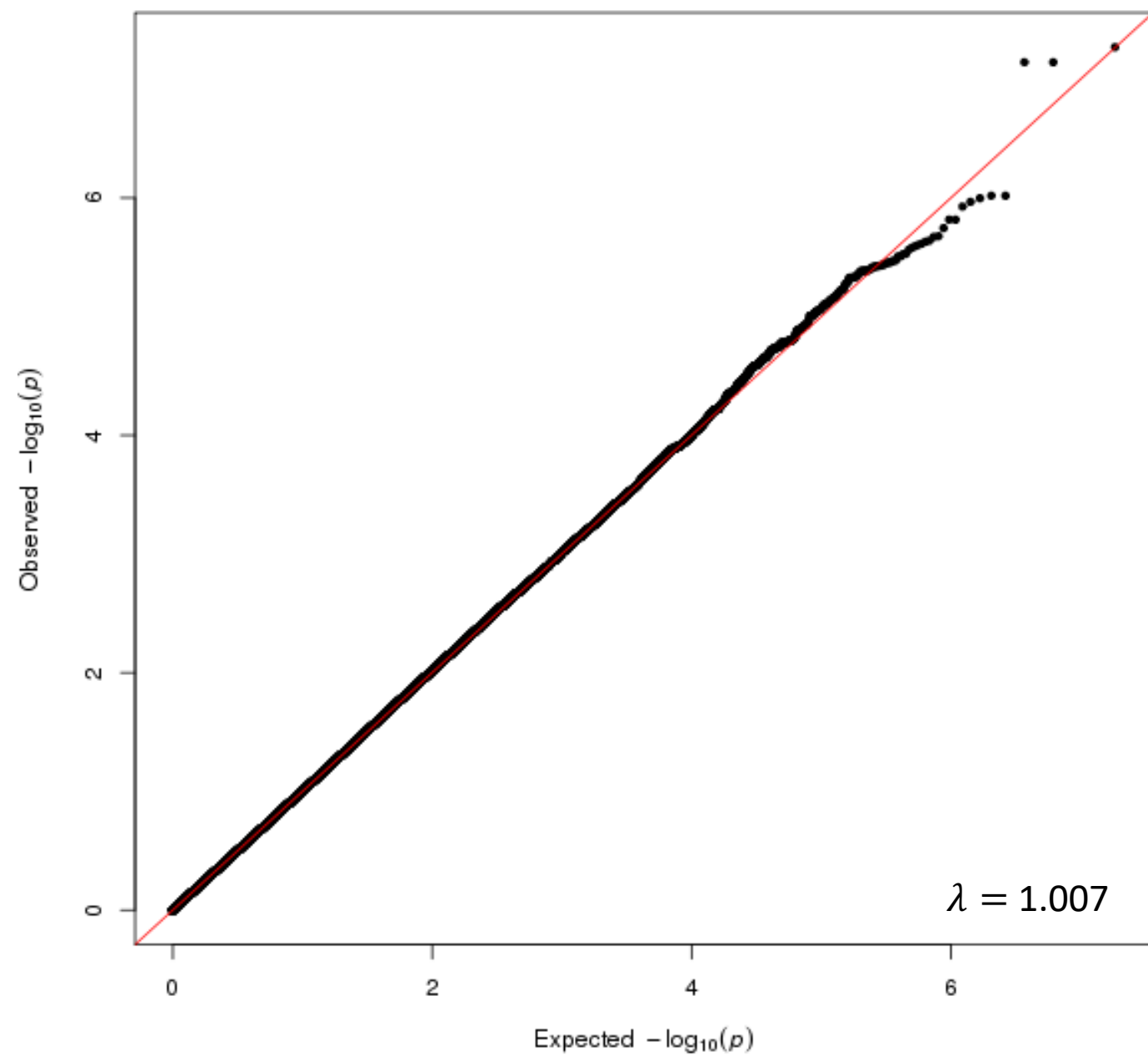

FV.CI

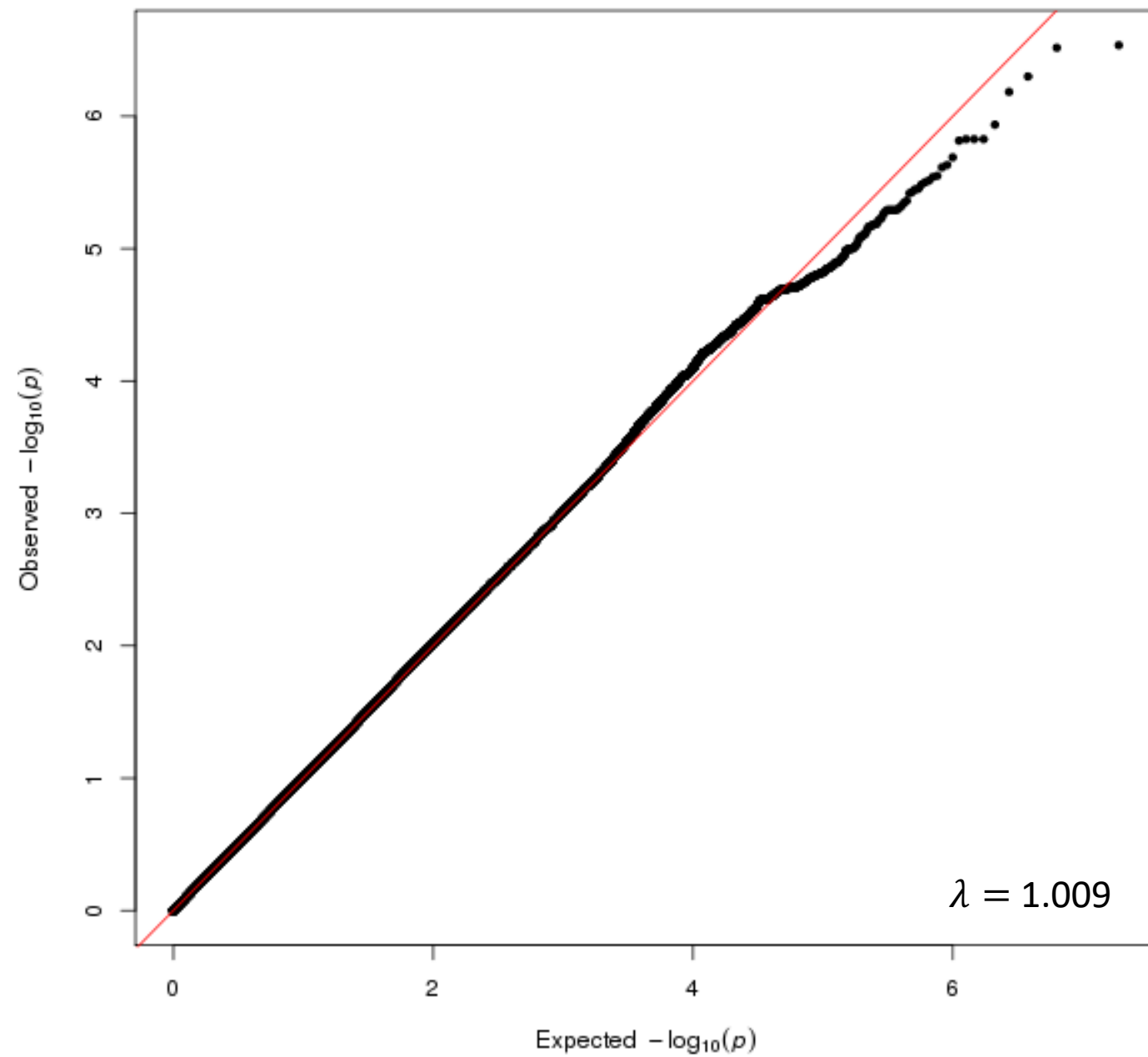

FV.MCL

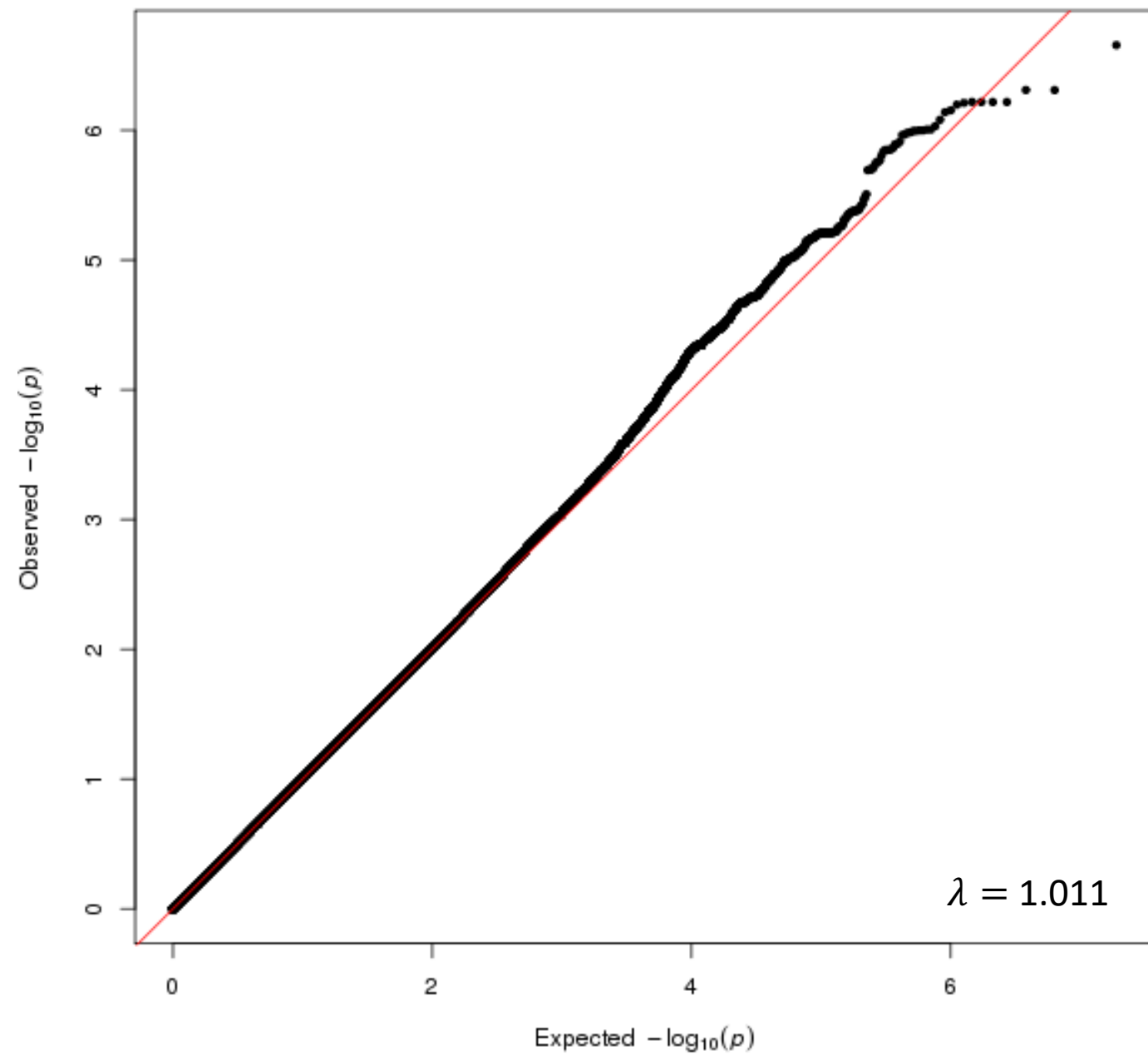

FV.MCW

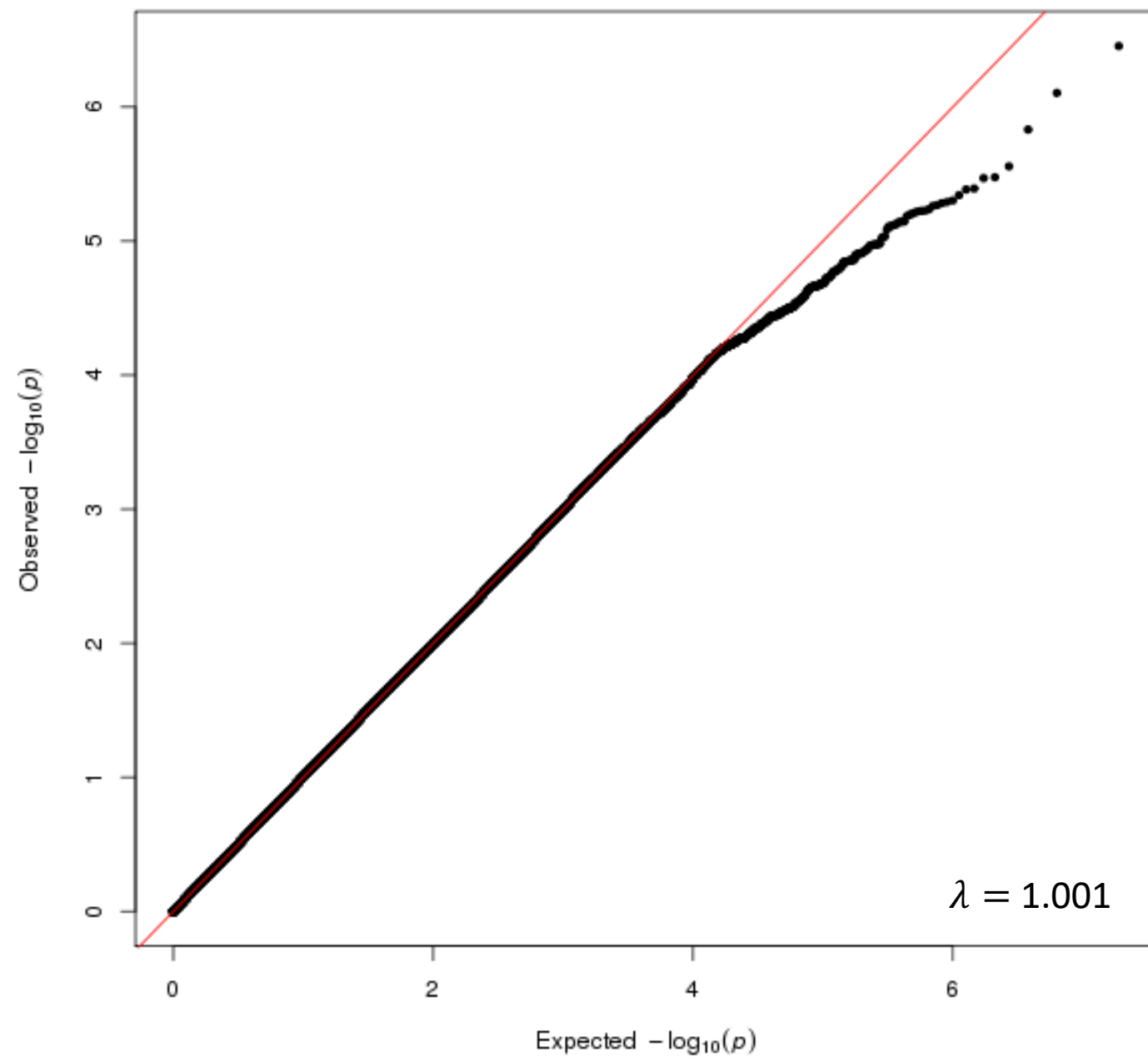

# OFC.MCL

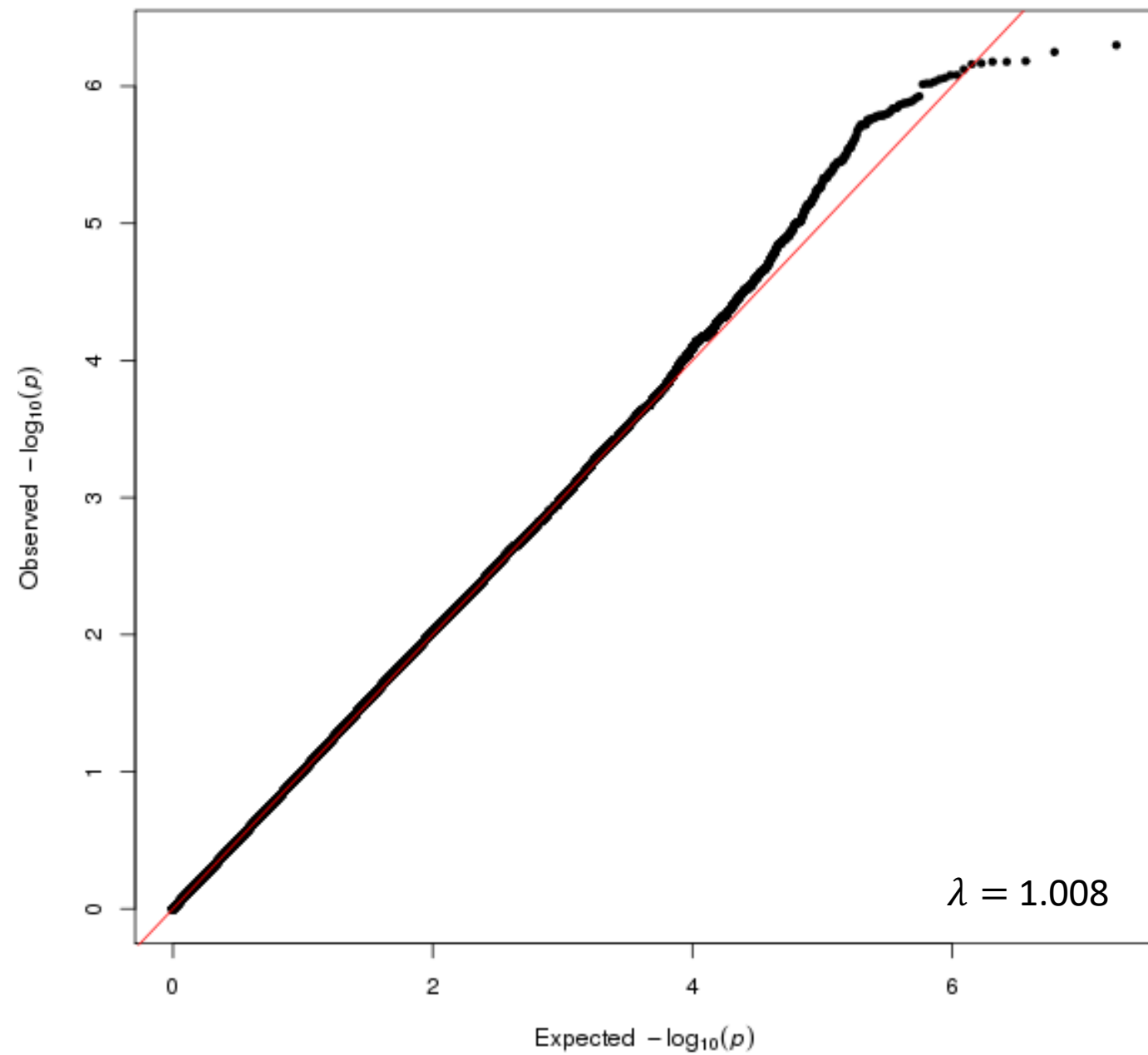

OFC.MCW

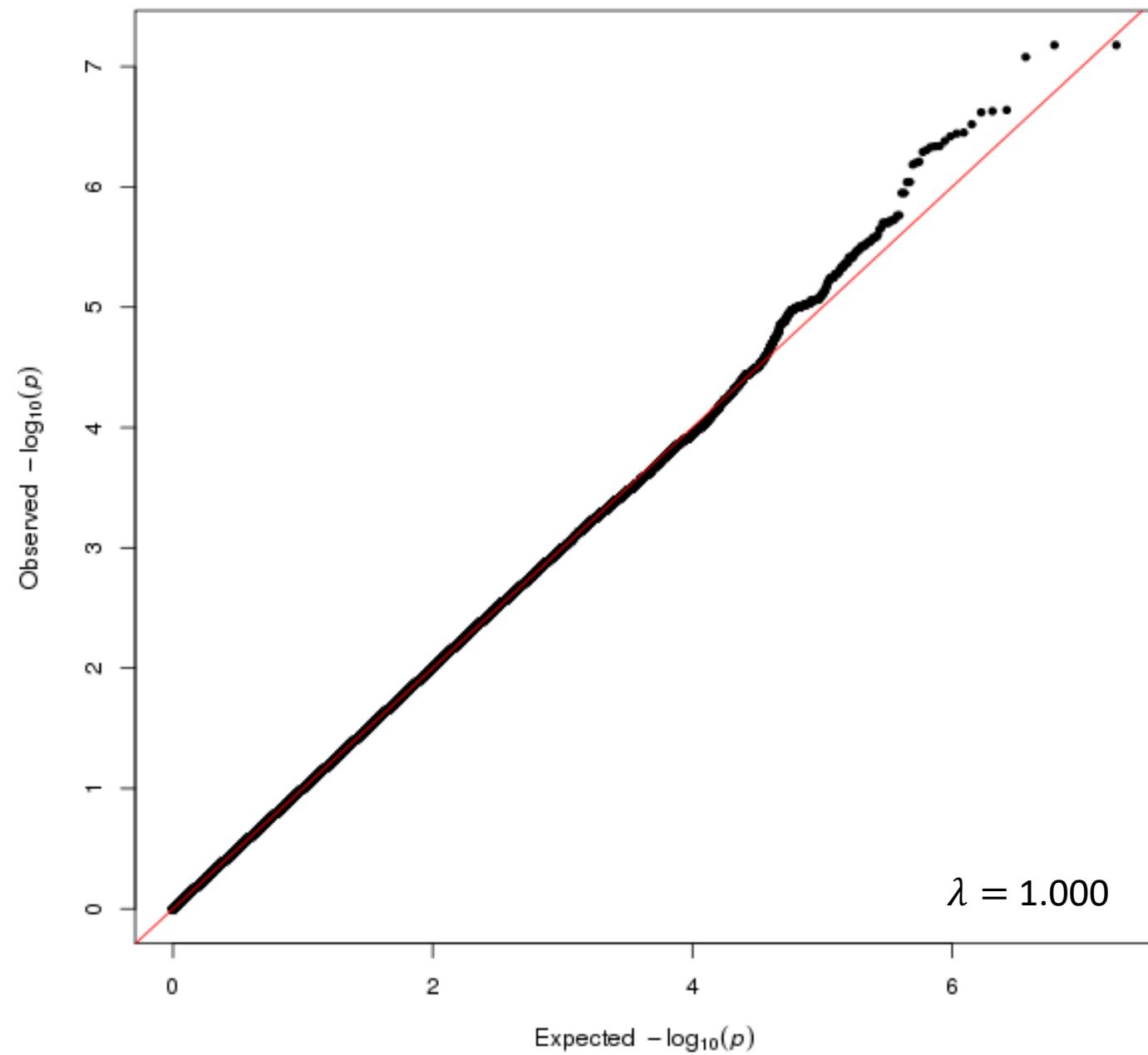

Meta.CI

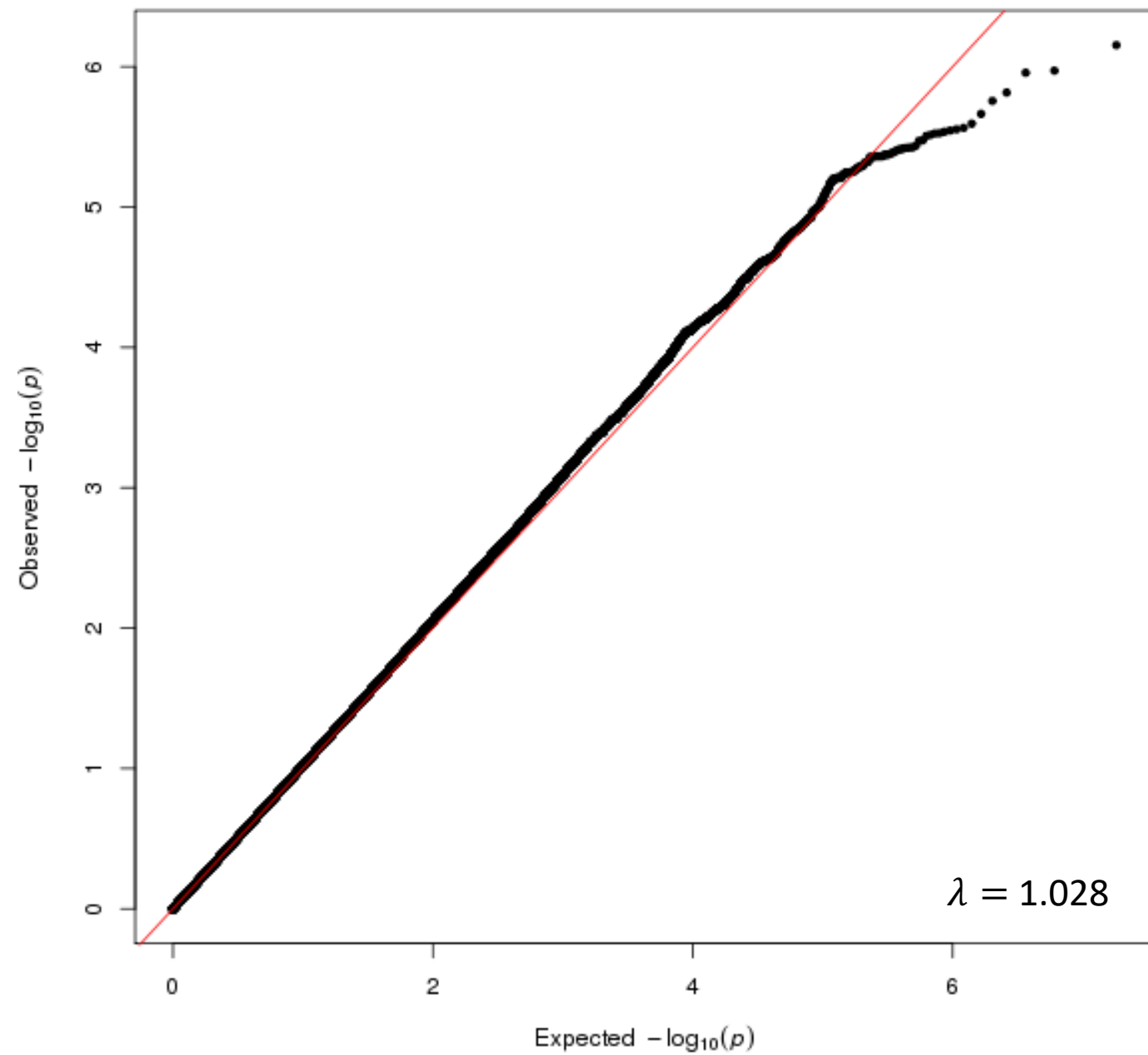

# Meta.MCL

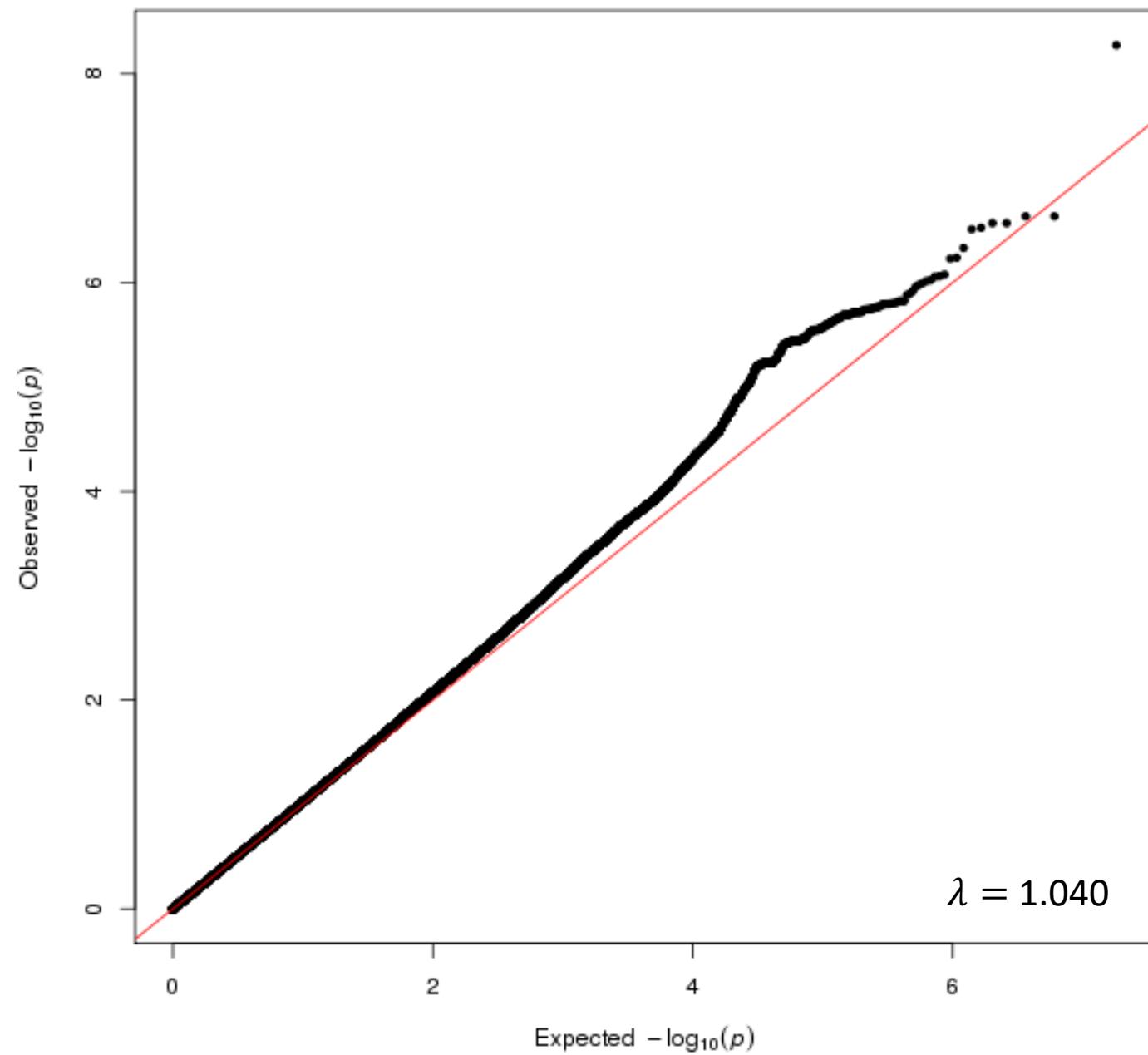

# Meta.MCW

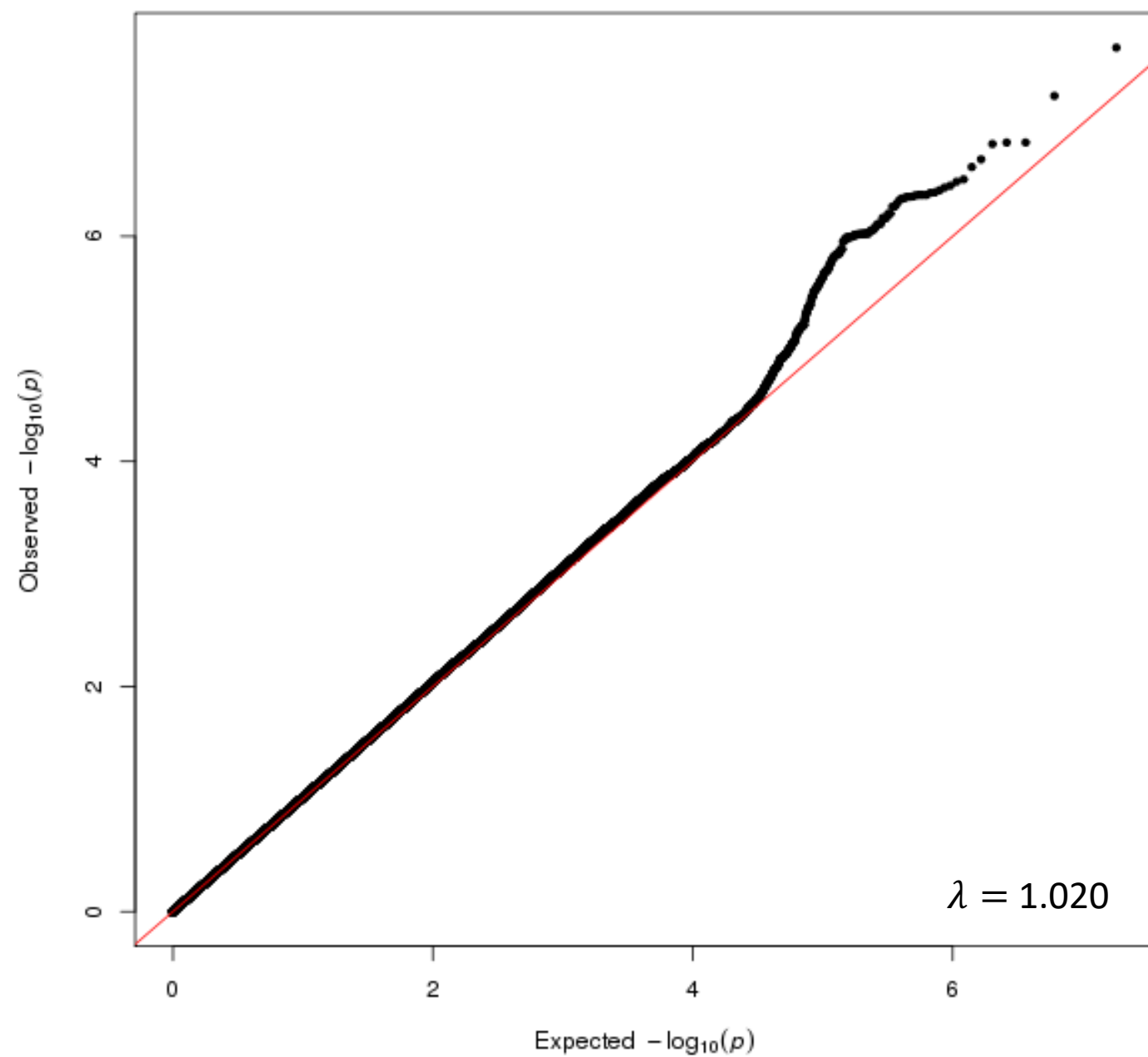

Supplement: S8 Fig — (PDF) [file pone.0196148.s016.pdf]
