# Supplementary material for: Mapping genetic variants for cranial vault shape in humans
Source: PLoS One. 2018 Apr 26;13(4):e0196148. doi: 10.1371/journal.pone.0196148 (PMC5919379; doi:10.1371/journal.pone.0196148)
Supplement: S4 Fig — LocusZoom plots show the association (left y-axis; log10-transformed p-values) with facial traits. Genotyped SNPs are depicted by asterisks and imputed SNPs are depicted by circles. Shading of the points represent the linkage disequilibrium (r2, based on the 1000 Genomes Project Europeans) between each SNP and the top SNP, indicated by purple shading. Grey points in these plots represent the lack of LD information between the index SNP (diamond) the plotted SNP (circle or asterisk). The blue overlay shows the recombination rate (right y-axis). Positions of genes are shown below the plot. (PDF) [file pone.0196148.s012.pdf]

Figure S5

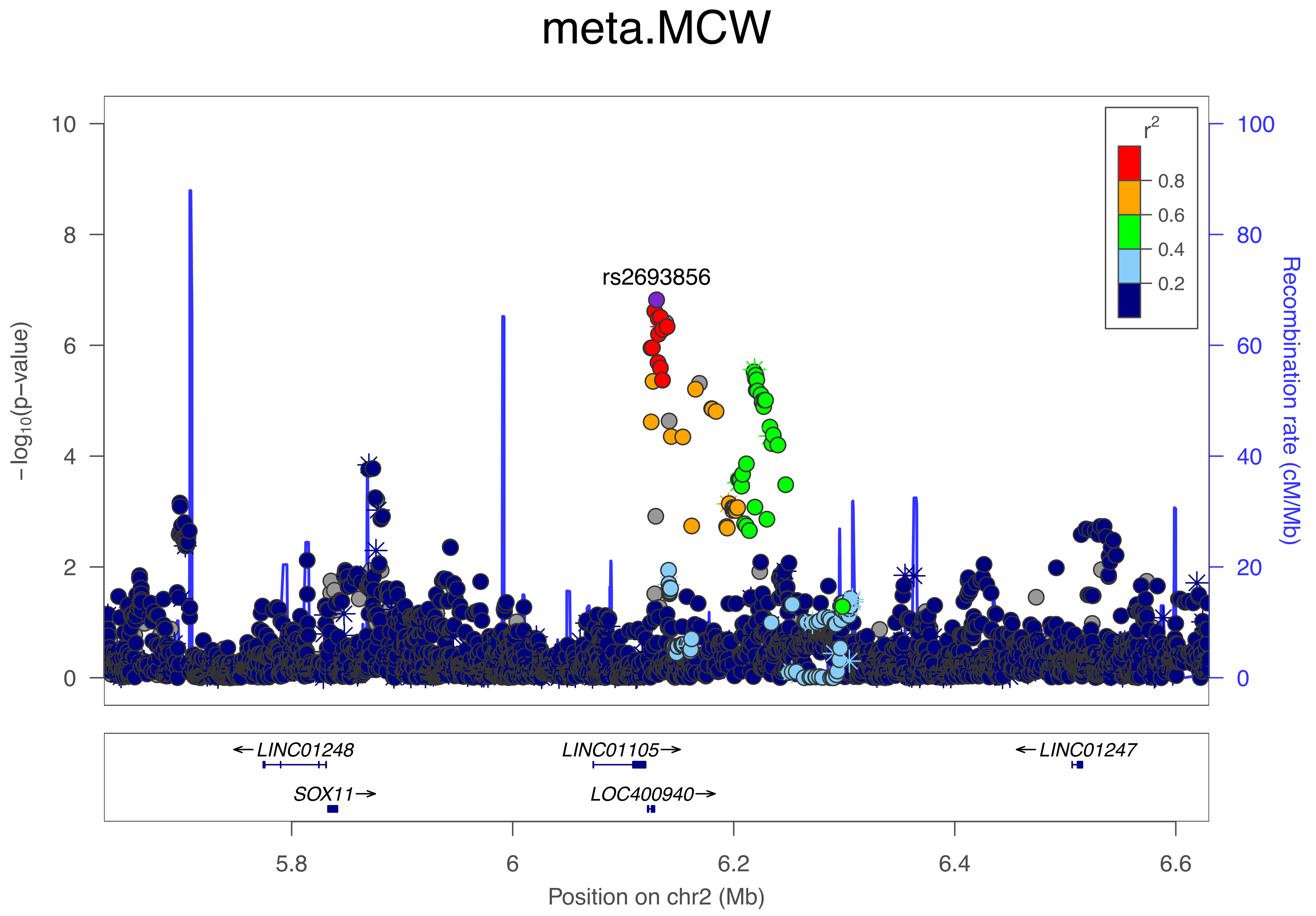
meta.MCW
rs2693856
r2
0.8
0.6
0.4
0.2
-log10(p-value)
Recombination rate (cM/Mb)
0
2
4
6
8
10
0
20
40
60
80
100
←LINC01248
LINC01105→
←LINC01247
SOX11→
LOC400940→
5.8
6
6.2
6.4
6.6
Position on chr2 (Mb)

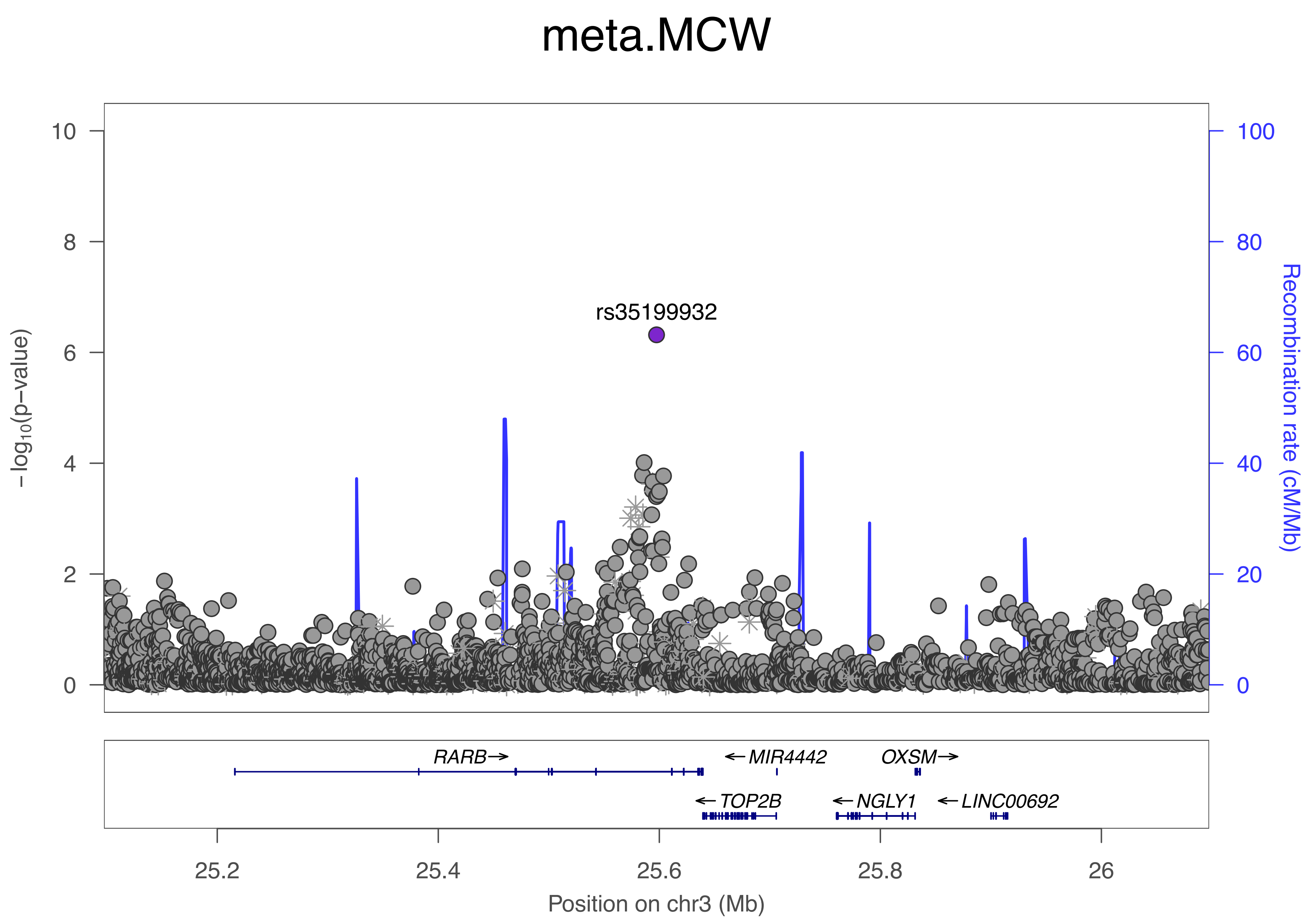
meta.MCW
rs35199932
−log10(p−value)
Recombination rate (cM/Mb)
0
2
4
6
8
10
0
20
40
60
80
100
RARB→
←MIR4442
OXSM→
←TOP2B
←NGLY1
←LINC00692
25.2
25.4
25.6
25.8
26
Position on chr3 (Mb)

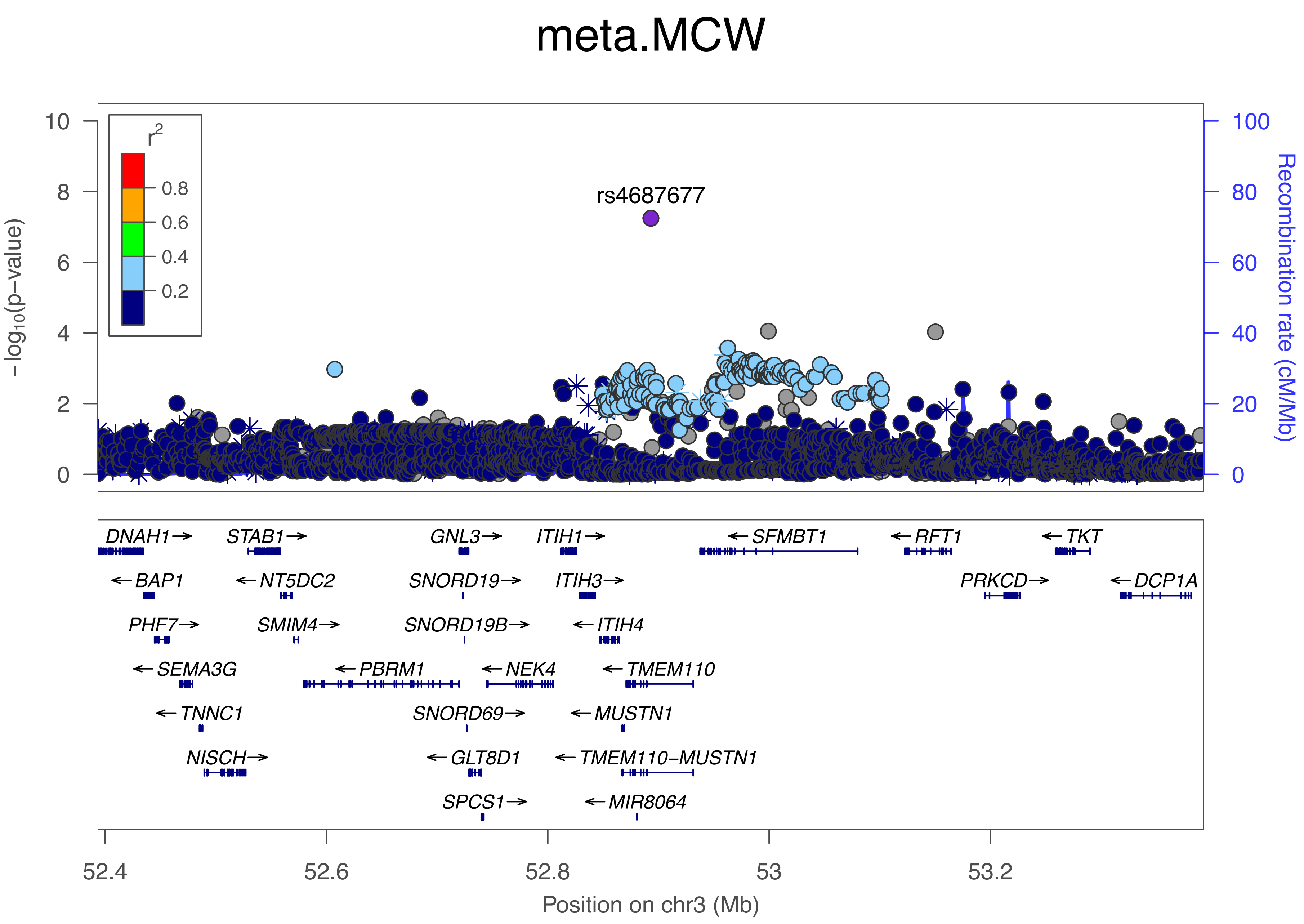

meta.MCW
rs4687677
r2
0.8
0.6
0.4
0.2
−log10(p−value)
Recombination rate (cM/Mb)
DNAH1→
STAB1→
GNL3→
ITIH1→
←SFMBT1
←RFT1
←TKT
←BAP1
←NT5DC2
SNORD19→
ITIH3→
PRKCD→
←DCP1A
PHF7→
SMIM4→
SNORD19B→
←ITIH4
←SEMA3G
←PBRM1
←NEK4
←TMEM110
←TNNC1
SNORD69→
←MUSTN1
NISCH→
←GLT8D1
←TMEM110−MUSTN1
SPCS1→
←MIR8064
52.4
52.6
52.8
53
53.2
Position on chr3 (Mb)

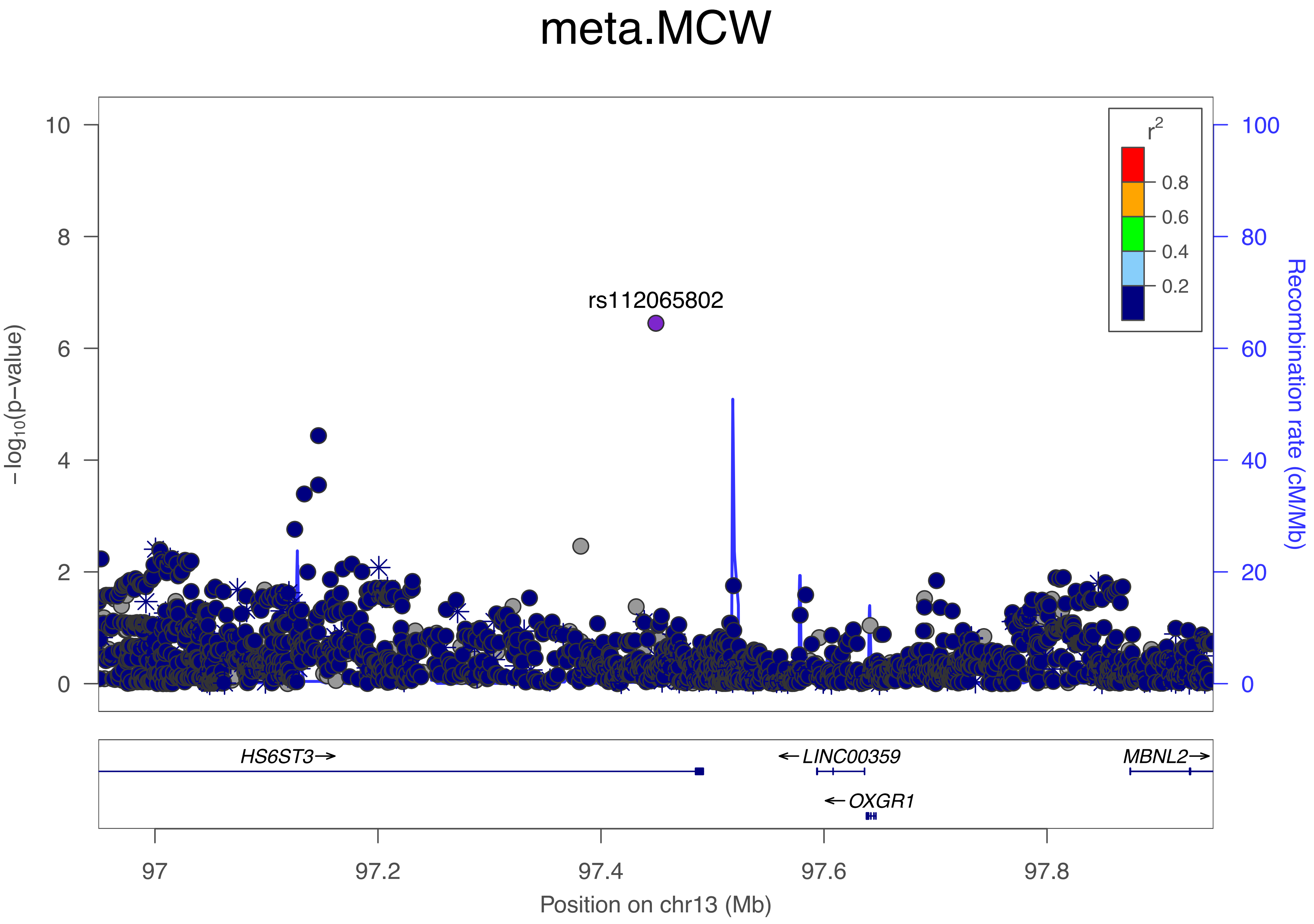

meta.MCW
rs112065802
r2
0.8
0.6
0.4
0.2
−log10(p−value)
Recombination rate (cM/Mb)
HS6ST3→
←LINC00359
←OXGR1
MBNL2→
97
97.2
97.4
97.6
97.8
Position on chr13 (Mb)

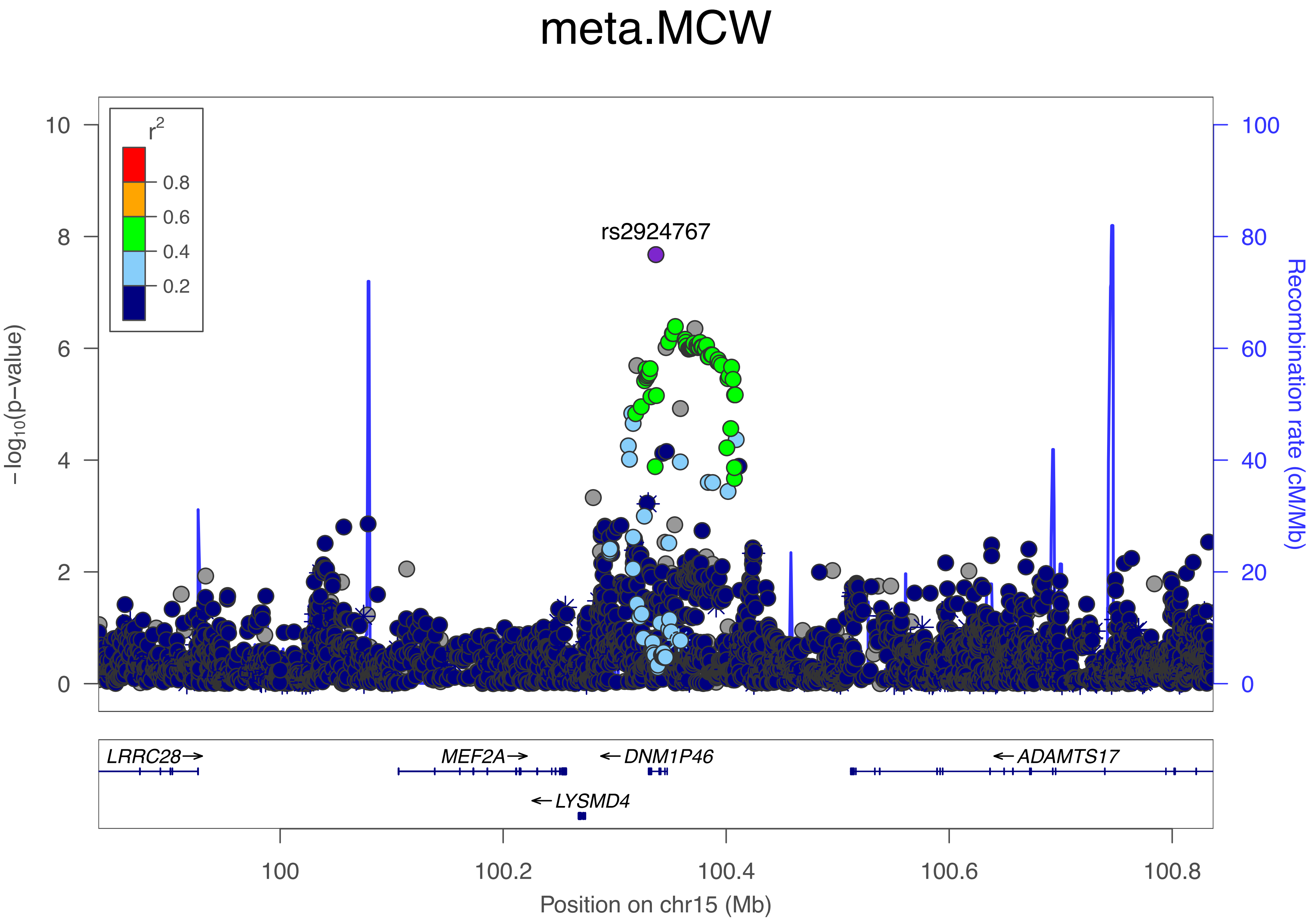

meta.MCW
r2
0.8
0.6
0.4
0.2
rs2924767
10
8
6
4
2
0
-log10(p-value)
100
80
60
40
20
0
Recombination rate (cM/Mb)
LRRC28→
MEF2A→
←DNM1P46
←ADAMTS17
←LYSMD4
100
100.2
100.4
100.6
100.8
Position on chr15 (Mb)

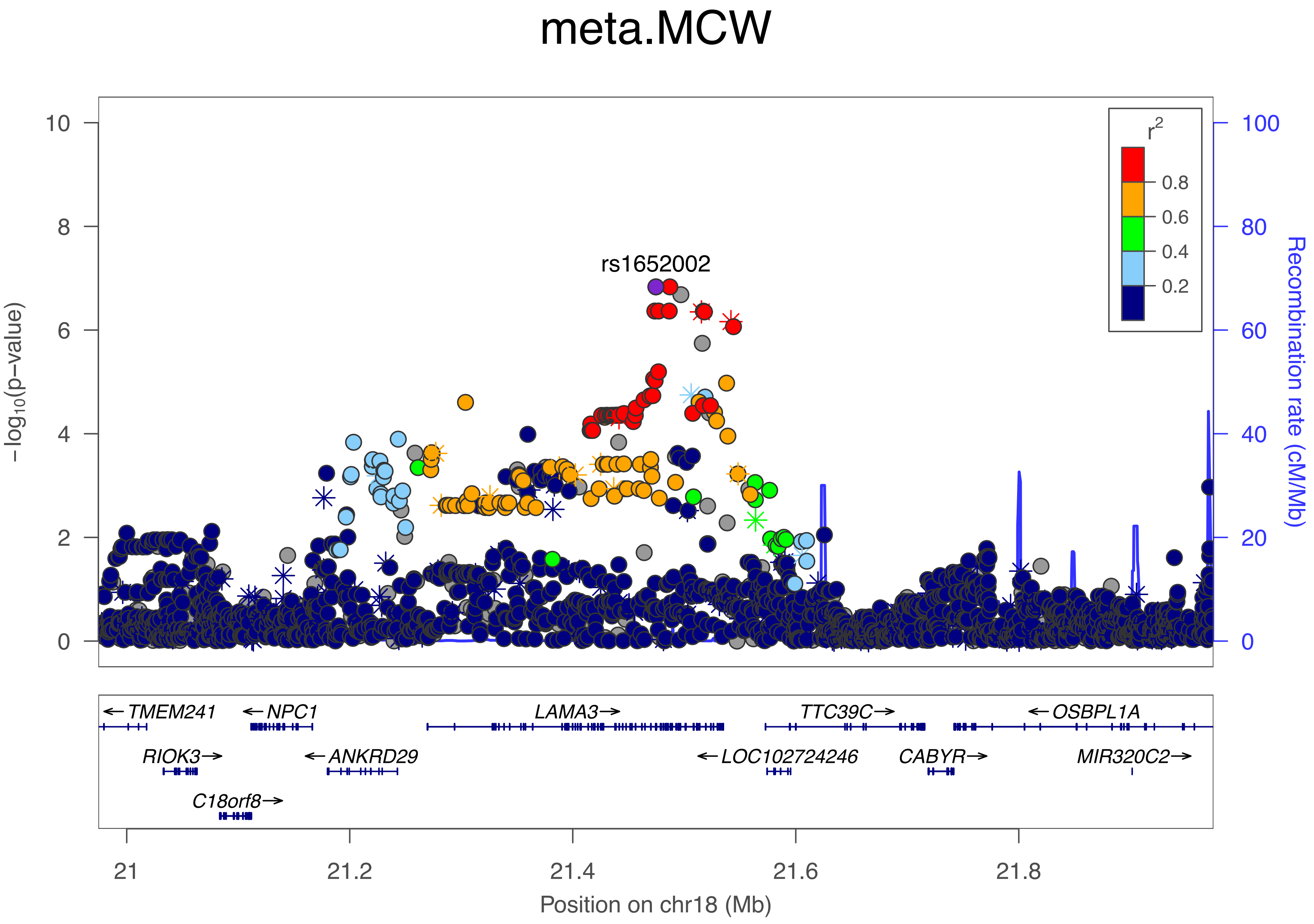

meta.MCW
rs1652002
r2
0.8
0.6
0.4
0.2
−log10(p−value)
Recombination rate (cM/Mb)
10
8
6
4
2
0
100
80
60
40
20
0
← TMEM241
← NPC1
LAMA3→
TTC39C→
← OSBPL1A
RIOK3→
← ANKRD29
← LOC102724246
CABYR→
MIR320C2→
C18orf8→
21
21.2
21.4
21.6
21.8
Position on chr18 (Mb)

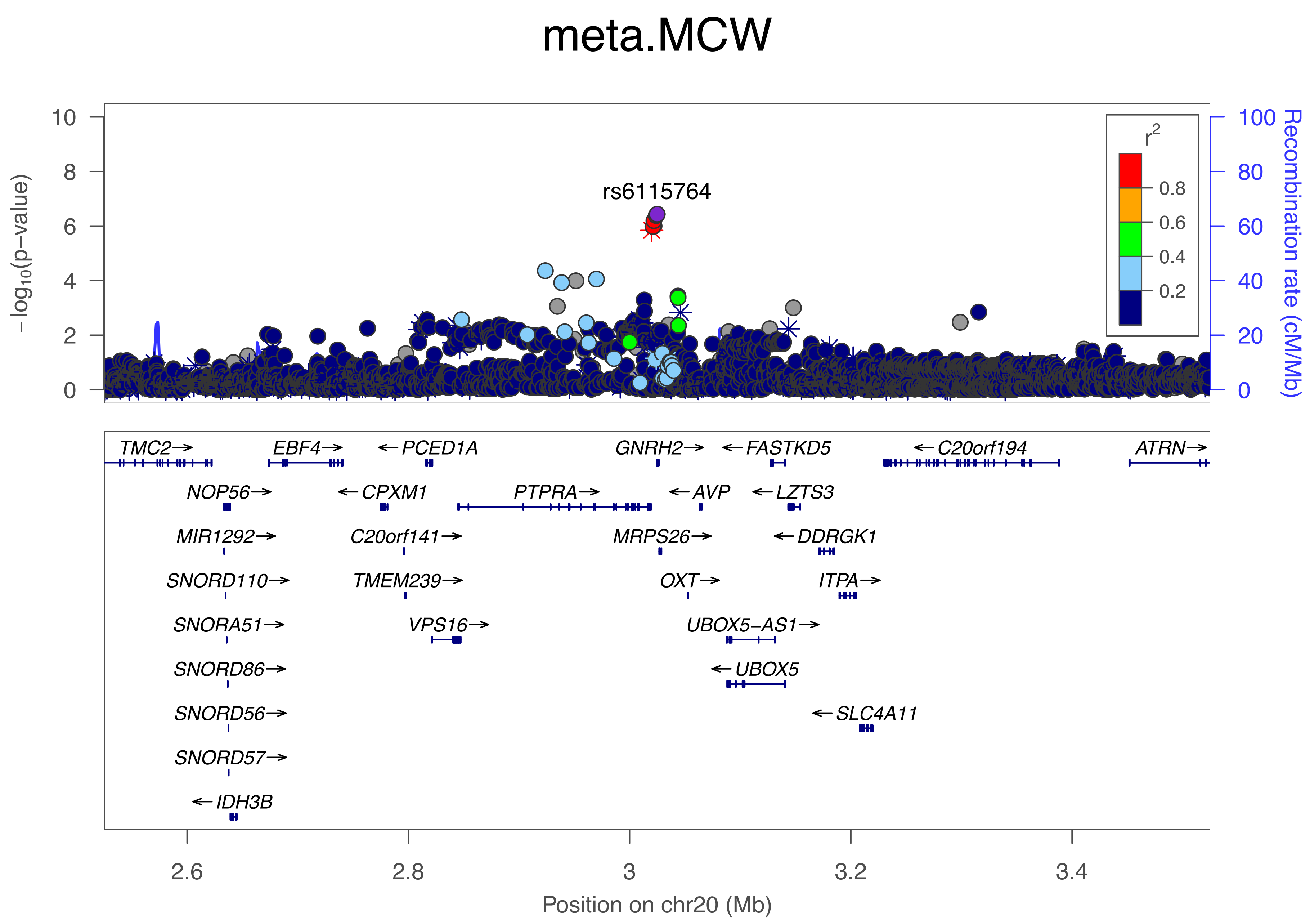
meta.MCW
rs6115764
-log10(p-value)
Recombination rate (cM/Mb)
r2
0.8
0.6
0.4
0.2
TMC2
EBF4
PCED1A
GNRH2
FASTKD5
C20orf194
ATRN
NOP56
CPXM1
PTPRA
AVP
LZTS3
MIR1292
C20orf141
MRPS26
DDRGK1
SNORD110
TMEM239
OXT
ITPA
SNORA51
VPS16
UBOX5-AS1
SNORD86
UBOX5
SNORD56
SLC4A11
SNORD57
IDH3B
2.6
2.8
3
3.2
3.4
Position on chr20 (Mb)

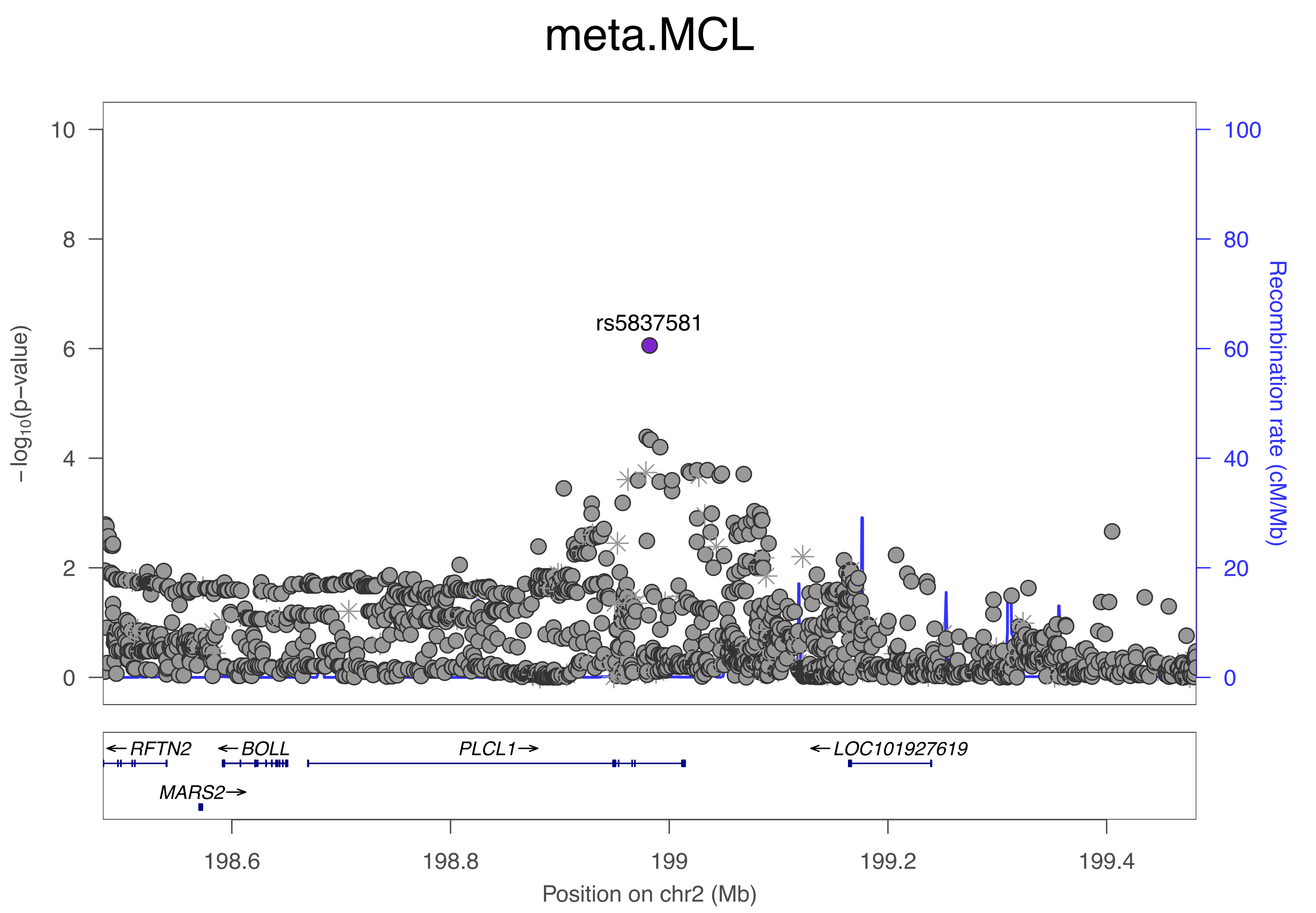

meta.MCL
rs5837581
−log10(p−value)
Recombination rate (cM/Mb)
0
2
4
6
8
10
0
20
40
60
80
100
RFTN2
BOLL
PLCL1
LOC101927619
MARS2
198.6
198.8
199
199.2
199.4
Position on chr2 (Mb)

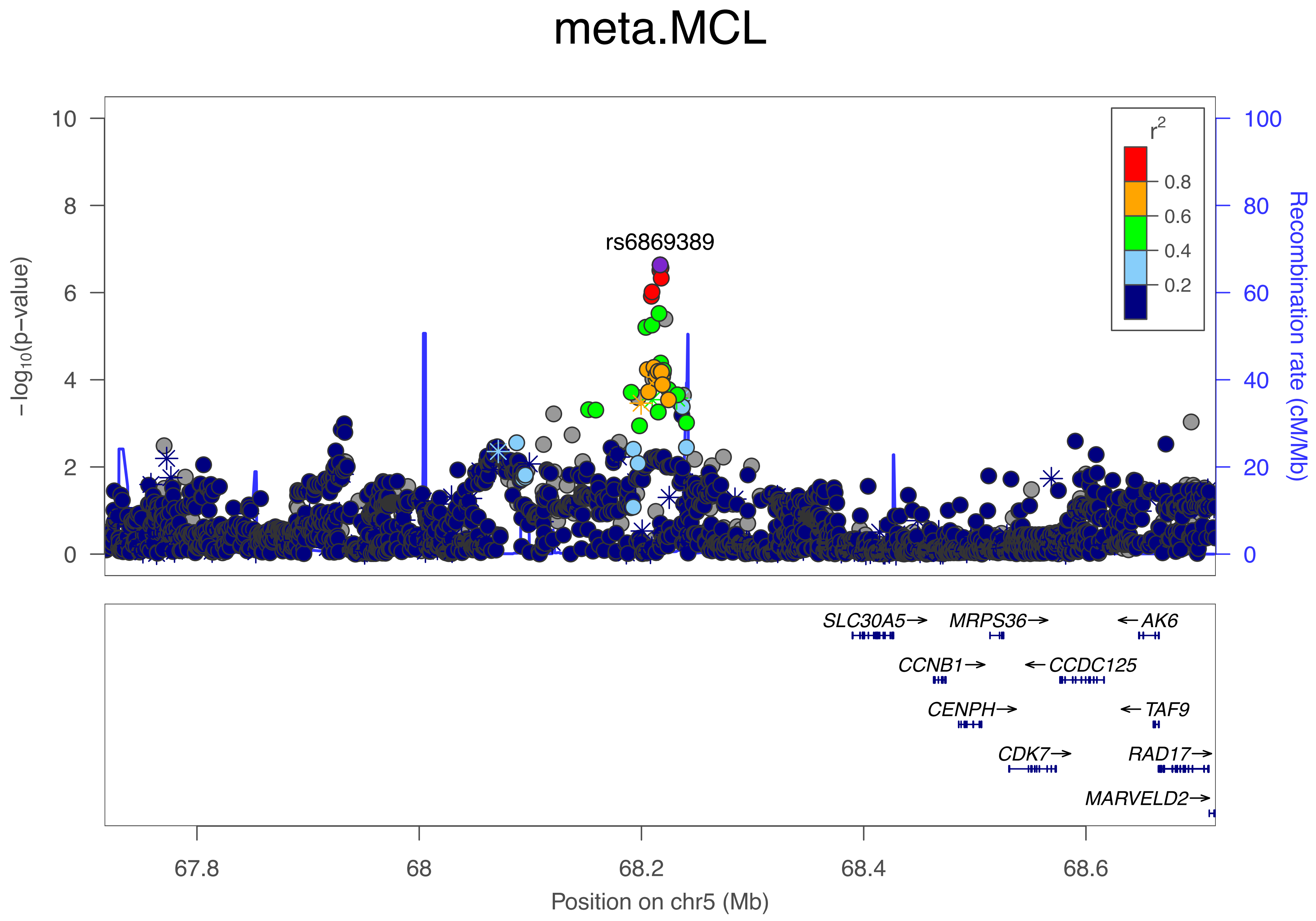
meta.MCL
rs6869389
r2
0.8
0.6
0.4
0.2
−log10(p−value)
Recombination rate (cM/Mb)
SLC30A5
MRPS36
AK6
CCNB1
CCDC125
CENPH
TAF9
CDK7
RAD17
MARVELD2
67.8
68
68.2
68.4
68.6
Position on chr5 (Mb)

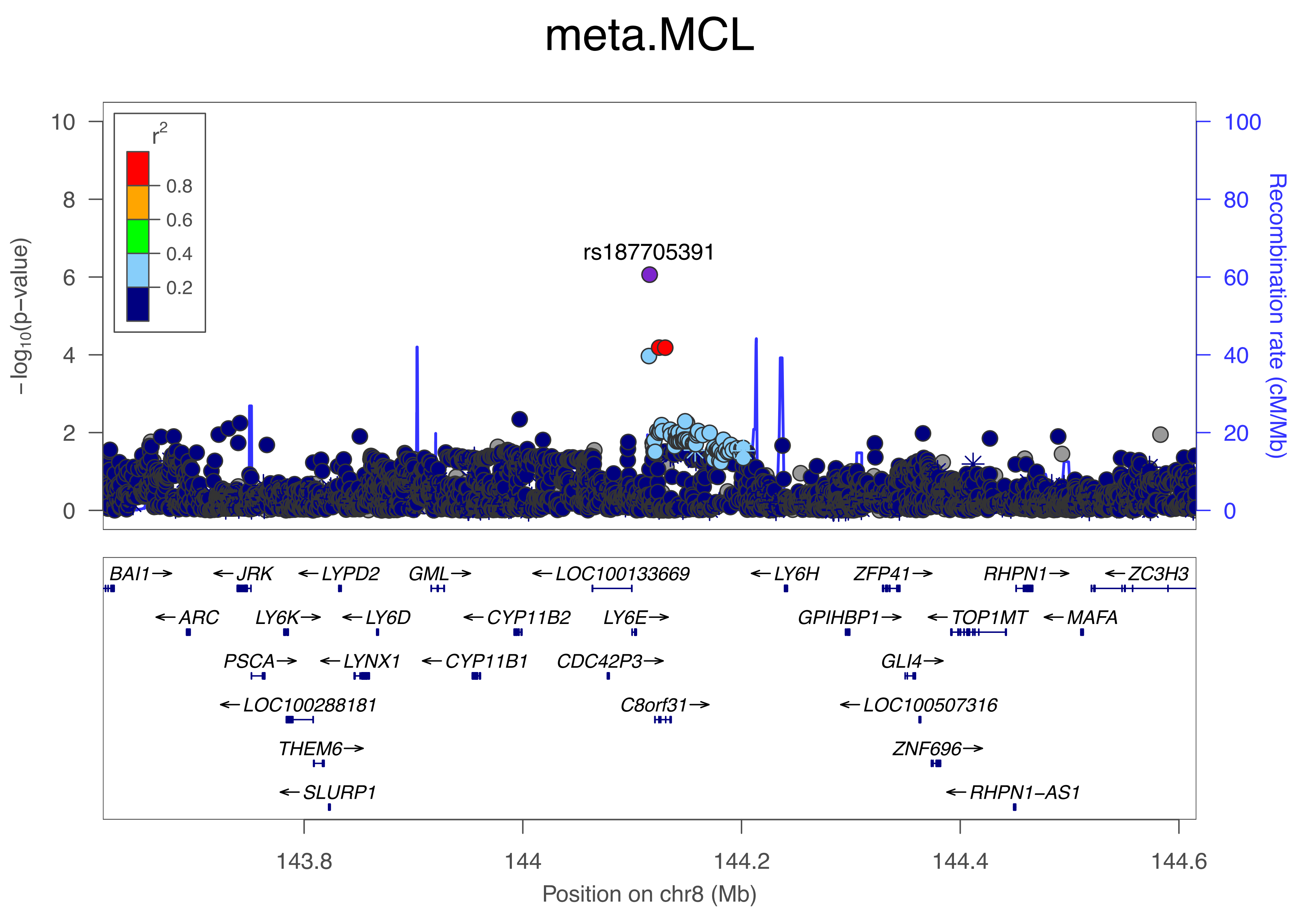
meta.MCL
rs187705391
r2
0.8
0.6
0.4
0.2
−log10(p−value)
Recombination rate (cM/Mb)
BAI1→
←JRK
←LYPD2
GML→
←LOC100133669
←LY6H
ZFP41→
RHPN1→
←ZC3H3
←ARC
LY6K→
←LY6D
←CYP11B2
LY6E→
GPIHBP1→
←TOP1MT
←MAFA
PSCA→
←LYNX1
←CYP11B1
CDC42P3→
GLI4→
←LOC100288181
C8orf31→
←LOC100507316
THEM6→
ZNF696→
←SLURP1
←RHPN1−AS1
143.8
144
144.2
144.4
144.6
Position on chr8 (Mb)

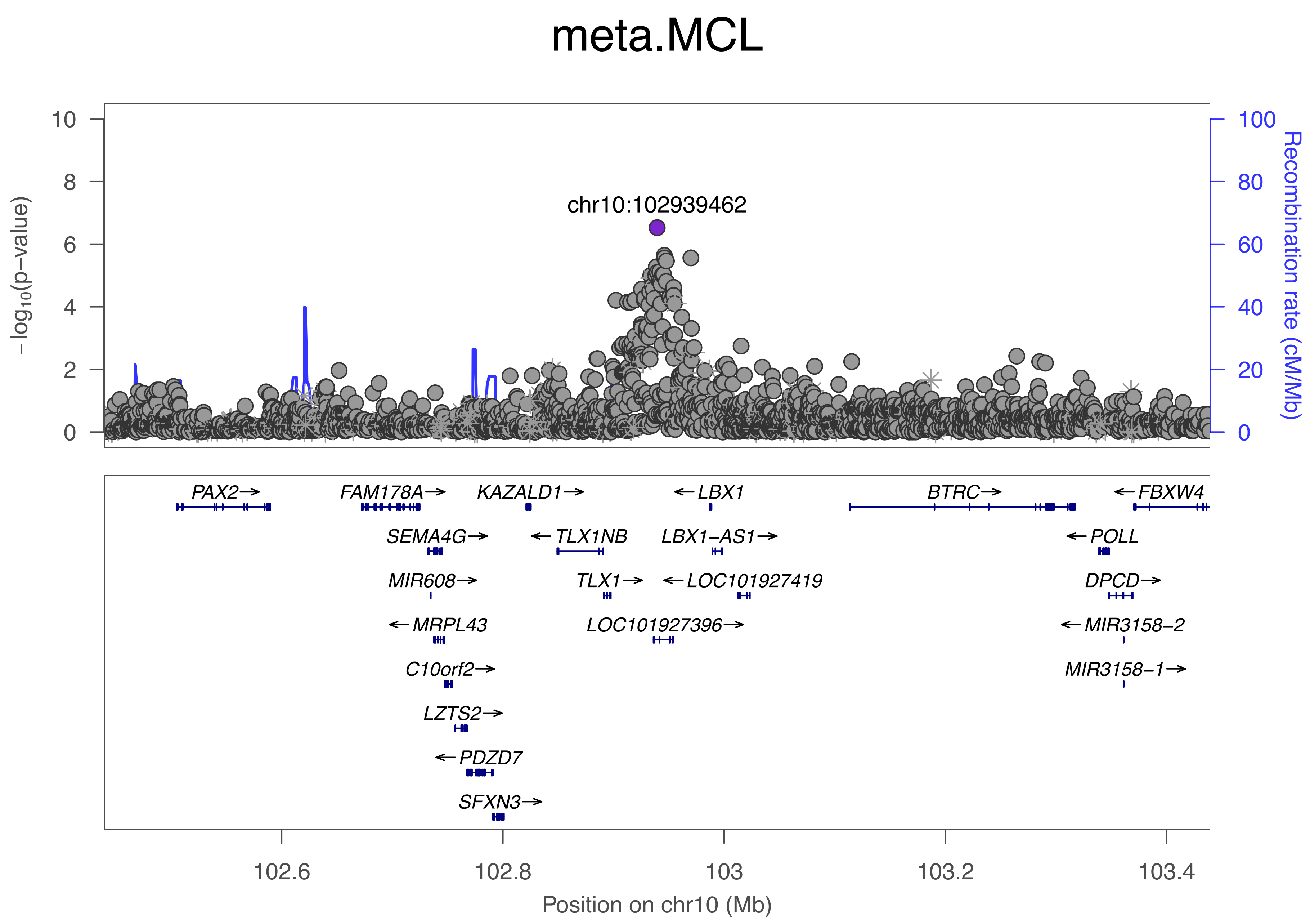

meta.MCL
chr10:102939462
-log10(p-value)
Recombination rate (cM/Mb)
10
8
6
4
2
0
100
80
60
40
20
0
PAX2→
FAM178A→
KAZALD1→
←LBX1
BTRC→
←FBXW4
SEMA4G→
←TLX1NB
LBX1-AS1→
←POLL
MIR608→
TLX1→
←LOC101927419
DPCD→
←MRPL43
LOC101927396→
←MIR3158-2
C10orf2→
MIR3158-1→
LZTS2→
←PDZD7
SFXN3→
102.6
102.8
103
103.2
103.4
Position on chr10 (Mb)

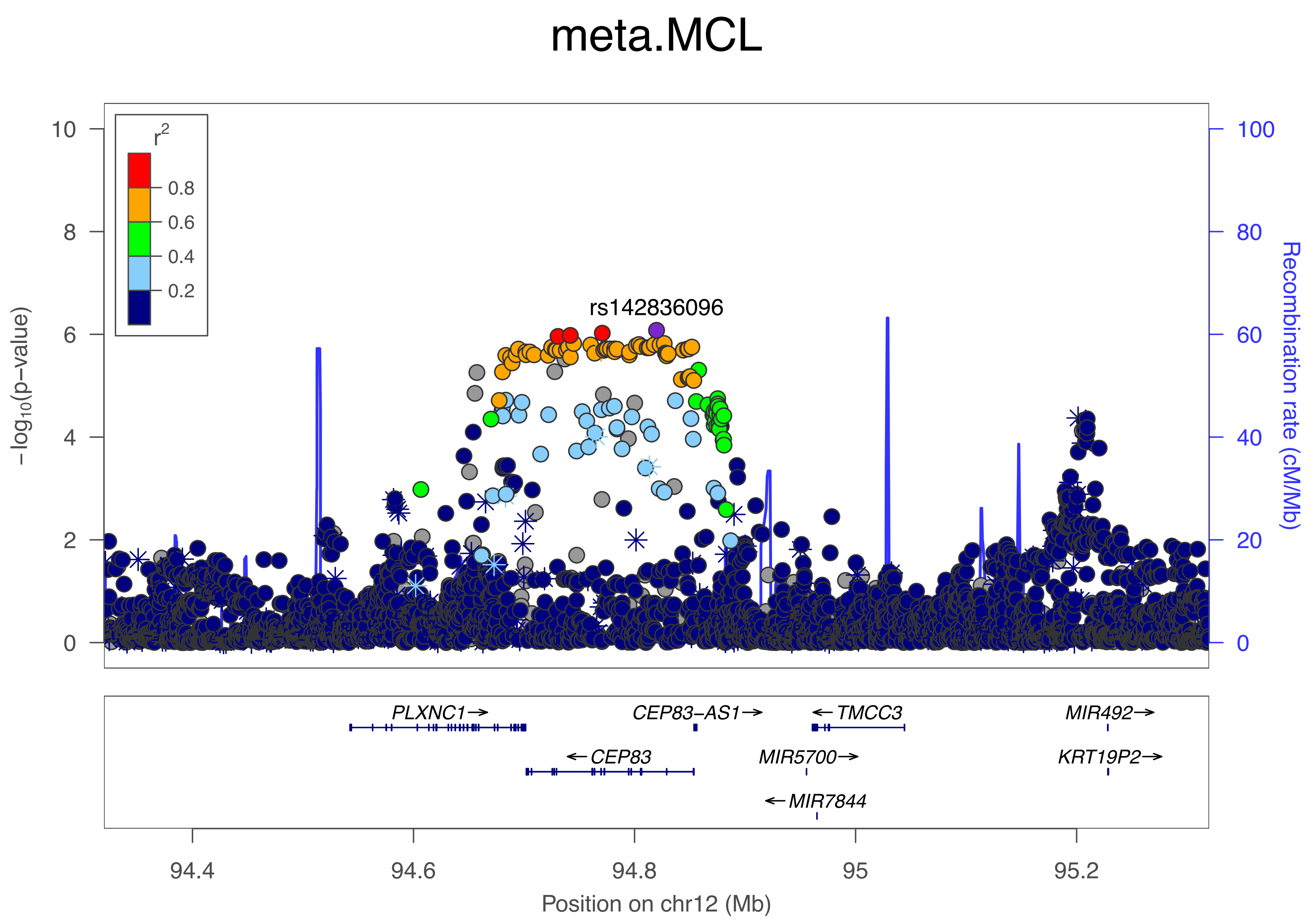

meta.MCL
r2
0.8
0.6
0.4
0.2
rs142836096
-log10(p-value)
Recombination rate (cM/Mb)
PLXNC1→
CEP83-AS1→
←TMCC3
MIR492→
←CEP83
MIR5700→
KRT19P2→
←MIR7844
94.4
94.6
94.8
95
95.2
Position on chr12 (Mb)

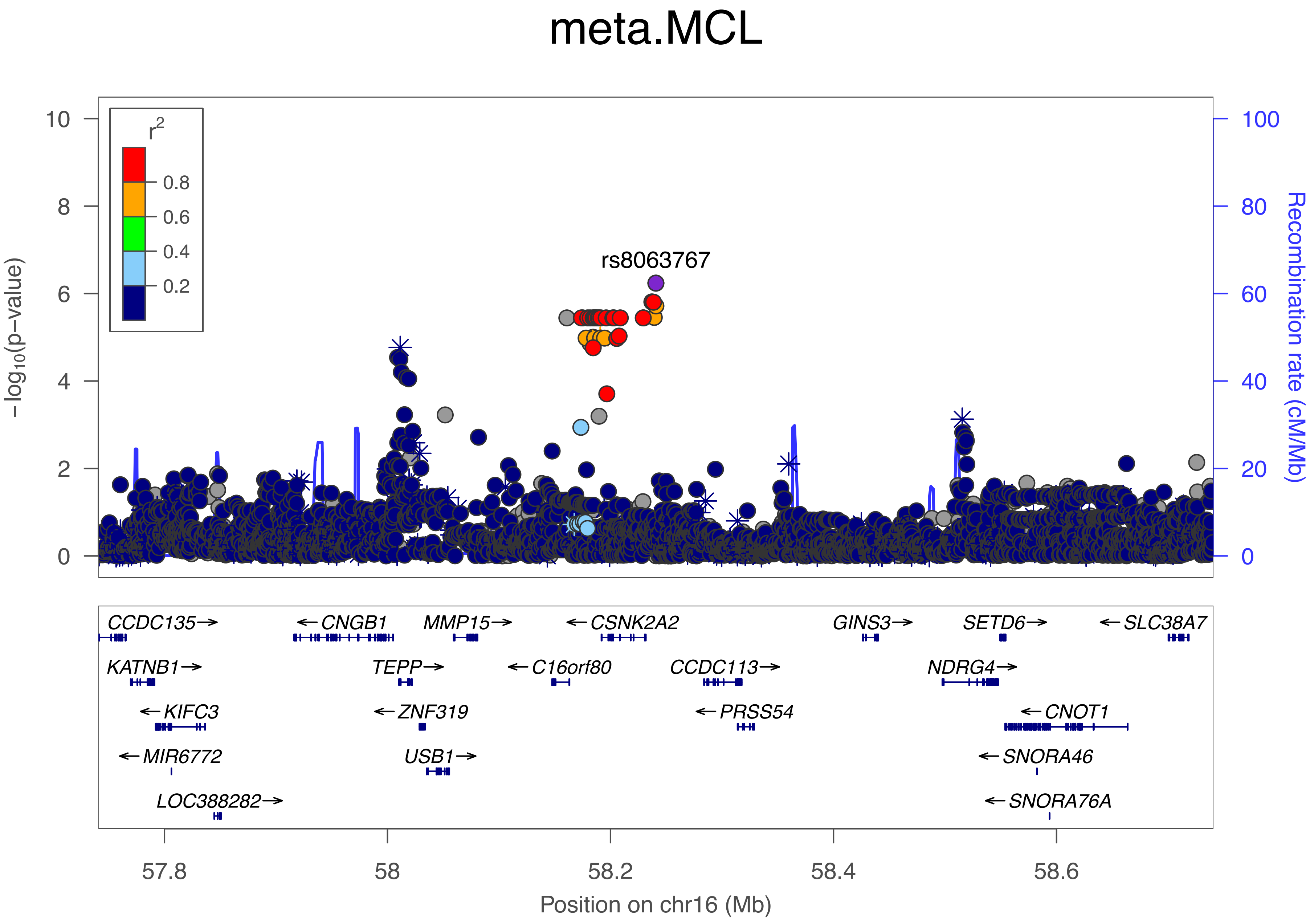

meta.MCL
rs8063767
r2
0.8
0.6
0.4
0.2
−log10(p−value)
Recombination rate (cM/Mb)
0
2
4
6
8
10
0
20
40
60
80
100
CCDC135→
←CNGB1
MMP15→
←CSNK2A2
GINS3→
SETD6→
←SLC38A7
KATNB1→
TEPP→
←C16orf80
CCDC113→
NDRG4→
←KIFC3
←ZNF319
←PRSS54
←CNOT1
←MIR6772
USB1→
←SNORA46
LOC388282→
←SNORA76A
57.8
58
58.2
58.4
58.6
Position on chr16 (Mb)

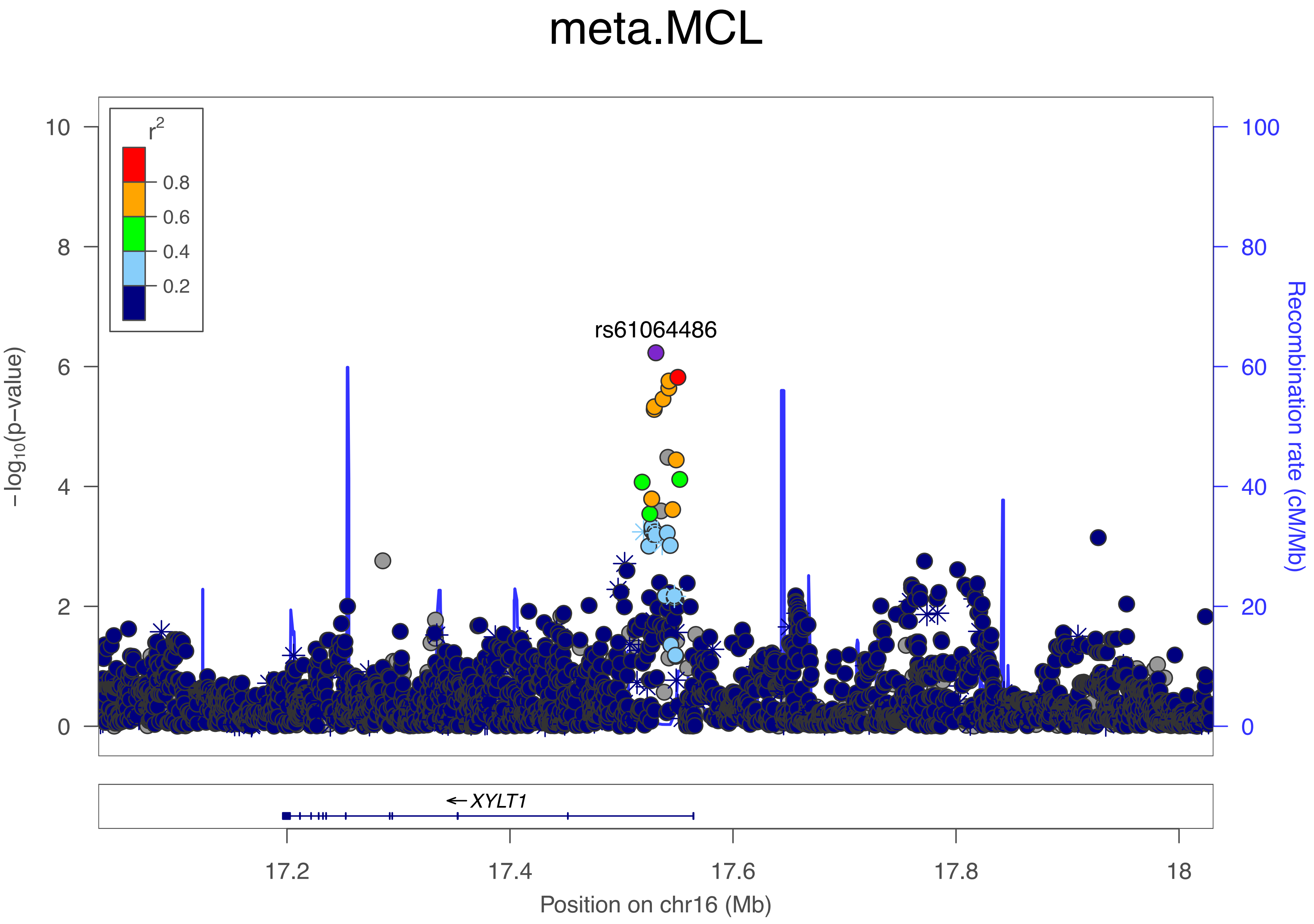
meta.MCL
r2
0.8
0.6
0.4
0.2
rs61064486
-log10(p-value)
Recombination rate (cM/Mb)
0
2
4
6
8
10
0
20
40
60
80
100
XYLT1
17.2
17.4
17.6
17.8
18
Position on chr16 (Mb)

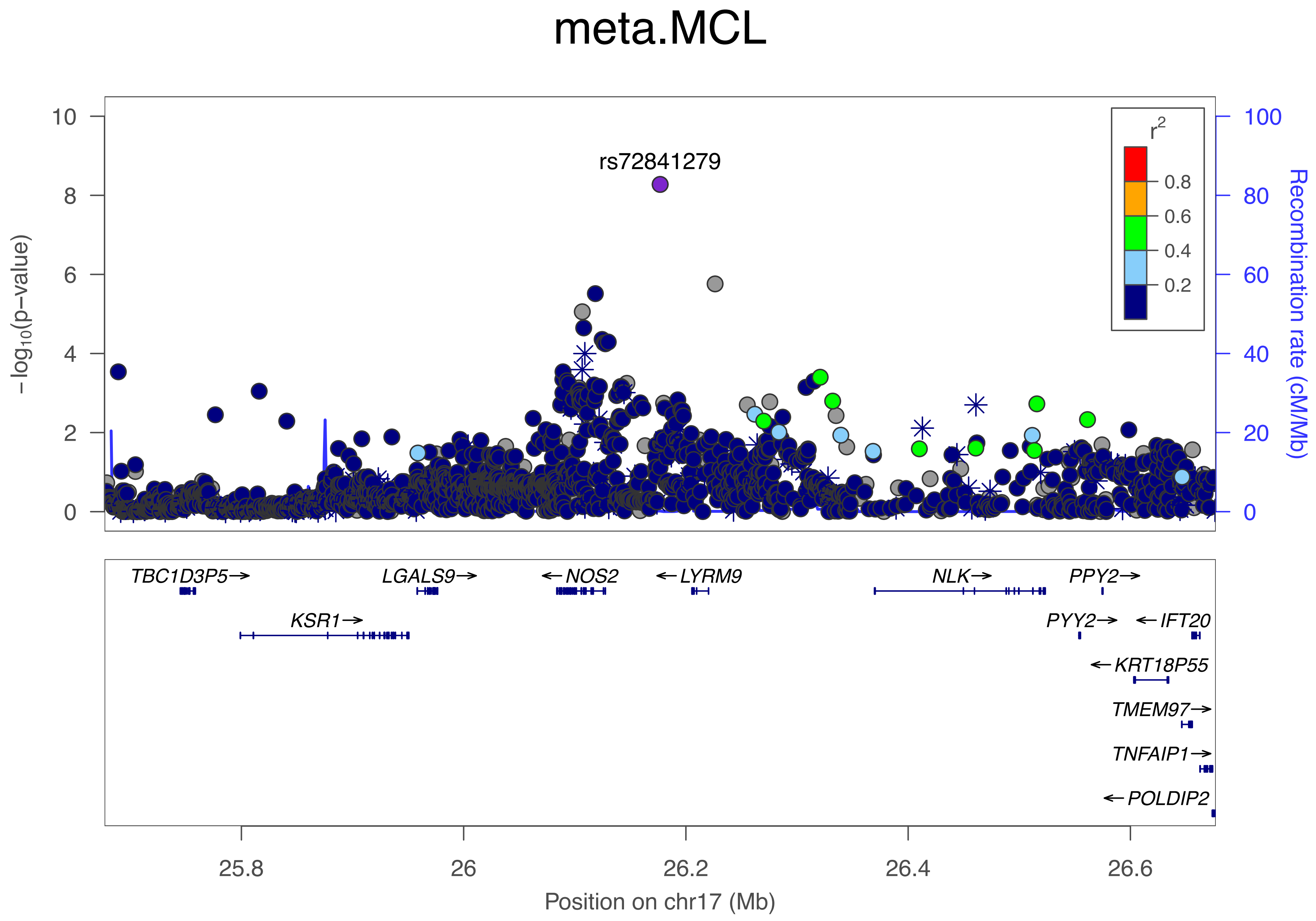

meta.MCL
rs72841279
r2
0.8
0.6
0.4
0.2
−log10(p−value)
10
8
6
4
2
0
100
80
60
40
20
0
Recombination rate (cM/Mb)
TBC1D3P5→
LGALS9→
←NOS2
←LYRM9
NLK→
PPY2→
KSR1→
PYY2→
←IFT20
←KRT18P55
TMEM97→
TNFAIP1→
←POLDIP2
25.8
26
26.2
26.4
26.6
Position on chr17 (Mb)

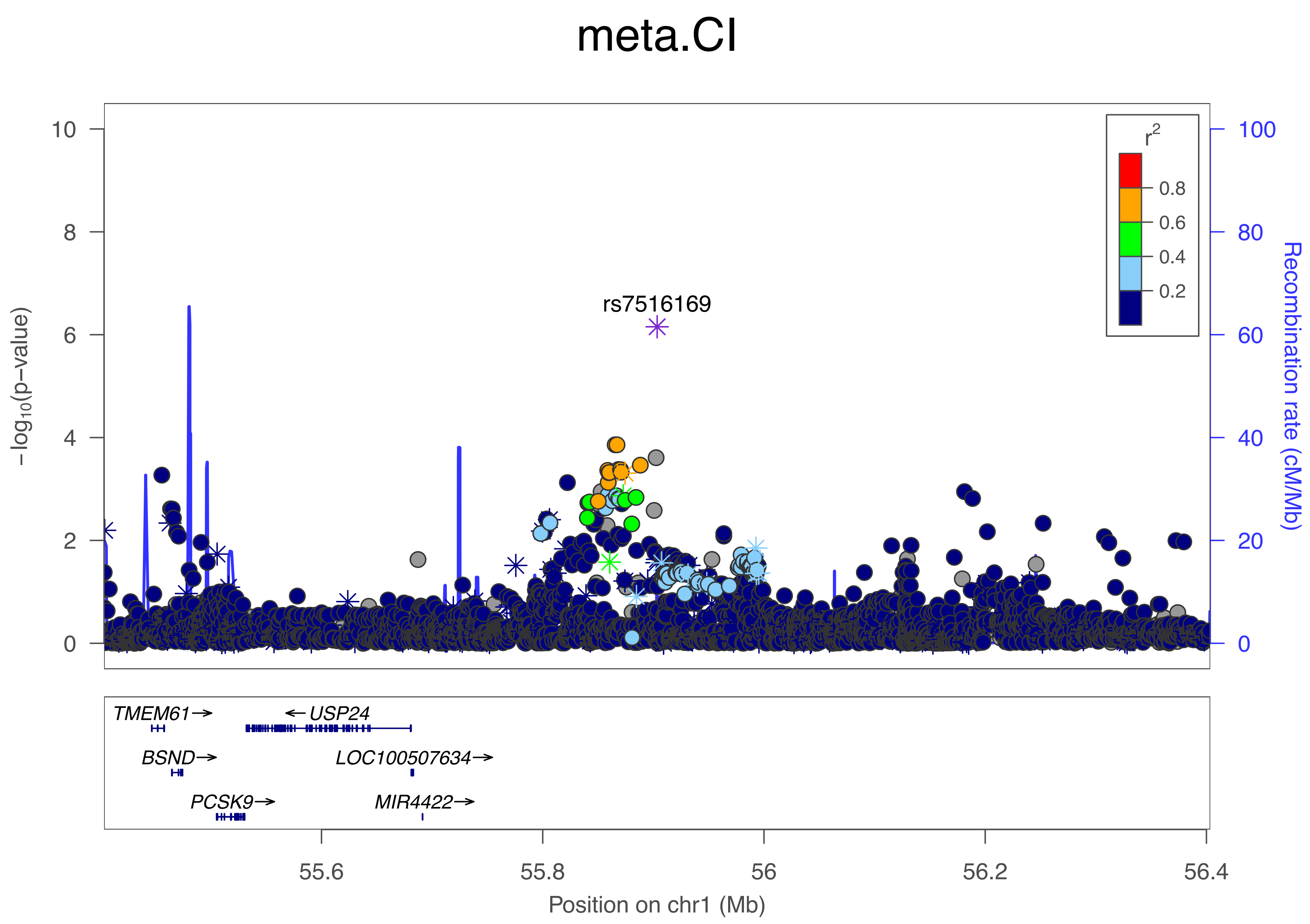
meta.CI
rs7516169
r2
0.8
0.6
0.4
0.2
−log10(p−value)
Recombination rate (cM/Mb)
10
8
6
4
2
0
100
80
60
40
20
0
TMEM61→
←USP24
BSND→
LOC100507634→
PCSK9→
MIR4422→
55.6
55.8
56
56.2
56.4
Position on chr1 (Mb)
